# Large-scale geographic patterns and environmental and anthropogenic drivers of wetland plant diversity in the Qinghai-Tibet Plateau

**DOI:** 10.1186/s12862-024-02263-w

**Published:** 2024-06-03

**Authors:** Yigang Li, Fan Liu, Yadong Zhou, Xing Liu, Qingfeng Wang

**Affiliations:** 1 College of Life Sciences and Technology, Hubei Engineering University, Xiaogan, China; 2 Laboratory of Extreme Environment Biological Resources and Adaptive Evolution, School of Ecology and Environment, Tibet University, Lhasa, China; 3 Wuhan Botanical Garden, Chinese Academy of Sciences, Wuhan, China; 4 School of Life Sciences, Nanchang University, Nanchang, Jiangxi China; 5 Key Laboratory of Biodiversity and Environment on the Qinghai-Tibet Plateau, Ministry of Education, College of Life Sciences, Wuhan University, Wuhan, China

**Keywords:** Functional diversity, Wetland plants, Qinghai-Tibet Plateau, Phylogenetic structure, Functional structure, Geographic pattern

## Abstract

**Background:**

The geographic patterns of plant diversity in the Qinghai-Tibet Plateau (QTP) have been widely studied, but few studies have focused on wetland plants. This study quantified the geographic patterns of wetland plant diversity in the QTP through a comprehensive analysis of taxonomic, phylogenetic and functional indices.

**Methods:**

Based on a large number of floras, monographs, specimens and field survey data, we constructed a comprehensive dataset of 1,958 wetland plant species in the QTP. Species richness (SR), phylogenetic diversity (PD), functional diversity (FD), net relatedness index (NRI) and net functional relatedness index (NFRI) were used to assess the taxonomic, phylogenetic and functional diversity of wetland plants. We explored the relationships between the diversity indices and four categories of environmental variables (i.e. energy-water, climate seasonality, topography and human activities). We used four diversity indices, namely endemic species richness, weighted endemism, phylogenetic endemism and functional endemism, together with the categorical analysis of neo- and paleo-endemism (CANAPE), to identify the endemic centers of wetland plants in the QTP.

**Results:**

SR, PD and FD were highly consistent and showed a decreasing trend from southeast to northwest, decreasing with increasing elevation. The phylogenetic structure of wetland plant assemblages in most parts of the plateau is mainly clustered. The functional structure of wetland plant assemblages in the southeast of the plateau is overdispersed, while the functional structure of wetland plant assemblages in other areas is clustered. Energy-water and climate seasonality were the two most important categories of variables affecting wetland plant diversity. Environmental variables had a greater effect on the functional structure of wetland plants than on the phylogenetic structure. This study identified seven endemic centres, mainly in the Himalayas and Hengduan Mountains.

**Conclusions:**

Climate and topography are the main factors determining the geographic distribution of wetland plant diversity at large scales. The majority of grid cells in the QTP with significant phylogenetic endemism were mixed and super-endemism. At large scales, compared to climate and topography, human activities may not have a negative impact on wetland plant diversity in the QTP.

**Supplementary Information:**

The online version contains supplementary material available at 10.1186/s12862-024-02263-w.

## Background

Understanding spatial patterns of biodiversity and their drivers along geographic gradients is a fundamental question in ecology and biogeography [1–3]. Studies have shown that patterns of diversity along environmental gradients can be influenced by the evolutionary history and functional trait composition of species assemblages [4–6]. Under the assumption of phylogenetic niche conservatism, closely related species may be ecologically similar, so that shared ancestry in assemblages may provide a surrogate for ecological similarity among co-occurring species [7]. However, niche conservatism is not the rule and phylogenetic diversity cannot be reduced to a proxy for current ecological niche diversity [7]. Functional traits related to behaviour, morphology, physiological strategies, and adaptation can represent the ability of species to utilize resources and capture ecological needs [2, 8]. Along biogeographic gradients, functional traits of species assemblages associated with different environmental conditions may reflect environmental filtering and ecological adaptation, and further influence ecosystem function [2]. Phylogenetic patterns reflect the influence of evolution and biogeographic history on assemblages structure, whereas functional patterns are more directly related to current ecological processes [9–11]. The systematic study of the relationships between species, phylogeny and function within a region helps to reveal the mechanisms that generate and maintain patterns of biodiversity [4].

Environmental filtering and competitive interactions among species are two widely studied processes in community assembly. In general, environmental stress acts as a filter, allowing species with similar functional traits adapted to the environment to persist in the community, resulting in functional clustering in the community. On the contrary, competition between species leads to the coexistence of species with different functional traits, resulting in functionally overdispersed assemblages [4, 12, 13]. It is generally assumed that closely related species may share common features, such as ecological niches and functional traits. If functional traits are highly conserved along the phylogeny, then phylogenetic structural patterns may also serve as a good proxy for functional structure. However, phylogenetic similarity cannot always represent ecological similarity [13]. The research shows that there are strong geographic differences in the phylogenetic and functional structures of birds, with the greatest differences in the temperate highlands [14]. There are regional differences in the functional structure and phylogenetic structure of Chinese pheasants, indicating that phylogenetic structure is not always a good proxy for identifying functional structure [13]. Compared to phylogenetic relationships, functional traits are more susceptible to environmental influences [4]. Therefore, we hypothesize that environmental variables will have different effects on the phylogenetic and functional structure of wetland plant assemblages.

Numerous studies have attempted to determine the impacts of various environmental variables that shape the distribution and diversity of organisms in different ecological regions [15, 16]. The energy-water hypothesis posits that the availability of water and energy determines the total resources available to plants that control biological activity, which in turn determines the changes in biodiversity [15, 17]. The habitat heterogeneity hypothesis posits that the presence of environmental or resource heterogeneity can generate greater ecological niche diversity, and that the existence of species depends on the available ecological niche space, allowing species to coexist over large spatial scales [15, 18]. The climate variability hypothesis posits that organisms that experience greater thermal variability have a wider range of physiological thermal tolerance and therefore tend to be geographically widespread [19]. Temperature seasonality is also known as the “temperature seasonality tolerance hypothesis” or the “climate variability hypothesis” [20]. Species that grow in seasonal regions tend to have larger ecological niches and ranges, reducing the risk of species extinction, but also reducing the opportunities for species formation [21]. Human activities can directly affect species distribution patterns and diversity at a regional scale through land-use change, disturbance, habitat fragmentation, destruction and loss [15, 22]. Overall, we believe that no single factor can fully explain the formation and maintenance of patterns of species diversity gradients. To better understand patterns of species diversity, it is necessary to consider multiple hypotheses or mechanisms [23]. Water, as an important component of wetland ecosystems, has a crucial influence on the species composition, diversity and structure of wetland vegetation [24]. Therefore, we hypothesise that energy-water environmental variables will have the greatest impact on wetland plant diversity, while human activity variables will be negatively correlated with wetland plant diversity.

Centers of endemism act as “hotspots within hotspots” and are considered one of the most attractive topics in conservation biogeography [25]. Endemism is often considered an important criterion for biodiversity conservation at global, national and even local levels [26]. Biodiversity hotspots are identified through hotspot analysis and protection of these hotspots is considered the most cost-effective conservation option. Therefore, hotspots based on different diversity indices should be considered when identifying priority conservation areas [27]. Phylogenetic endemism provides an alternative evolutionary metric by integrating phylogenetic diversity (PD) and the geographic distribution of species, allowing the identification of geographic areas where species are both phylogenetically and spatially restricted [28]. Based on a species distribution data set with 12,824 Chinese endemic plants, Huang et al. [26] have identified 19 centers of endemism, mainly located in mountainous regions, with the Hengduan Mountains being a critical area for conservation. Zhang et al. [29] conducted a spatial phylogenetic analysis of the Chinese angiosperm flora at the generic level and identified 9 centers of endemism, including the Xigazê and Lhoka prefecture in the Himalayas. Therefore, we speculate that the center of endemic wetland plants in the QTP may also be concentrated in the Himalayas and Hengduan Mountains region.

Known as the roof of the world, the QTP has an average elevation of over 4000 m and is relatively rich in biodiversity. The QTP supports the largest wetland groups in China [30], with plateau wetlands consisting mainly of wet meadows, marshlands, riverine and lacustrine wetlands [31]. The plateau wetland ecosystem supports the richest biodiversity in the world at high elevations and has the most important ecological barrier function. It is highly sensitive to global change and is considered a symbol of global change [32]. Wetlands in the QTP have the following characteristics. Firstly, wetlands are located in arid and semi-arid environments, which makes them vulnerable. In addition to the typical riverine and lacustrine wetlands, most of the marshlands have less water storage, with limited rainwater and runoff being used as supplementary sources, resulting in wetlands that are prone to drying out. Secondly, the plateau wetlands are an important production base for livestock farming, which is closely linked to human activities. Finally, the plateau wetlands are very sensitive to climate change. Although climate warming may accelerate the melting of snow and frozen soil and increase the supply of water resources in the wetland, the increase in evaporation causes water depletion [33].

The conservation of wetland plant diversity in fragile alpine wetland habitats is urgent, especially in the face of global climate change. Analysis of the spatial patterns of plant taxonomic, phylogenetic and functional structure will help us to understand the ecological and evolutionary factors that determine the composition of wetland communities in the QTP. The special wetland ecosystems of the QTP support a large number of wetland plants, but the patterns of plant diversity in the region have been better studied and fewer studies have focused on wetland plants [34–36]. Based on a large amount of literature, monographs, online databases and field survey data, this study comprehensively analysed the geographic distribution characteristics of wetland plants in terms of taxonomic, phylogenetic and functional diversity, as well as the effects of environmental factors and human activities on these distribution patterns. At the same time, distribution centers of endemic wetland plants in the QTP were identified. Specifically, we aim to answer the following questions: (1) Are there differences in the geographic patterns of wetland plant diversity in the QTP, particularly in the phylogenetic and functional structure of wetland plants? (2) Which environmental variables drive the geographic patterns of wetland plant diversity in the QTP? (3) Where are the main hotspots for endemic wetland plants in the QTP?

## Methods

### Study area and environmental factors

We used the dataset of urban land and urbanization index on the Tibetan Plateau (2018, 2019) to identify the geographic area of the QTP [37], which was downloaded from the National Tibetan Plateau Data Center (https://data.tpdc.ac.cn/). In order to eliminate the potential effect of area on species richness, the QTP was divided into 50 km × 50 km grid cells, and grid cells within China with less than 50% land area were excluded, resulting in a total of 1037 grid cells. A 50 km × 50 km grid was used in this study mainly because species distribution data are mainly recorded by county. The grid area is close to the median of the county area in the QTP region, which reduces the variability of distribution information and has been widely used in previous studies [34, 38]. We overlapped this grid map with DEM (at 250 m resolution) and the administrative map of the QTP to obtain the elevation ranges and counties included in each grid. Only when both the horizontal distribution (county) and vertical distribution (elevation range) of a species are included in a grid, will the species be recorded in the grid [34].

The Qinghai-Tibet Plateau (QTP), with its complex geological and tectonic evolutionary history, rich topography and diverse climatic conditions [39], provides a unique experimental site for studying large-scale patterns of species diversity and the mechanisms by which they are generated. The topography of the QTP is extremely complex, with dense mountains separated by deep valleys, resulting in a variety of climatic conditions throughout the plateau region [40]. From the south-east to the north-west of the plateau, temperature and rainfall gradually decrease and the climate changes from warm and humid to cold and dry [41]. The climate data used in the analysis include: annual mean temperature (AMT), annual precipitation (AP), potential evapotranspiration (PET), actual evapotranspiration (AET), precipitation of driest month (PDM), min temperature of coldest month (MTCM) and aridity index (AI). In previous research, these variables have also been used as surrogates for energy and water [15, 18, 42]. Temperature annual range (TAR), mean diurnal range (MDR), temperature seasonality (TS), and precipitation seasonality (PS), which are commonly used as surrogates for climate seasonality [15, 18]. The data of annual mean temperature (AMT), annual precipitation (AP), precipitation of driest month (PDM), min temperature of coldest month (MTCM), temperature annual range (TAR), mean diurnal range (MDR), temperature seasonality (TS) and precipitation seasonality (PS) were extracted from the WorldClim database ( http://www.worldclim.org). The data of PET and AI were downloaded from the Version 3 of the Global Aridity Index and Potential Evapotranspiration Database of the Plant Science Data Center (https://www.plantplus.cn/cn). AET data was from the Global-PET database (www.cgiar-csi.org). Human Footprint data were used to represent the impact of human activities. The data are calculated from six spatial data representing human activities, including population density, land use, grazing density, night lighting, railways and highways [43]. The human footprint data was downloaded from the National Tibetan Plateau/Third Pole Environment Data Center (https://data.tpdc.ac.cn/).

We used elevation variation coefficient (EVC), slope (Slope), and wetland area (WA) to represent topographic variables. Wetland area (WA) represents the total area of rivers, lakes and swamp wetlands in each grid. We downloaded a 30-m spatial distribution dataset of Chinese vegetated wetlands from the National Earth System Science Data Center (http://www.geodata.cn). We used ArcGIS to extract water surface data in China from the 1:250000 national basic geographic database, which was downloaded from the National Geomatics Center of China (NGCC, http://www.ngcc.cn/) [44]. The elevation variation coefficient (EVC) is the ratio of the standard deviation of the elevation to the mean in the grid unit and reflects the relative change in surface elevation. DEM data was downloaded from the Resource and Environmental Science and Data Center (https://www.resdc.cn/). Slope was extracted from the slope map of the Tibet Plateau downloaded from the National Tibetan Plateau/Third Pole Environment Data Center (https://data.tpdc.ac.cn/). ArcGIS 10.2 was used to extract the elevation of each grid cell and calculate the values of energy-water, climate seasonality, human activities and topographic environmental variables (Additional file 1). To reduce the effects of multicollinearity, Pearson correlation analysis was used to screen environmental variables with high collinearity (r > |0.8|) in combination with their ecological significance. Finally, eleven environmental variables were selected for further analysis.

### Distribution and functional data of wetland plants

We have compiled a checklist of 1958 wetland plants in the QTP based on extensive floras, monographs and online sources (Additional file 2), including 100 aquatic plants and 1858 hygrophytes (Additional file 3). Aquatic plants, which include emergent, floating-leaved plants, floating plants and submerged plants, are those that live in water. Hygrophytes are plants that grow on riverbanks, lake shores, stream banks, ditch sides and marshes and which cannot tolerate prolonged water shortages or seasonal flooding. Exotic, invasive and planted wetland plants were excluded from this study and the infraspecific taxa were combined with their respective species. We used extensive specimens, floras, monographs and online database data, as well as field survey data from 2018 to 2020, to ensure that species distribution information was as comprehensive and accurate as possible. Meanwhile, based on floras and specimens, we identified six morphological traits of each species: growth form, fruit type, maximum height, maximum leaf size, flower colour and flowering period length [4, 45–47]. The growth forms are divided into emergent, floating, submerged, floating-leaved and hygrophytes. Fruit types included capsule, achene, nutlet, schizocarp, caryopsis, follicle, silicle, legume, berry, drupe, utricle, silique, nut, caryopsis, achene. The maximum height and flowering period are determined by the recorded values of Flora Reipublicae Popularis Sinicae, Flora of Tibet, Flora of Qinghai and plant specimens. Three formulas were used to estimate the maximum leaf size: length × breadth × 2/3 for entire or dentate leaves; length × breadth× 1/2 for shallow-lobed leaves; and length × breadth × 1/3 for deep-lobed leaves [4, 48]. The compound leaf is represented by the largest leaflet, which is considered a single leaf in our analysis. The colors of flowers are classified as purple, yellow, green, red, white, brown, blue and black.

### Phylogeny and function construction of wetland plants

Species names were standardized mainly according to “The Plant List” version 1.1 (http://www.theplantlist.org), using the package “U.Taxonstand” [49]. The phylogenetic tree of wetland plants was generated from the largest dated mega-tree using the R package “U.PhyloMaker” [50] based on GBOTB.extended.TPL.tre and build.nodes.1 [51]. For species absent from the mega-tree, we used the Scenario S3 approach to add them to their families and genera in the mega-tree [52] (Additional file 4). We used the “FD” package [53] to calculate the trait-based Gower distance matrix based on six functional traits and performed hierarchical clustering analysis to produce a trait tree [4] (Additional file 5).

### Taxonomic, phylogenetic and functional diversity

Species richness (SR) is defined as the total number of wetland plant species found in each grid. Phylogenetic diversity (PD) is a commonly used measure for the phylogenetic diversity of species assemblages. Phylogenetic diversity (PD) measures the sum of all phylogenetic branch lengths that connect species in a species assemblage. The net relatedness index (NRI) and the net nearest taxon index (NTI) are the most commonly used indices to measure the phylogenetic structure of each site, reflecting primarily the community structure in the deeper and shallower parts of a phylogeny, respectively [54]. In this study, the net relatedness index (NRI), which reflects deep phylogenetic structure, was selected for analysis, defined as:

$$NRI{\rm{ }} = {\rm{ }} - 1{\rm{ }} \times {\rm{ }}\left( {MP{D_{observed}}-{\rm{ }}MP{D_{randomized}}} \right)/sdMP{D_{randomized}}$$

where MPD_observed_ is the observed mean phylogenetic distance, MPD_randomized_ is the expected mean phylogenetic distance of randomized assemblages, and sdMPD_randomized_ is the standard deviation of mean phylogenetic distance for the randomized assemblages. A positive NRI indicates phylogenetic clustering, while a negative value indicates phylogenetic dispersion [55]. As a functional equivalence to PD and NRI, we calculated FD and NFRI [56]. All diversity indices were calculated using the R package “phylomemeasures” [57].

### Identification of endemic plant distribution centers and conservation status in the QTP

In this study, endemic species richness, weighted endemism, phylogenetic endemism and functional endemism of wetland plants in the QTP were analysed together. Endemic species richness was defined as the total number of endemic species found in each grid cell. Based on the checklist of endemic seed plant species in the QTP reported by Yu et al. [58], we selected from the checklist of wetland plants in the QTP, resulting in a total of 395 endemic wetland plants in the QTP. Weighted endemism of wetland plants was calculated for each grid cell, using an arbitrary region or range size threshold to define what constitutes a species [59, 60]. Phylogenetic endemism is the total phylogenetic branch length encompassed by wetland plant species in the QTP, where the phylogeny is modified so that each branch length is divided by the global range size of its descendant clade [61]. Functional endemism combines information on functional traits with the size of the geographic range to extend the measure of endemism for functional diversity in the same way that phylogenetic endemism extends the measure of phylogenetic diversity [62]. Phylogenetic endemism and functional endemism were calculated using the R package “PDcalc” [63]. We applied a method developed by Mishler et al. [64] called categorical analysis of neo- and paleo-endemism (CANAPE). This method allows the identification of five types of grid cells: nonsignificant, centers of neo-endemism, centers of paleo-endemism, centers of mixed-endemism and super-endemism. The analysis was calculated using Biodiverse 2.0 software [65].

In this study, hotspots of endemic wetland plant diversity are identified as follows: The grid cells with the top 10% values of endemic richness, weighted endemism, phylogenetic endemism and functional endemism [27] are integrated with the centers of neo-endemism, centers of paleo-endemism, centers of mixed-endemism and super-endemism grid cells identified by the CANAPE analysis to serve as hotspots of endemic wetland plant diversity in the QTP. To assess the conservation status of hotspots of endemic wetland plant diversity, we overlaid digital maps of protected areas in the QTP with maps of wetland plants [27]. This allowed us to identify conservation gaps. Data for nature reserves in the QTP were obtained from Liu et al. [66].

### Data analysis

First, we used ArcGIS 10.2 [67] to create maps of geographic patterns of SR, PD, FD, NRI and NFRI for wetland plants in the QTP. The diversity index values were then classified into ten categories using the natural breaks classification method. In this study, the linear relationship between SR, PD, FD, NFRI and elevation was investigated using ordinary least squares (OLS). Generalized additive model (GAM) was used to explore the relationship between NRI and elevation, which is a nonparametric extension of the generalized linear model and can model nonlinear relationships [68]. In this study, the relationship between diversity indices and environmental variables was assessed in three steps to determine which environmental variables primarily affect wetland plant diversity. As spatial autocorrelation can affect the explanatory power of regression models, the relationship between environmental variables and wetland plant diversity indices was first analysed using single variable ordinary least squares (OLS) and spatial simultaneous autoregressive error (SAR) models. Then, the relationship between wetland plant diversity indices and environmental conditions was studied by redundancy analysis (RDA). Finally, we grouped all environmental variables into four categories: energy-water, climate seasonality, human activities and topography. We used variance partitioning analysis to examine the contribution of independent effects of the energy-water, climate seasonality, human activities and topography variables, and their overlapping contributions to SR, PD, FD, NRI and NFRI. We use the R package “vegan” for RDA and variance partitioning analysis [69]. Spatial autoregressive models were constructed using Spatial Analysis in Macroecology (SAM) [70]. All analyses except spatial autoregression were carried out using the R 3.3.3 [71].

## Results

### Geographic pattern of wetland plant diversity

**Fig. 1:**
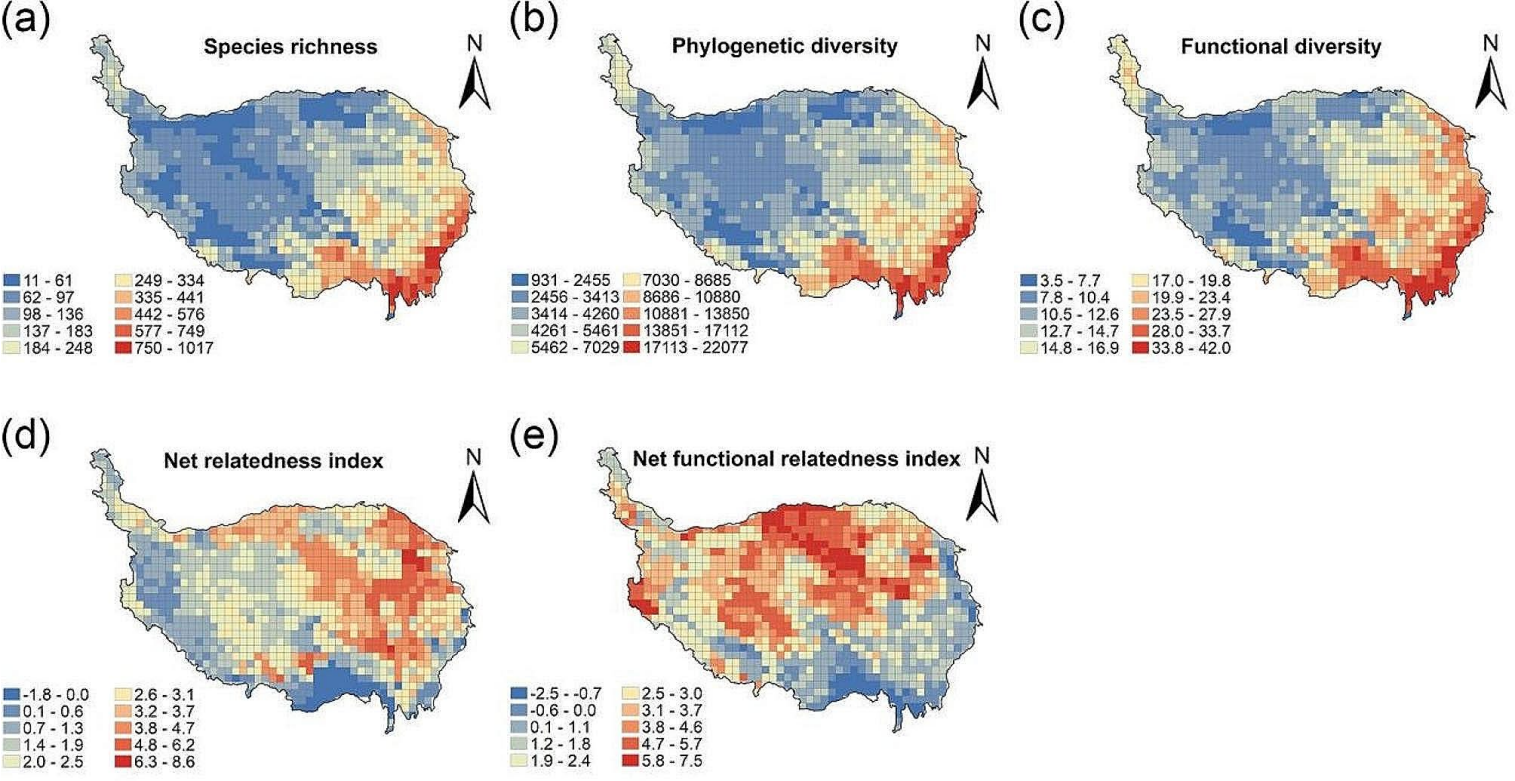
Geographic patterns of species richness (SR) (**a**), phylogenetic diversity (PD) (**b**), functional diversity (FD) (**c**), net relatedness index (NRI) (**d**) and net functional relatedness index (NFRI) (**e**) of wetland plants in the Qinghai-Tibet Plateau. The geographic boundaries of the Qinghai-Tibet Plateau were sourced from the dataset of urban land and urbanization index on the Tibetan Plateau (2018, 2019)

The SR showed a declining trend from southeast to northwest of the QTP, with the highest SR concentrated in the Hengduan Mountains and Himalayas in the southeastern part of the plateau (Fig. 1a). PD and FD showed the same spatial pattern as SR. NRI showed a spatial pattern of high in the northeast and low in the southwest (Fig. 1b, c). The positive NRI values in most areas of the QTP indicated that the phylogenetic structure of wetland plant assemblages in the QTP was predominantly clustered. Furthermore, the NRI showed an increasing trend from the southwest to the northeast, indicating that the phylogenetic clustering of wetland plant assemblages was higher in the northeast than in the southwest (Fig. 1d). NFRI showed the opposite trend to SR, PD and FD, increasing from southeast to northwest. The areas with relatively low NFRI values were mainly concentrated in the Hengduan Mountains and the Himalayas in the southeast of the plateau, indicating that the functional structure of wetland plant assemblages in these regions was overdispersed, while the functional structure of wetland plant assemblages in other parts of the plateau was clustered (Fig. 1e).

## Elevational pattern of diversity indices

**Fig. 2:**
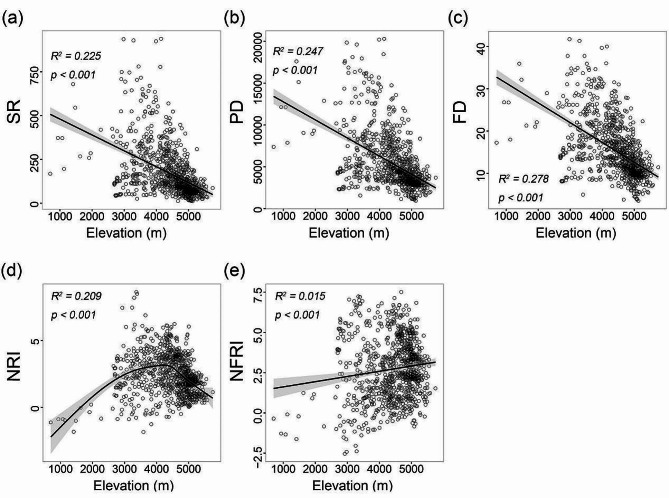
The elevational patterns of species richness (SR) (**a**), phylogenetic diversity (PD) (**b**), functional diversity (FD) (**c**), net relatedness index (NRI) (**d**) and net functional relatedness index (NFRI) (**e**) of wetland plants in the Qinghai-Tibet Plateau. Black lines were fitted based on the ordinary least squares (OLS) or Generalized additive model (GAM) models, and shaded areas represent 95% confidence intervals (*N* = 1037)

The SR, PD and FD of wetland plants showed the same elevational pattern, gradually decreasing with increasing elevation (Fig. 2a, b, c). The NRI showed a hump-shaped change along the elevational gradient, with the maximum value appearing at around 4000 m, indicating that that the phylogenetic structure of wetland plant assemblages is more clustered in mid-elevation areas (Fig. 2d). The elevational pattern of NFRI was inconsistent with that of NRI and increased monotonically with increasing elevation, indicating that the functional structure of wetland plant assemblages tended to be more clustered at higher elevations (Fig. 2e).

### Driving factors of wetland plant diversity

The results of single variable ordinary least squares (OLS) and spatial simultaneous autoregressive error (SAR) models indicate that SR, PD and FD are significantly positively correlated with annual mean temperature (AMT), annual precipitation (AP), precipitation of the driest month (PDM), slope (Slope), elevation variation coefficient (EVC) and human footprint (HF). Conversely, they are significantly negatively correlated with potential evapotranspiration (PET), mean diurnal range (MDR), temperature seasonality (TS) and precipitation seasonality (PS). The NRI is significantly negatively correlated with annual mean temperature (AMT), precipitation of driest month (PDM), potential evapotranspiration (PET), slope (Slope), elevation variation coefficient (EVC) and human footprint (HF), but positively correlated with mean diurnal range (MDR), temperature seasonality (TS), wetland area (WA) and human footprint (HF). Finally, the NFRI is significantly negatively correlated with annual mean temperature (AMT), annual precipitation (AP), precipitation of driest month (PDM), slope (Slope), elevation variation coefficient (EVC) and human footprint (HF), but positively correlated with potential evapotranspiration (PET), mean diurnal range (MDR), temperature seasonality (TS), precipitation seasonality (PS) and wetland area (WA) (Table 1).

**Table 1:** Results of the single variable ordinary least squares (OLS) models and spatial simultaneous autoregressive error (SAR) models of the associations between wetland plant species richness (SR), phylogenetic diversity (PD), functional diversity (FD), net relatedness index (NRI) and net functional relatedness index (NFRI) and the four categories of environmental factors. *R*^2^ and coefficients (coef) for the OLS models and SAR models are listed. * *p* < 0.05; ** *p* < 0.01; *** *p* < 0.001

		AMT	AP	PDM	PET	TS	PS	MDR	Slope	EVC	WA	HF
**SR**	**Coef**_**OLS**_	0.157^***^	0.208^***^	0.149^***^	-0.111^***^	-0.157^***^	-0.098^***^	-0.168^***^	0.181^***^	0.145^***^	-0.012	0.164^***^
	***R*** ^**2**^_**OLS**_	0.232	0.408	0.210	0.117	0.232	0.092	0.267	0.309	0.199	0.001	0.254
	**Coef**_**SAR**_	0.072^***^	0.110^***^	0.085^***^	-0.026^***^	-0.151^***^	-0.061^***^	-0.09^***^	0.095^***^	0.074^***^	-0.005	0.058^***^
	***R*** ^**2**^_**SAR**_	0.410	0.470	0.440	0.378	0.499	0.412	0.433	0.434	0.379	0.358	0.428
**PD**	**Coef**_**OLS**_	0.124^***^	0.155^***^	0.115^***^	-0.073^***^	-0.126^***^	-0.076^***^	-0.127^***^	0.141^***^	0.116^***^	-0.019^**^	0.117^***^
	***R*** ^**2**^_**OLS**_	0.298	0.470	0.256	0.103	0.307	0.335	0.316	0.389	0.263	0.007	0.266
	**Coef**_**SAR**_	0.059^***^	0.083^***^	0.062^***^	-0.011	-0.119^***^	-0.045^***^	-0.066^***^	0.073^***^	0.060^***^	-0.008	0.040^***^
	***R*** ^**2**^_**SAR**_	0.480	0.546	0.513	0.440	0.595	0.696	0.502	0.511	0.436	0.426	0.498
**FD**	**Coef**_**OLS**_	0.090^***^	0.113^***^	0.080^***^	-0.055^***^	-0.084^***^	-0.055^***^	-0.090^***^	0.100^***^	0.083^***^	-0.007	0.092^***^
	***R*** ^**2**^_**OLS**_	0.256	0.400	0.199	0.097	0.224	0.095	0.257	0.316	0.215	0.002	0.267
	**Coef**_**SAR**_	0.044^***^	0.059^***^	0.044^***^	-0.008	-0.081^***^	-0.033^***^	-0.047^***^	0.051^***^	0.042^***^	-0.003	0.034^***^
	***R*** ^**2**^_**SAR**_	0.429	0.477	0.445	0.380	0.506	0.424	0.438	0.444	0.390	0.369	0.447
**NRI**	**Coef**_**OLS**_	-0.231^***^	-0.063	-0.250^***^	-0.248^***^	0.319^***^	0.168^***^	0.293^***^	-0.207^***^	-0.397^***^	0.254^***^	0.398^***^
	***R*** ^**2**^_**OLS**_	0.024	0.002	0.028	0.028	0.046	0.013	0.039	0.020	0.072	0.029	0.072
	**Coef**_**SAR**_	-0.346^***^	0.357^***^	-0.267^***^	-0.134^**^	0.449^***^	0.228^***^	0.296^***^	-0.143^**^	-0.336^***^	0.075^*^	0.178^**^
	***R*** ^**2**^_**SAR**_	0.271	0.285	0.290	0.258	0.332	0.272	0.295	0.279	0.279	0.272	0.284
**NFRI**	**Coef**_**OLS**_	-0.706^***^	-0.911^***^	-0.711^***^	0.426^***^	1.117^***^	0.494^***^	0.697^***^	-1.010^***^	-0.697^***^	0.312^***^	-0.732^***^
	***R*** ^**2**^_**OLS**_	0.143	0.238	0.145	0.052	0.358	0.070	0.139	0.293	0.139	0.028	0.154
	**Coef**_**SAR**_	-0.332^***^	-0.589^***^	-0.309^***^	0.139^*^	0.957^***^	0.206^**^	0.346^***^	-0.486^***^	-0.331^***^	0.140^**^	-0.362^***^
	***R*** ^**2**^_**SAR**_	0.313	0.394	0.348	0.337	0.471	0.325	0.349	0.367	0.300	0.309	0.366

**Fig. 3:**
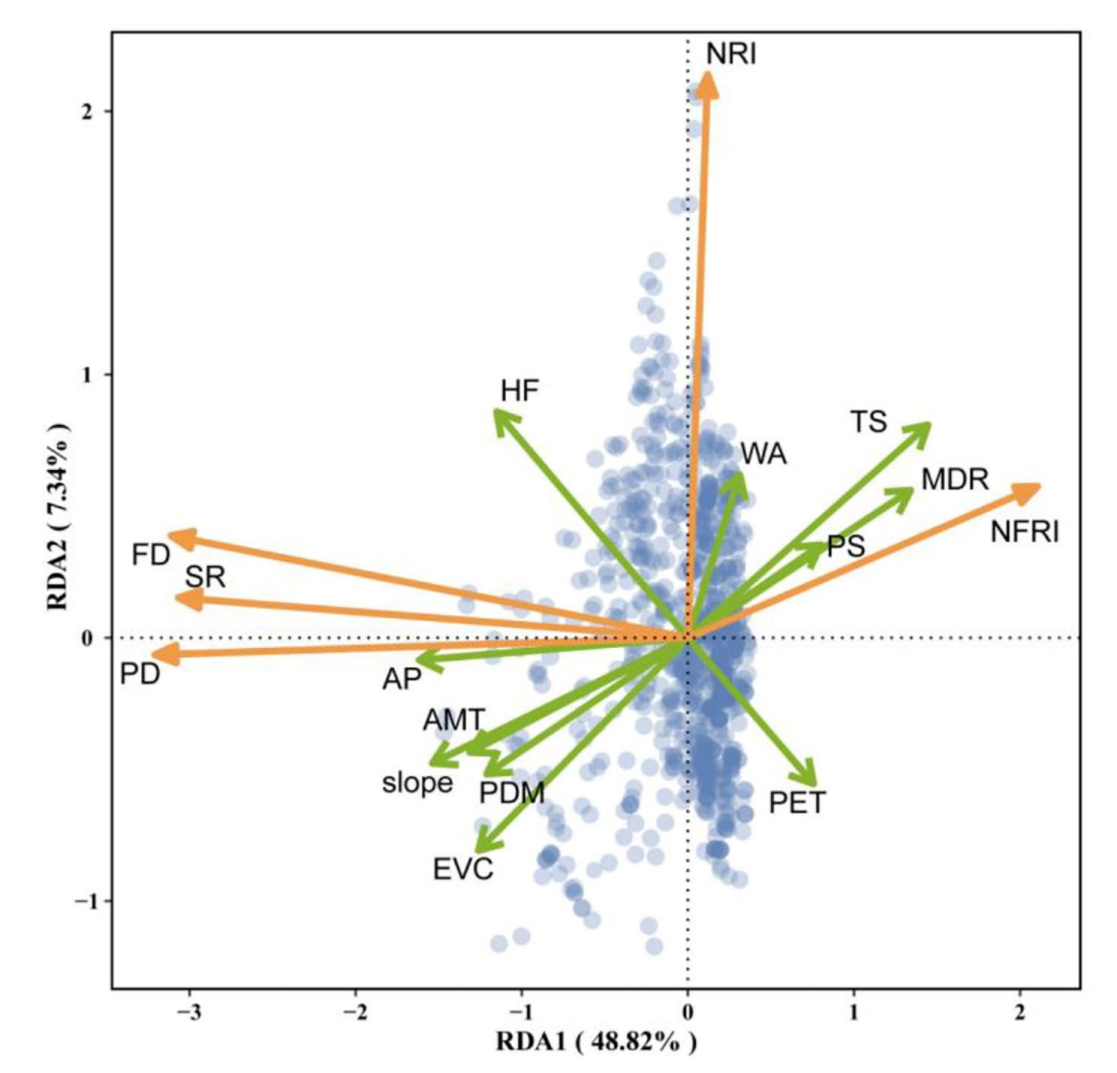
The species richness (SR), phylogenetic diversity (PD), functional diversity (FD), net relatedness index (NRI), net functional relatedness index (NFRI) and redundancy analysis between environmental variables of wetland plants were sorted. The energy-water variables are annual mean temperature (AMT), annual precipitation (AP), potential evapotranspiration (PET), Precipitation of driest month (PDM). The climate seasonality variables are mean diurnal range (MDR), temperature seasonality (TS) and precipitation seasonality (PS). The variable of human activities is human footprint (HF). The topographic variables are wetland area (WA), elevation variation coefficient (EVC) and slope (Slope). The brown arrows represent diversity indices and the green arrows represent the energy-water, climate seasonality, human activities and topography variables

**Fig. 4:**
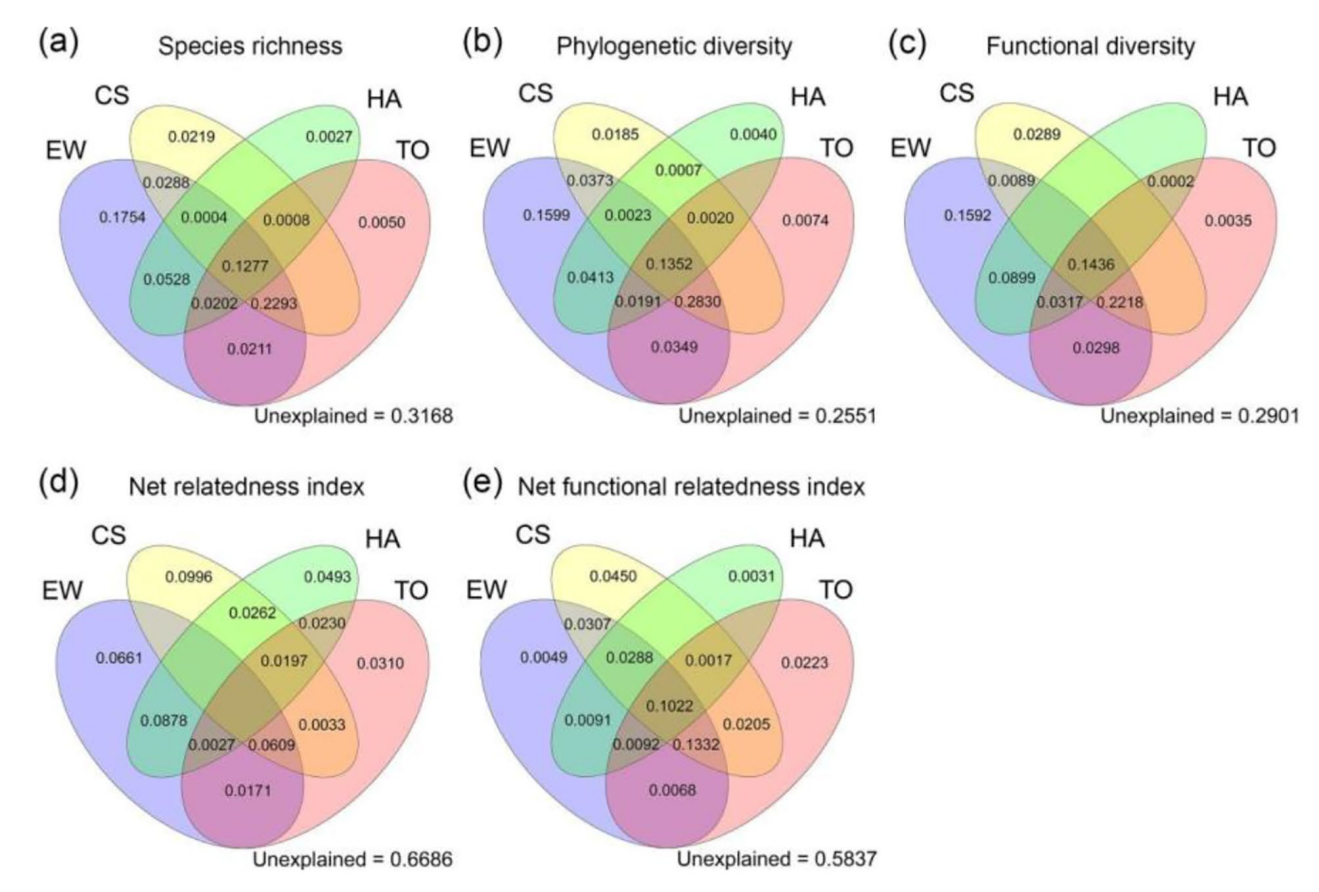
Variance partitioning (proportions) of species richness (SR) (**a**), phylogenetic diversity (PD) (**b**), functional diversity (FD) (**c**), net relatedness index (NRI) (**d**) and net functional relatedness index (NFRI) (**e**) for wetland plants into the independent effects of energy-water (EW), climate seasonality (CS), human activities (HA) and topography (TO). Negative values are not shown in the figures

The RDA analysis of wetland plant diversity and environmental indicators showed that the RDA results were statistically significant, and the first two axes accounted for 48.82% and 7.34% of the variance, respectively (Fig. 3). SR, PD and FD were positively correlated with annual mean temperature (AMT), annual precipitation (AP), slope (Slope), elevation variation coefficient (EVC), precipitation of driest month (PDM) and human footprint (HF), and negatively correlated with mean diurnal range (MDR), precipitation seasonality (PS), potential evapotranspiration (PET) and temperature seasonality (TS). NRI had a low correlation with most of the environmental variables. NFRI was positively correlated with temperature seasonality (TS), temperature seasonality (TS) and mean diurnal range (MDR), and negatively correlated with annual mean temperature (AMT), annual precipitation (AP), precipitation of driest month (PDM), elevation variation coefficient (EVC) and slope (Slope). In this study, In this study, we investigated the relative contribution of different categories of environmental variables (energy-water, climate seasonality, human activities and topography) to the taxonomic, phylogenetic and functional diversity of wetland plants. The results showed that the four categories of variables could explain the taxonomic diversity of wetland plants well, with all variables together explaining 68.32%, 74.49% and 70.99% of the variance in SR, PD and FD respectively (Fig. 4a, b,c). Among them, the variance explained by the energy-water and climate seasonality variables alone was also high, i.e. the energy-water environmental variable explains 65.57%, 71.30% and 68.49% of the variance in SR, PD and FD, and the climate seasonality variables explain 40.89%, 47.90% and 40.32% of the variance in SR, PD and FD. We found that the four categories of environmental variables can only explain 33.14% of the variance in NRI, indicating that environmental variables do not explain the phylogenetic structure of wetland plant communities well (Fig. 4d). On the contrary, four categories of environmental variables can explain 41.63% of the variance in NFRI. At the same time, energy-water and climate seasonality variables explained 32.49% and 36.89% of the variance in NFRI, respectively (Fig. 4e). This showed that the functional structure of wetland plant assemblages was more influenced by environmental variables, especially energy-water and climate seasonality variables, compared to the phylogenetic structure.

### Centers of endemic plant distribution and conservation status

**Fig. 5:**
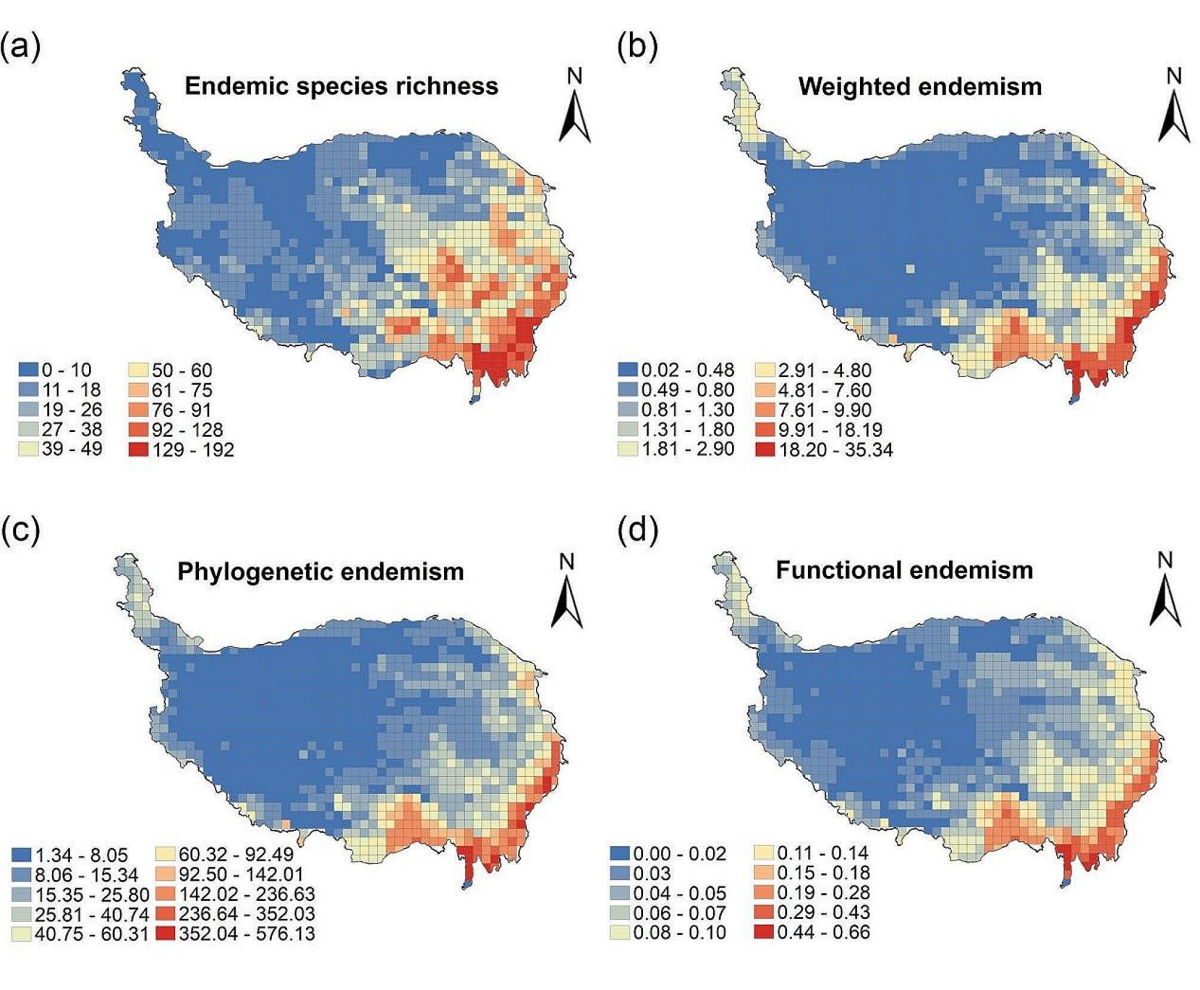
Geographic patterns of endemic centers of wetland plants in the Qinghai-Tibet Plateau. (**a**) endemic species richness, (**b**) weighted endemism, (**c**) phylogenetic endemism and (**d**) Functional endemism

**Fig. 6:**
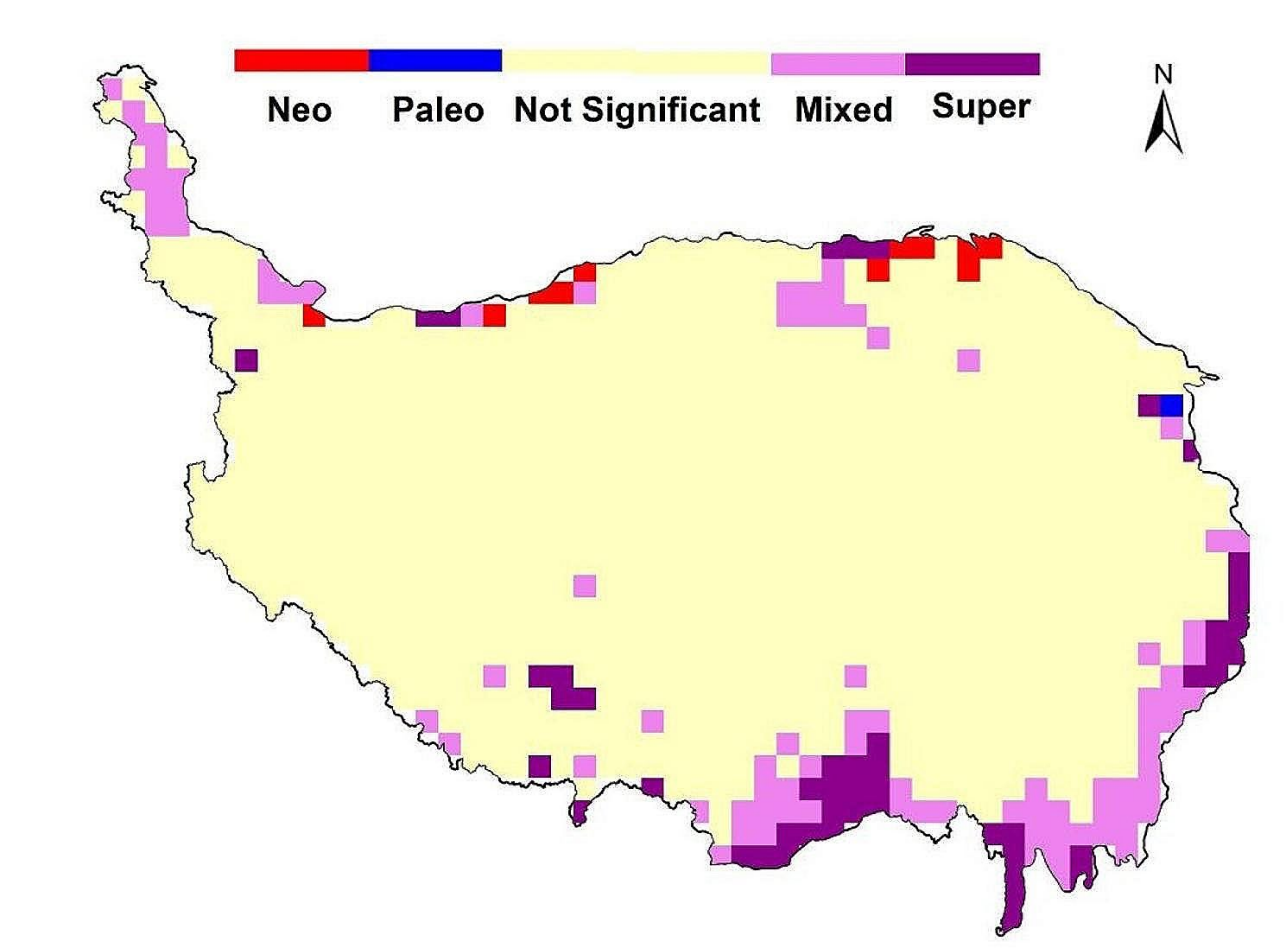
Centers of phylogenetic endemism inferred by CANAPE analysis. Red cells indicate centers of neo-endemism. Blue cells indicate centers of paleo-endemism. Purple cells indicate mixed endemism. Darker purple cells indicate super-endemism. Yellow cells are not significant

**Fig. 7:**
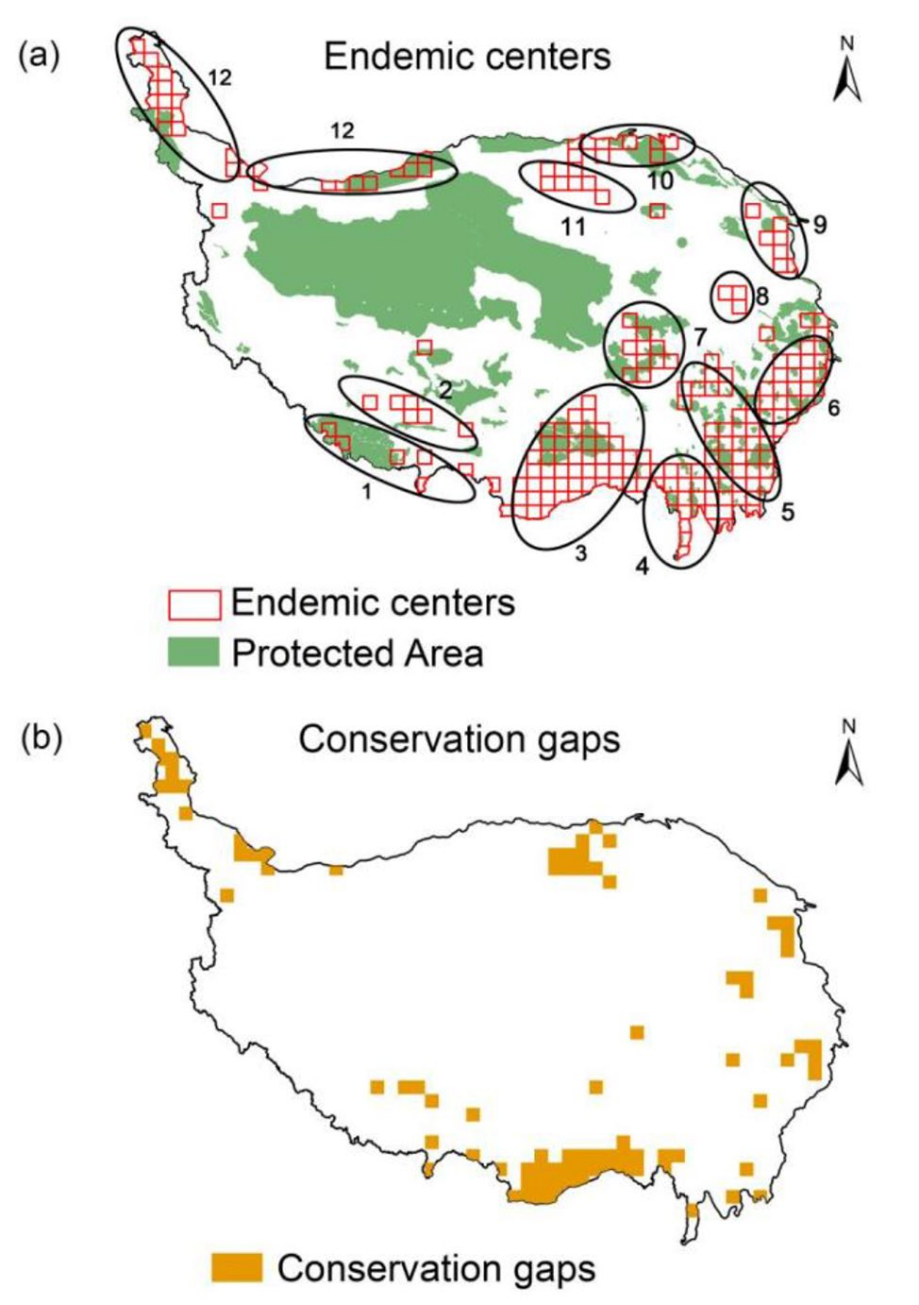
Hotspots of endemic wetland plant diversity in the Qinghai-Tibet Plateau (named after major mountains and rivers in the region) (**a**) and conservation gaps (**b**). 1: Central Himalayan region; 2: Gangdise Mountains; 3: Eastern Himalayas; 4: South-central part of Hengduan Mountains; 5: Daxueshan-Sharuli Mountains; 6: Minshan-Qionglai Mountains; 7: Three Rivers Source Region; 8: Anyemaqen Mountains; 9: Southeastern branch of Qilian Mountains; 10: Western part of Qilian Mountains-Altun Mountain Junction; 11: Qaidam Basin; 12: Pamir-Kunlun Mountains

The hotspots of endemic species richness of wetland plants in the QTP are mainly located in the Hengduan Mountains and the Eastern Himalayas in the southeast of the plateau, the Qilian Mountains in the northeast of the plateau, and the Three Rivers Source Region. The geographic distribution patterns of weighted endemism, phylogenetic endemism and functional endemism are similar to those of endemic species richness (Fig. 5a, b,c, d). The results of the CANAPE analysis indicate that the phylogenetic endemism of wetland plants in the QTP was dominated by mixed endemic and super-endemic types, with few purely neo-endemic and paleo-endemic types (Fig. 6). The marginal mountainous regions of the QTP, especially the Eastern Himalayas and the Hengduan Mountains, are continuous centers of mixed endemism and super-endemism, and these regions can serve as “museums” and “cradles” for the evolution of wetland plants. There are twelve hotspots of endemic wetland plant diversity in the QTP, mainly in the fringe mountains of the plateau, the Three Rivers Source Region and the Qaidam Basin (Fig. 7a). These include the Central Himalayas, the Gangdise Mountains, the Eastern Himalayas, the south-central part of the Hengduan Mountains, the Daxueshan-Sharuli Mountains, the Minshan-Qionglai Mountains, the Three Rivers Source Region, the Anyemaqen Mountains, the south-eastern branch of the Qilian Mountains, the western part of the Qilian Mountains-Altun Mountain Junction, the Qaidam Basin and the Pamir-Kunlun Mountains. The overlap between hotspots of endemic wetland plant diversity and nature reserves was analysed, and it was found that a small number of hotspots remain uncovered by protected areas. The main gaps in protection are in the Eastern Himalayas, Gangdise Mountains, Minshan-Qionglai Mountains, Anyemaqen Mountains (southeast), southeastern branch of Qilian Mountains, Qaidam Basin and Pamir-West Kunlun Mountains(Fig. 7b).

## Discussion

### Geographic patterns of species richness (SR), phylogenetic diversity (PD), functional diversity (FD) and their relationships

In this study, the SR and PD of wetland plants in the QTP showed a declining trend from the southeast to the northwest of the plateau, and the areas with higher SR and PD were mainly concentrated in the southeastern areas where the climate is relatively mild (e.g. Hengduan Mountains and Eastern Himalayas), while the areas with lower SR and PD were mainly concentrated in the western areas where the climate is harsh, which is consistent with the results of previous studies [34, 72]. Our results show that the SR of wetland plants decreases with increasing elevation, which is consistent with the elevational pattern of aquatic plants in the Himalayas of Nepal [73]. The decline in species diversity with increasing elevation is a widely reported trend [4]. Relatively harsh climatic conditions at high elevations, such as lower temperatures, greater climatic variability and lower habitat complexity, can lead to reduced SR. Meanwhile, stronger abiotic filtering limits the ability of species to colonise or persist in such environments [2]. We found that the elevational pattern of PD and FD was consistent with that of SR, and similar to the results for aquatic plants in the QTP and seed plants in Mount Kenya [4, 48]. In general, PD and FD metrics are commonly (but not always) closely correlated with SR [13]. The possible explanation for this is that species usually differ from each other with respect to their position in the phylogenetic tree [4, 74, 75] as well as due to their functional traits [76, 77].

### Geographic patterns of phylogenetic and functional structure and their relationships

We found that the geographic patterns of the NRI and NFRI are distinctly different, and they also show inconsistent patterns in terms of elevational gradients. In other words, the phylogenetic structure is not a good representation of the functional structure of wetland plants, which is consistent with the results of studies on the phylogenetic and functional structure of aquatic plants in the QTP [4]. The decoupling between phylogenetic and functional structure may be due to the fact that phylogenetic structure is largely influenced by historical processes, whereas functional structure is more related to contemporary and local environments [4, 9, 78]. It is generally accepted that the historical effects of high in situ speciation, niche conservatism and dispersal limitation may have led to phylogenetic clustering [34]. Uplift on the QTP facilitated in situ speciation, while climatic oscillations created harsh environments on the plateau that acted as dispersal barriers and filtered out these vulnerable species, meaning that rapid species formation and environmental filtering combined to produce phylogenetic clustering across much of the plateau [34]. The Himalayas in the southern plateau region have acted as one of the main historical corridors for species migration and dispersal [79], with large numbers of species moving from the Hengduan Mountains to recolonise the area [35], which may have resulted in a dispersed or random phylogenetic structure in the region. We found that in the warm and moist southeastern region, where environmental conditions are relatively stable, species with different functional traits coexist due to competitive exclusion, resulting in functional dispersion [13, 55]. In contrast, in the cold and dry northwestern region [35, 40], where environmental filtering dominates, only species with similar functional traits can coexist, resulting in functional clustering [13, 55].

### Influence of environmental variables on the geographic pattern of wetland plant diversity

The water-energy dynamic theory posits that temperature and precipitation jointly affect the available liquid water, thereby influencing biological activity in ecosystems, including productivity and the chemical energy required for species survival [20, 80]. Variance partitioning analysis shows that most of the variation in SR, PD and FD of wetland plants can be explained jointly by temperature and precipitation-related variables. This suggests that the geographic patterns of wetland plant diversity may be driven by the interaction of two types of climatic variables, i.e., energy and water. This is also indicated by the strong correlation of annual mean temperature (AMT) and annual precipitation (AP) with SR, PD and FD in the RDA analysis. Toivanen et al. [81] found that precipitation has a strong explanatory power for aquatic plant species richness in rivers and lakes across Finland. Studies show that precipitation is an important resource gradient, temperature is a regulating gradient [82], and thermal energy regulates the availability of liquid water through evapotranspiration [20]. Water availability is the primary determinant of wetland plant distribution as it strongly influences seed germination, establishment and plant growth [83], while temperature also influences plant physiological responses [4]. For example, low temperatures can cause sediments to freeze, which in turn limits light penetration and gas exchange between air and water, ultimately affecting the growth of wetland plants [84].

According to the theory of niche conservatism, limited climatic tolerance plays an important role in limiting species dispersal, which helps us to understand the phenomenon of higher species richness (SR) in areas with stable climate and lower SR in areas with more climate change and extreme events [85]. Precipitation seasonality is a measure of the tendency for a site or region to receive more precipitation in certain months or seasons than others (i.e., the higher the coefficient of variation, the greater the temporal fluctuations in precipitation). In areas with high seasonal precipitation, wetlands may dry out completely during the dry season, posing a significant challenge to wetland plant populations. In contrast, where there is less seasonal variation in precipitation, permanent wetlands are likely to retain moisture throughout the year and may support higher diversity [86]. Climate seasonality is the primary factor in determining richness variation in Moraceae [87]. Our results confirmed that climate seasonality is an important environmental variable influencing the geographic pattern of wetland plant diversity. The results of the RDA analysis showed that SR, PD and FD were strongly negatively correlated with climate seasonality variables such as temperature seasonality (TS), precipitation seasonality (PS) and mean diurnal range (MDR), which may be related to the physiological tolerance of plants to climate change.

Habitat heterogeneity has an important influence on plant diversity. Wetland plant diversity is influenced by a combination of habitat factors including slope, wetland area, and elevation variation coefficient. In this study, topographic variables explained 40.41% of the variance in SR and there was a strong positive correlation between Slope and SR, which is consistent with the hypothesis of habitat heterogeneity [18]. The QTP wetland environment is unique in that the topography controls the distribution and redistribution of water on slopes and in small watersheds, and dominates the flow production process on different slopes, which plays a critical role in wetland formation [33, 88]. Slope is an important topographic factor related to the effective water-holding capacity of soil moisture and plays an important role in wetland water regulation [89]. Therefore, we speculate that slope affects SR by mediating wetland formation. Interestingly, we did not find a significant correlation between SR and WA. Although the QTP has the largest number and area of lake complexes, some of them are brackish and saline lakes. For example, the Qiangtang Plateau in its northern part is dotted with over 900 lakes, of which 514 are brackish, saline and salt lakes combined, and only a few aquatic plants can grow under these conditions [4, 41].

Our results showed that environmental variables have a greater effect on the functional structure of wetland plants than on their phylogenetic structure, which may be related to the high plasticity of plant functional traits [9, 48]. This is consistent with the findings of the terrestrial flora of Inner Mongolia [78]. In addition, we found that among all the environmental variables, the climate seasonality variable had the most significant effect on the NFRI, explaining 36.89% of the variance in the NFRI. Previous studies have shown that climate seasonality is an important factor influencing the geographic variation in the structure of functional traits of woody plants in the Japanese archipelago, and that climate seasonality leads to convergence and divergence of co-occurring traits between different vegetation zones [90]. Goldel et al. [91] found that seasonality in temperature and precipitation played a major role in explaining geographic variation in leaf size, stem height and fruit size in New World palms (Arecaceae). Therefore, we speculate that climate seasonality may act as a filter for species traits, limiting the functional traits of species and leading to functional clustering [90], as indicated by the significant positive correlation between NFRI and climate seasonality variables temperature seasonality (TS), precipitation seasonality (PS) and mean diurnal range (MDR) in this study.

### Influence of human activities on the geographic pattern of wetland plant diversity

Contrary to expectations, human activities do not have a negative impact on wetland plant diversity. We found a strong colinearity between human activities variables and environmental variables (i.e., human footprint and climate are highly correlated), with variance partitioning showing a very low contribution of the human activities variables alone (the highest being the NRI at 0.0493). The possible explanation is that biodiversity tends to be higher in highly productive areas, where most energy and water resources are available. At the same time, population density is higher in areas where resources are more readily available and productivity is higher [3, 92–94]. For example, the wetlands of the QTP provide a wide variety of nutritious forage for livestock, and they are important in supporting local livestock farming [33]. Another reason may be that the human footprint used in this study is a composite variable that includes factors such as road traffic and population density [93, 95]. Therefore, sampling may be more complete in areas of high human activity. However, our study is based on large-scale patterns (i.e. coarse spatial resolution) and individual grid cells may show high levels of anthropogenic influence. Furthermore, within the large spatial extent, multiple refugia of wetland plants may exist within grid cells. Therefore, the results of this particular relationship represent a high probability of chance. We speculate that at finer spatial resolutions the correlation between wetland plant diversity and anthropogenic variables may weaken or become negatively correlated, which needs to be further studied [93].

### Conservation concerns for wetland plants

Our results showed that the Eastern Himalayas and the Hengduan Mountains are areas of high wetland plant endemism, which is consistent with previous studies [26, 29, 38, 58, 96]. The centers of wetland plant endemism in the QTP are mainly located in these mountainous regions of the Himalayas and the Hengduan Mountains, which have long been regarded as areas of high biodiversity and endemism, with many young endemics [26, 29, 96]. The high topographic heterogeneity of these areas, particularly in the mountainous uplifts, may have provided a stimulus for the formation of plant species, thereby promoting species diversity and a high degree of endemism [26]. At the same time, the natural barriers formed by the mountains may have restricted north-south and east-west migration and exchange of plants, creating a geographically isolated area that facilitated the formation and differentiation of endemism [26, 38]. Geological history may also have influenced the distribution of endemic wetland plants on the plateau, with the southeastern plateau providing refuge for species during the glacial period [34, 38]. Glaciation can influence species diversity by isolating habitats and limiting migration [15], which also contributes to wetland plant endemism. Studies have shown that many endemic species and remnant genera of vascular plants are found in these areas [26]. The results of the CANAPE analysis show that phylogenetic endemism of wetland plants in the QTP is dominated by mixed endemism and super-endemism, while pure neo-endemism and paleo-endemism are rare. Environmental stability is not only a prerequisite for the preservation of relict lineages, but also a driving factor for the emergence of new taxonomic groups. Glacial refugia are usually associated with variable terrain and provide such stability by buffering extreme climatic fluctuations. Therefore, the coexistence of neoendemic and paleoendemic phenomena occurs in many regions[97].

The study found that the endemic distribution centers of wetland plants in the QTP are mainly located in nature reserves, which is important for the conservation of endemic wetland plants and reflects the effectiveness of the establishment of national nature reserves. However, there are still some conservation gaps, especially in the Eastern Himalayas, Qaidam Basin and Pamir-West Kunlun Mountains. We suggest that more attention should be paid to the above areas in the future optimisation of protected areas. As most of the endemic plants in the QTP are alpine plants that are adapted to cold environments and can only survive at high elevations, they are vulnerable to the effects of climate warming [38]. Therefore, in our future work, we will focus on the effects of global climate change on endemic species of plateau wetland plants.

## Conclusion

In summary, based on the three dimensions of biodiversity (taxonomic diversity, phylogenetic diversity and functional diversity), this study comprehensively revealed the geographic pattern of wetland plant diversity in the QTP and its correlation with geographic and climatic variables. The geographic patterns of taxonomic, phylogenetic and functional diversity of wetland plants in the QTP are highly consistent, while the phylogenetic and functional structures show inconsistent geographic patterns. The geographic patterns of wetland plant diversity in the QTP are most influenced by energy-water and climate seasonality variables. The influence of environmental variables on functional structure is greater than that on phylogenetic structure. Phylogenetic endemism of wetland plants in the QTP was dominated by mixed endemic and super-endemic types, with few purely neo-endemic and paleo-endemic types. The conservation gaps for the distribution centers of endemic wetland plants in the QTP are mainly in the Eastern Himalayas, the Qaidam Basin and the Pamir-Western Kunlun Mountains. Our work provides insights into understanding the large-scale pattern of wetland plants in the QTP and is also an important addition to previous studies on the macro-ecological pattern of terrestrial organisms, as well as providing useful information for conservation planning.

### Electronic supplementary material

Below is the link to the electronic supplementary material.

Supplementary Material 1

Supplementary Material 2

Supplementary Material 3

Supplementary Material 4

Supplementary Material 5

Supplementary Material 6

## Data Availability

All data generated or analysed during this study are included in this published article [and its supplementary information files].

## Abbreviations

QTP: Qinghai-Tibet Plateau
SR: Species richness
PD: Phylogenetic diversity
FD: Functional diversity
NRI: Net relatedness index
NFRI: Net functional relatedness index
CANAPE: Categorical analysis of neo- and paleo-endemism
