# Supplementary material for: Large-scale geographic patterns and environmental and anthropogenic drivers of wetland plant diversity in the Qinghai-Tibet Plateau
Source: BMC Ecol Evol. 2024 Jun 3;24:74. doi: 10.1186/s12862-024-02263-w (PMC11145778; doi:10.1186/s12862-024-02263-w)
Supplement: Supplementary file 4 — Supplementary Material 4 [file 12862_2024_2263_MOESM4_ESM.pdf]

Additional file 4.

The Newick version of the phylogeny of wetland plants in the Qinghai-Tibet Plateau

((((((((((((((Sonchella_dentata:45.259634,Neobrachyactis_roylei:45.259634,Eschenbachia_muliensis:45.259634,(((((((((Adenostemma_lavenia:17.695347,Acmella_paniculata:17.695347):0.462661,(Bidens_maximowicziana:5.015196,Bidens_parviflora:5.015197,(Bidens_cernua:0.005555,Bidens_tripartita:0.005555):5.009642):13.142811):1.951761,Centipeda_minima:20.109769):0.793155,(((Anaphalis_aureopunctata:1.553864,Anaphalis_nepalensis:1.553865):0.229117,Pseudognaphalium_affine:1.782982):4.975269,((Leontopodium_stoloniferum:1.175402,Leontopodium_conglobatum:1.175402,Leontopodium_franchetii:1.175403,(((Leontopodium_ochroleucum:0.505166,Leontopodium_artemisiifolium:0.505166):0.001617,Leontopodium_souliei:0.506783):0.004812,Leontopodium_calocephalum:0.511595):0.663808):0.034173,(((Leontopodium_nanum:0.086597,Leontopodium_himalayanum:0.086597):0.010669,Leontopodium_pusillum:0.097266):0.007705,Leontopodium_stracheyi:0.104971):1.104605):5.548675):14.144673):1.196783,((Inula_racemosa:5.866189,((Inula_caspica:0.45305,Inula_japonica:0.45305):0.197382,Inula_britannica:0.650432):5.215758):0.328687,(Carpesium_minus:5.263094,Carpesium_cernuum:5.263095):0.931782):15.90483):0.889753,(((((Erigeron_moupinensis:0.904545,Erigeron_breviscapus:0.904546):3.953071,((Kalimeris_longipetiolata:2.20582,(Tripolium_pannonicum:2.204875,Heteropappus_gouldii:2.204874):0.000945):0.64795,(Aster_veitchianus:2.851508,Aster_trichoneurus:2.851508,Aster_stracheyi:2.851508,Aster_semiprostratus:2.851508,Aster_likiangensis:2.851508,Aster_albescens:2.851508,((((((Heteropappus_crenatifolius:0.492576,Aster_tongolensis:0.492576):0.110431,Aster_vestitus:0.603007):0.201684,(Aster_dolichopodus:0.601262,Aster_ageratoides:0.601262):0.203429):0.15559,Kalimeris_indica:0.960281):0.614041,Aster_tataricus:1.574322):0.997973,(Aster_poliothamnus:1.658796,Aster_falcifolius:1.658796):0.913499):0.279214,((Aster_diplostephioides:1.407556,(Aster_flaccidus:0.517475,Aster_asteroides:0.517475):0.890081):0.843748,((Aster_argyropholis:0.177195,Aster_lavandulifolius:0.177195):2.067806,Aster_fuscescens:2.245001):0.006303):0.600205):0.002261):2.003847):0.123888,(Grangea_maderaspatana:3.554039,(Dichrocephala_benthamii:0.538548,Dichrocephala_integrifolia:0.538548):3.015491):1.427466):13.961597,((((((Artemisia_kuschakewiczii:1.605142,Artemisia_phaeolepis:1.605142):0.184971,Artemisia_capillaris:1.790113):0.036046,(Artemisia_verbenacea:1.096136,Artemisia_velutina:1.096136,Artemisia_robusta:1.096136,Artemisia_przewalskii:1.096136,Artemisia_leucophylla:1.096136,Artemisia_hedinii:1.096136,Artemisia_fulgens:1.096136,Artemisia_codonocephala:1.096136,Artemisia_caruifolia:1.096136,Artemisia_calophylla:1.096136,Artemisia_abaensis:1.096136,((Artemisia_macrocephala:0.267917,Artemisia_minor:0.267917):0.719026,(Artemisia_mongolica:0.648448,((Artemisia_dubia:0.390858,Artemisia_tangutica:0.390858):0.002788,(Artemisia_vulgaris:0.311889,Artemisia_lactiflora:0.311889):0.081757):0.254802):0.338495):0.109194):0.730022):0.515037,Ajania_khartensis:2.341196):0.521766,Artemisiella_stracheyi:2.862962):2.857527,Achillea_acuminata:5.720489):13.222613):2.505677,(((((Senecio_yungningensis:0.958808,Senecio_wightii:0.958808,Senecio_graciliflorus:0.958808,Senecio_dubitabilis:0.958808,Senecio_arachnanthus:0.958808,Senecio_albopurpureus:0.958808):0.145716,Senecio_nemorensis:1.104525):4.315963,Senecio_scandens:5.420488):1.534842,Gynura_bicolor:6.955329):5.956504,(((((Sinosenecio_oldhamianus:2.465485,Tephroseris_stolonifera:2.465484):1.808687,(Parasenecio_pilgerianus:3.512826,Parasenecio_hastiformis:3.512827):0.761345):0.00415,(Ligularia_transversifolia:3.5398,Ligularia_tenuipes:3.5398,Li

gularia_rockiana:3.5398,Ligularia_retusa:3.5398,Ligularia_potaninii:3.5398,Ligularia_phyllocolea:3.5398,Ligularia_microcephala:3.5398,Ligularia_microcardia:3.5398,Ligularia_longifolia:3.5398,Ligularia_lidjiangensis:3.5398,Ligularia_ghatsukupa:3.5398,Ligularia_atkinsonii:3.5398,(((((((((Ligularia_sagitta:0.464265,Ligularia_stenocephala:0.464265):0.085644,Ligularia_intermedia:0.549909):0.00625,Ligularia_przewalskii:0.556159):0.178897,Ligularia_caloxantha:0.735056):0.241335,((Ligularia_lamarum:0.120369,Ligularia_subspicata:0.120369):0.566163,Ligularia_liatroides:0.686532):0.289859):0.436016,(Ligularia_fischeri:0.290702,Ligularia_veitchiana:0.290702):1.121705):0.308706,(((Ligularia_dictyoneura:1.606335,Ligularia_virgaurea:1.606335):0.005506,Ligularia_pleurocaulis:1.611841):0.104047,(Ligularia_cyathiceps:0.003755,Ligularia_latihastata:0.003755):1.712133):0.005225):0.218483,(((Ligularia_platyglossa:1.078639,Ligularia_vellerea:1.078639):0.005881,Ligularia_hookeri:1.08452):0.84783,Ligularia_oligonema:1.93235):0.007246):0.55514,(Ligularia_cymbulifera:1.487904,Ligularia_tongolensis:1.487904):1.006832):1.045065,(((((Ligularia_curvisquama:0.12772,Ligularia_duciformis:0.12772):0.187771,(Ligularia_konkalingensis:0.164636,Ligularia_purdomii:0.164636):0.150855):1.558091,Ligularia_franchetiana:1.873582):1.465432,((Ligularia_sibirica:1.769505,Ligularia_hodgsonii:1.769505):0.900442,Ligularia_japonica:2.669947):0.669067):0.193016,Ligularia_rumicifolia:3.53203):0.007771):0.738521):0.095044,(Cremanthodium_stenactinium:3.443147,Cremanthodium_smithianum:3.443147,Cremanthodium_pulchrum:3.443147,Cremanthodium_petiolatum:3.443147,Cremanthodium_glandulipilosum:3.443147,Cremanthodium_daochengense:3.443147,Cremanthodium_chungdienense:3.443147,Cremanthodium_calcicola:3.443147,Cremanthodium_angustifolium:3.443147,(((Cremanthodium_stenoglossum:2.242599,(Cremanthodium_brunneopilosum:1.796269,Cremanthodium_lineare:1.796269):0.44633):0.457735,Cremanthodium_discoideum:2.700334):0.004438,Cremanthodium_ellisii:2.704772):0.738376):0.930218):7.668761,((Petasites_tricholobus:4.02673,Petasites_japonicus:4.02673):1.450774,Tussilago_farfara:5.477504):6.564623):0.869707):8.536945):1.540681):7.684679,(((((((Crepis_rigescens:4.254201,Crepis_bodinieri:4.254201):1.696835,Crepis_multicaulis:5.951037):2.498613,(Pseudoyoungia_simulatrix:4.73633,Youngia_japonica:4.73633):3.71332):0.796877,(Syncalathium_roseum:8.704246,(Ixeris_chinensis:6.652005,(Askellia_flexuosa:3.3260025,Askellia_karelinii:3.3260025):3.3260025):2.052241):0.542281):1.335822,(Taraxacum_scariosum:9.434822,Taraxacum_maurocarpum:9.434822,Taraxacum_eriopodum:9.434822,Taraxacum_albiflos:9.434822,Taraxacum_mongolicum:9.434823):1.147526):1.958913,(Sonchus_wightianus:3.133128,Sonchus_palustris:3.133128):9.408133):0.355331,Lactuca_tatarica:12.896593):17.777546):3.606085,((Saussurea_ussuriensis:3.051764,Saussurea_thoroldii:3.051764,Saussurea_thomsonii:3.051764,Saussurea_runcinata:3.051764,Saussurea_pulchra:3.051764,Saussurea_populifolia:3.051764,Saussurea_ovatifolia:3.051764,Saussurea_nimborum:3.051764,Saussurea_laciniata:3.051764,Saussurea_iodostegia:3.051764,Saussurea_chingiana:3.051764,Saussurea_caudata:3.051764,Saussurea_acroura:3.051764,((((Saussurea_erubescens:1.006379,Saussurea_phaeantha:1.006379):0.065588,Saussurea_obvallata:1.071967):0.593826,((Saussurea_subulata:0.885658,Saussurea_katochaete:0.885658):0.527776,Saussurea_stella:1.413434):0.252359):0.273487,(((((Saussurea_salsa:0.432629,Saussurea_amara:0.432629):0.21071,Saussurea_parviflora:0.643339):0.023439,Saussurea_japonica:0.666778):0.001241,Saussurea_salicifolia:0.668019):0.690838,Saussurea_involucrata:1.358857):0.580423):1.112485):6.373334,((Cirsium_souliei:3.428857,Cirsium_shansiense:3.428857,Cirsium_eriophoroides:3.428857,Cirsium_arvense:3.428858):1.29959,(Carduus_crispus:1.897734,Carduus_acanthoides:1.897734):2.830714):4.696651):24.855125):4.014047,Adenocaulon_himalaicum:38.29427):6.965364):23.009715,((Nymphoides_indica:6.463658,Nymphoides_peltata:6.463658):32.794685,Menyanthes_trifoliata:39.258343):29.011007):14.933416,(Peracarpa_carnosa:45.590069,(Codonopsis_benthamii:19.811691,Cyananthus_hookeri:19.811691):25.778379,(Lobelia_zeylanica:12.797892,Lobelia_s

essilifolia:12.797892,Lobelia_nummularia:12.797892,Lobelia_davidii:12.797892,Lobelia_alsinoides:1
2.797892,(Lobelia_nicotianifolia:12.540959,Lobelia_chinensis:12.540959):0.256934):32.792177):37.6
12696)Asterales.rn.d8s.tre:10.527446,((((((((((((((((Pimpinella_smithii:0.171016,Pimpinella_brachyst
yla:0.171016):3.168493,Angelica_decursiva:3.339509):1.973922,(Angelica_grosseserrata:5.300218,C
nidium_monnieri:5.300218):0.013213):0.499752,Ligusticum_brachylobum:5.813183):4.003921,((Pleu
rospermum_rivulorum:7.180422,(Ligusticum_sikiangense:4.556064,(Ligusticum_pteridophyllum:3.32
3317,Ligusticum_jeholense:3.323317):1.232748):2.624357):1.530128,Cnidium_salinum:8.71055):1.10
6554):6.685216,(Carum_carvi:16.296178,(Pimpinella_silvatica:7.588248,Pimpinella_kingdon.wardii:7.
588248,(((((Pimpinella_coriacea:0.492263,Pimpinella_yunnanensis:0.492263):0.938184,Pimpinella_ca
ndolleana:1.430447):1.964586,Pimpinella_diversifolia:3.395033):0.200978,Pimpinella_fargesii:3.5960
11):1.177736,Pimpinella_chungdienensis:4.773747):2.814502):8.707929):0.206142):6.030024,(Anthri
scus_sylvestris:18.260917,(Pleurospermum_hookeri:3.654084,Ligusticum_tachiroei:3.654084):14.606
833):4.271427):1.071837,(Sium_frigidum:13.314548,(((((Oenanthe_javanica:0.011263,Oenanthe_line
aris:0.011263):2.116719,Oenanthe_thomsonii:2.127982):0.16246,Oenanthe_hookeri:2.290442):1.2002
29,Oenanthe_benghalensis:3.490671):6.269202,Cicuta_virosa:9.759873):3.554675):10.289633):2.200
146,(((((Pimpinella_henryi:3.977576,Tongoloa_tenuifolia:3.977576):0.658498,Tongoloa_silaifolia:4.6
36074):4.361537,Tongoloa_elata:8.997611):1.518069,Pimpinella_rhomboidea:10.51568):7.512979,(C
yclorhiza_waltonii:11.357691,Pleurospermum_angelicoides:11.357691):6.670968):7.775668):2.35512
6,((Physospermopsis_kingdon.wardii:5.397517,Pleurospermum_nanum:5.397518):5.070477,(Pleurosp
ermum_aromaticum:5.330224,(Pleurospermum_franchetianum:3.802547,Pleurospermum_linearilobu
m:3.802547):1.527678):5.13777):17.691458):3.31348,Bupleurum_gracillimum:31.472933):1.33036,(C
hamaesium_viridiflorum:13.861491,Chamaesium_novemjugum:13.861491,Chamaesium_paradoxum:1
3.861492):18.941801):10.66454,(((Sanicula_hacquetioides:8.671508,Sanicula_caerulescens:8.671508):
0.431715,((Sanicula_astrantiifolia:6.983555,(Sanicula_orthacantha:3.212047,Sanicula_lamelligera:3.2
12047):3.771508):1.622769,Sanicula_elata:8.606324):0.4969):0.421612,Sanicula_chinensis:9.524836):
33.942997):6.45715,Dickinsia_hydrocotyloides:49.924983):6.052178,Centella_asiatica:55.977161):4.2
11005,(Hydrocotyle_wilfordii:12.804033,Hydrocotyle_salwinica:12.804033,Hydrocotyle_hookeri:12.8
04033,Hydrocotyle_himalaica:12.804033,(Hydrocotyle_javanica:9.303653,Hydrocotyle_sibthorpioides:
9.303653):3.500381):47.384132):25.452489,((((Valeriana_officinalis:9.93615,Valeriana_flaccidissima:
9.93615):7.603453,(Valeriana_hardwickii:11.614204,Valeriana_jatamansi:11.614204):5.925399):19.23
5106,(Dipsacus_asper:31.612686,Triplostegia_glandulifera:31.612686):5.162023):34.163256,(Sambuc
us_javanica:7.618099,Sambucus_adnata:7.618099):63.319866)Dipsacales.rn.d8s.tre:14.70269)mrcaott
1673ott2128:8.089557)mrcaott320ott1673:13.011199,(((((((((Schnabelia_terniflora:34.280417,Amethy
stea_caerulea:34.280417,(((((((Clinopodium_repens:4.179118,Clinopodium_gracile:4.179119):1.73614
9,(Mentha_asiatica:5.067829,Mentha_canadensis:5.06783):0.847438):9.9176,Prunella_vulgaris:15.832
868):0.673667,Glechoma_longituba:16.506535):0.643844,((Lycopus_europaeus:0.505162,Lycopus_ca
valeriei:0.505162):3.805073,Lycopus_lucidus:4.310235):12.840144):2.449966,Salvia_plebeia:19.6003
45):5.757407,((Elsholtzia_luteola:16.187179,(Elsholtzia_pilosa:15.921731,Elsholtzia_densa:15.92173
1):0.265449):0.333005,Mosla_dianthera:16.520185):8.837567):8.922666,(((Teucrium_viscidum:7.091
961,Teucrium_pilosum:7.091961):13.951387,(Ajuga_nipponensis:3.922368,Ajuga_forrestii:3.922368,
Ajuga_ciliata:3.922368):17.12098):7.532365,((((Lamium_amplexicaule:12.761009,(Phlomoides_tibeti
ca:4.399019,Phlomoides_dentosa:4.399019,Phlomoides_tuberosa:4.39902,Phlomoides_atropurpurea:4.
39902):8.361989):1.527775,(Stachys_oblongifolia:10.661834,Stachys_kouyangensis:10.661834,Stach
ys_affinis:10.661834):3.626949):4.213148,(Pogostemon_linearis:6.01903,Pogostemon_auricularius:6.

01903):12.482902):8.317217,(Scutellaria_yunnanensis:7.452624,Scutellaria_tenax:7.452624,Scutellari
a_discolor:7.452624,Scutellaria_barbata:7.452625):19.366524):1.756565):5.704704):6.008518,(((((Pe
dicularis_souliei:14.947576,Pedicularis_reptans:14.947576,Pedicularis_pseudoingens:14.947576,Pedic
ularis_pseudocurvituba:14.947576,Pedicularis_pantlingii:14.947576,Pedicularis_longistipitata:14.9475
76,Pedicularis_longipetiolata:14.947576,Pedicularis_latirostris:14.947576,Pedicularis_kialensis:14.947
576,Pedicularis_habachanensis:14.947576,Pedicularis_furfuracea:14.947576,Pedicularis_elliotii:14.94
7576,Pedicularis_deqinensis:14.947576,Pedicularis_aschistorrhyncha:14.947576,(((((((((((((((((Pedicu
laris_sphaerantha:5.319154,Pedicularis_gyrorhyncha:5.319154):0.586052,Pedicularis_densispica:5.90
5206):0.002295,Pedicularis_glabrescens:5.907501):0.410975,Pedicularis_chenocephala:6.318476):0.3
66486,Pedicularis_pygmaea:6.684962):0.341025,Pedicularis_lyrata:7.025987):0.210175,(Pedicularis_
diffusa:3.313916,Pedicularis_kansuensis:3.313916):3.922246):0.407289,Pedicularis_pseudomelampyri
flora:7.643451):0.363361,Pedicularis_microchila:8.006812):1.514235,(((((Pedicularis_plicata:0.67492
1,Pedicularis_cheilanthifolia:0.674921):0.004438,Pedicularis_globifera:0.679359):0.78105,Pedicularis
_roylei:1.460409):1.71671,Pedicularis_anas:3.177119):4.316063,Pedicularis_pheulpinii:7.493182):2.0
27865):0.013467,Pedicularis_rhynchotricha:9.534514):0.13239,((Pedicularis_szetschuanica:7.975878,
Pedicularis_spicata:7.975878):0.849855,(Pedicularis_verticillata:8.42482,Pedicularis_oliveriana:8.424
82):0.400913):0.841171):1.393649,(Pedicularis_superba:6.710089,Pedicularis_przewalskii:6.710089):
4.350464):0.115297,Pedicularis_resupinata:11.17585):0.062367,(((Pedicularis_macrosiphon:3.911576,
Pedicularis_muscicola:3.911576):2.422734,Pedicularis_vagans:6.33431):1.628927,Pedicularis_rhinant
hoides:7.963237):3.27498):0.765872,((Pedicularis_oederi:11.58944,(Pedicularis_flexuosa:11.061646,P
edicularis_megalantha:11.061646):0.527794):0.410963,Pedicularis_yui:12.000403):0.003686):0.50384
9,((((((Pedicularis_gruina:4.77051,(Pedicularis_dolichantha:4.410214,Pedicularis_variegata:4.410214):
0.360296):0.00369,Pedicularis_siphonantha:4.7742):2.583973,(((Pedicularis_davidii:5.345101,Pedicul
aris_oxycarpa:5.345101):1.517622,Pedicularis_longiflora:6.862723):0.492898,(Pedicularis_cranolopha:
5.634841,Pedicularis_chinensis:5.634841):1.72078):0.002552):0.002283,Pedicularis_strobilacea:7.360
456):2.631244,Pedicularis_axillaris:9.9917):2.029587,Pedicularis_cryptantha:12.021287):0.486651):0.
160746,(Pedicularis_lasiophrys:8.037584,Pedicularis_muscoides:8.037584):4.6311):2.278893,Pedicula
ris_vialii:14.947577):15.080731,Euphrasia_regelii:30.028308):3.893561,Lindenbergia_muraria:33.921
868):2.566144,(Microcarpaea_minima:33.240551,(Mimulus_tenellus:11.209258,Mimulus_bracteosus:
11.209258,Mimulus_bodinieri:11.209258,Mimulus_szechuanensis:11.209259):22.031293):3.247461):0.
8879,((Mazus_lecomtei:9.672871,Mazus_humilis:9.672871,Mazus_celsioides:9.672871,Mazus_pumil
us:9.672872):7.778204,Lancea_tibetica:17.451076):19.924837):2.913023):6.982502,(((Strobilanthes_i
nflata:32.419985,Asystasiella_neesiana:32.419986):11.000864,((Utricularia_scandens:29.853655,Utric
ularia_salwinensis:29.853655,((((((Utricularia_australis:1.609383,Utricularia_minor:1.609383):0.8358
85,Utricularia_vulgaris:2.445268):2.637907,Utricularia_intermedia:5.083175):7.157369,Utricularia_au
rea:12.240544):4.459333,Utricularia_gibba:16.699877):12.98691,Utricularia_striatula:29.686787):0.16
6869):3.033082,Pinguicula_alpina:32.886738):10.534112):3.743164,Verbena_officinalis:47.164014):0.
107424):1.129444,((Lindernia_pusilla:20.141143,Lindernia_procumbens:20.141143,Lindernia_nummu
lariifolia:20.141143,Lindernia_micrantha:20.141143,Lindernia_hyssopoides:20.141143,Lindernia_crus
tacea:20.141143,Lindernia_ciliata:20.141143,Lindernia_anagallis:20.141143,Lindernia_antipoda:20.14
1143):20.141143,Torenia_violacea:40.282286):8.118595):0.472529,((Limosella_aquatica:26.96054,(Sc
rophularia_incisa:5.472212,Scrophularia_elatior:5.472212):21.488327):1.834695,(Buddleja_lindleyana:
5.198864,Buddleja_officinalis:5.198864):23.596371):20.078176):4.059351,(Callitriche_palustris:51.16
0266,((((((Veronica_laxa:13.253477,Veronica_chayuensis:13.253477,Veronica_anagallis.aquatica:13.2

53477):3.571586,Veronica_javanica:16.825064):1.407358,(((((Veronica_anagalloides:0.077049,Veroni
ca_undulata:0.077049):0.755272,Veronica_oxycarpa:0.832321):3.865843,Veronica_beccabunga:4.698
164):5.389251,(Veronica_pusilla:5.818976,Veronica_serpyllifolia:5.818976):4.268439):7.716197,Pseu
dolysimachion_linariifolium:17.803612):0.42881):4.777076,Lagotis_brachystachya:23.009498):7.523
889,(Plantago_minuta:16.608584,Plantago_depressa:16.608584,(Plantago_major:3.078473,Plantago_a
siatica:3.078473):13.530112):13.924802):8.062321,Hippuris_vulgaris:38.595708):12.564559,(Dopatri
um_junceum:24.59673,Limnophila_sessiliflora:24.59673):26.563537):1.772495):5.166068,(Henckelia
_forrestii:18.091576,(Lysionotus_serratus:17.711733,Henckelia_pumila:17.711734):0.379843):40.007
253):30.148947,(((Microula_leiocarpa:5.955295,((((Microula_trichocarpa:4.135102,Microula_diffusa:
4.135102):0.294088,Microula_tibetica:4.42919):0.392488,Microula_younghusbandii:4.821678):0.192
427,Microula_sikkimensis:5.014105):0.941191,(((Microula_floribunda:2.758312,Microula_stenophyll
a:2.758312):2.057988,Actinocarya_tibetica:4.8163):0.779119,Metaeritrichium_microuloides:5.595419)
:0.359877):11.818028,(Myosotis_caespitosa:15.578242,(Trigonotis_rockii:3.327442,Trigonotis_omeie
nsis:3.327442,Trigonotis_heliotropifolia:3.327442,Trigonotis_gracilipes:3.327442,Trigonotis_cavalerie
i:3.327442,Trigonotis_peduncularis:3.327443):12.2508):2.195081):0.851271,(Hackelia_difformis:10.5
18661,(Eritrichium_tangkulaense:9.937988,Eritrichium_sessilifructum:9.937988,Eritrichium_pseudola
tifolium:9.937988,Eritrichium_fruticulosum:9.937988,Eritrichium_acicularum:9.937988):0.580673):8.
105933):69.623182)mrcaott248ott2108:1.503291,(((Galium_yunnanense:6.40221,Galium_spurium:6.4
0221,Galium_paradoxum:6.40221,Galium_karataviense:6.40221,Galium_innocuum:6.40221,Galium_
hoffmeisteri:6.40221,Galium_exile:6.40221,Galium_elegans:6.40221,Galium_dahuricum:6.40221,Gali
um_bungei:6.40221,Galium_asperuloides:6.40221,Galium_asperifolium:6.40221,Galium_boreale:6.40
2211,(Galium_verum:2.319261,Galium_tricornutum:2.319261):4.08295):15.735641,(Oldenlandia_cor
ymbosa:12.521085,((Oldenlandia_diffusa:8.02752,(Oldenlandia_herbacea:4.416733,Oldenlandia_verti
cillata:4.416733):3.610787):3.826863,(Neanotis_hirsuta:0.442685,Neanotis_wightiana:0.442685):11.4
11698):0.666702):9.616767):45.590011,((Vincetoxicum_forrestii:21.894458,Apocynum_pictum:21.89
4459):30.178658,((Comastoma_tenellum:13.979497,Comastoma_stellariifolium:13.979497,Comastom
a_pulmonarium:13.979497,Comastoma_polycladum:13.979497,Comastoma_pedunculatum:13.979497,
Comastoma_disepalum:13.979497,Comastoma_falcatum:13.979497):13.979497,(((((Gentianella_turke
stanorum:12.953998,((Swertia_wolfgangiana:9.807248,Swertia_wardii:9.807248,Swertia_tibetica:9.80
7248,Swertia_tetraptera:9.807248,Swertia_punicea:9.807248,Swertia_przewalskii:9.807248,Swertia_
mussotii:9.807248,Swertia_kouitchensis:9.807248,Swertia_hispidicalyx:9.807248,Swertia_graciliflora:
9.807248,Swertia_franchetiana:9.807248,Swertia_erythrosticta:9.807248,Swertia_diluta:9.807248,Swe
rtia_dichotoma:9.807248,Swertia_davidii:9.807248,Swertia_cincta:9.807248,Swertia_bifolia:9.807248,
((Swertia_bimaculata:5.113493,Swertia_macrosperma:5.113493):3.295401,Swertia_ciliata:8.408894):1.
398355,Swertia_nervosa:9.807249):3.077264,((Lomatogonium_rotatum:3.64037,Lomatogoniopsis_gal
eiformis:3.64037):5.750894,(Lomatogonium_macranthum:4.695631,Lomatogonium_longifolium:4.69
5631,Lomatogonium_gamosepalum:4.695631,Lomatogonium_forrestii:4.695631,Lomatogonium_brac
hyantherum:4.695632,Lomatogonium_carinthiacum:4.695632):4.695632):3.493249):0.069486):0.5904
8,Halenia_elliptica:13.544479):0.855014,(Gentianopsis_grandis:3.003786,Gentianopsis_barbata:3.003
786,Gentianopsis_paludosa:3.003787):11.395706):4.849087,(Gentiana_yokusai:10.491855,Gentiana_v
eitchiorum:10.491855,Gentiana_tricolor:10.491855,Gentiana_tatsienensis:10.491855,Gentiana_syringe
a:10.491855,Gentiana_sutchuenensis:10.491855,Gentiana_stylophora:10.491855,Gentiana_stipitata:10.
491855,Gentiana_siphonantha:10.491855,Gentiana_riparia:10.491855,Gentiana_qiujiangensis:10.4918
55,Gentiana_purdomii:10.491855,Gentiana_pudica:10.491855,Gentiana_pseudoaquatica:10.491855,G

entiana_picta:10.491855,Gentiana_piasezkii:10.491855,Gentiana_pedicellata:10.491855,Gentiana_pan
thaica:10.491855,Gentiana_officinalis:10.491855,Gentiana_nubigena:10.491855,Gentiana_ninglangen
sis:10.491855,Gentiana_mairei:10.491855,Gentiana_ludingensis:10.491855,Gentiana_leucomelaena:1
0.491855,Gentiana_helophila:10.491855,Gentiana_heleonastes:10.491855,Gentiana_grumii:10.491855,
Gentiana_grata:10.491855,Gentiana_flexicaulis:10.491855,Gentiana_exigua:10.491855,Gentiana_decu
mbens:10.491855,Gentiana_decorata:10.491855,Gentiana_dahurica:10.491855,Gentiana_crenulatotrun
cata:10.491855,Gentiana_crassuloides:10.491855,Gentiana_clarkei:10.491855,Gentiana_choanantha:1
0.491855,Gentiana_asparagoides:10.491855,Gentiana_aristata:10.491855,Gentiana_aquatica:10.49185
5,Gentiana_aperta:10.491855,Gentiana_amplicrater:10.491855,Gentiana_algida:10.491855,(Gentiana_
macrophylla:1.180125,Gentiana_straminea:1.180125):9.311731):8.756724):4.903324,Centaurium_pul
chellum:24.151904):3.807091):24.114122):15.654746)Gentianales.rn.d8s.tre:22.023205)mrcaott248ott
1191:16.990343)mrcaott248ott320:5.599318,((((Rhododendron_xiguense:5.244737,Rhododendron_we
bsterianum:5.244737,Rhododendron_viscidifolium:5.244737,Rhododendron_tubiforme:5.244737,Rho
dodendron_temenium:5.244737,Rhododendron_nitidulum:5.244737,Rhododendron_chamaethomsonii:
5.244737,Rhododendron_capitatum:5.244737,Rhododendron_galactinum:5.244738,((Rhododendron_h
ippophaeoides:0.162844,Rhododendron_nivale:0.162844):0.486962,Rhododendron_primuliflorum:0.6
49806):4.594932):21.604245,(Vaccinium_vitis.idaea:9.671681,Vaccinium_uliginosum:9.671682):17.1
77301):68.03431,(((((Primula_woodwardii:10.641988,Primula_waltonii:10.641988,Primula_walshii:1
0.641988,Primula_vialii:10.641988,Primula_vaginata:10.641988,Primula_tsariensis:10.641988,Primul
a_tangutica:10.641988,Primula_stenodonta:10.641988,Primula_stenocalyx:10.641988,Primula_silaens
is:10.641988,Primula_sapphirina:10.641988,Primula_russeola:10.641988,Primula_oxygraphidifolia:10.
641988,Primula_orbicularis:10.641988,Primula_optata:10.641988,Primula_monticola:10.641988,Prim
ula_macrophylla:10.641988,Primula_limbata:10.641988,Primula_laxiuscula:10.641988,Primula_lactuc
oides:10.641988,Primula_laciniata:10.641988,Primula_forbesii:10.641988,Primula_firmipes:10.64198
8,Primula_concinna:10.641988,Primula_calthifolia:10.641988,Primula_buryana:10.641988,Primula_b
athangensis:10.641988,(((Primula_algida:7.905159,(((Primula_deflexa:3.989269,((Primula_pseudoden
ticulata:2.741832,Primula_caldaria:2.741832):1.070458,Primula_denticulata:3.81229):0.176979):0.303
684,(Primula_pumilio:4.087819,Primula_capitata:4.087819):0.205134):2.288335,((((Primula_fascicula
ta:1.57705,Primula_tibetica:1.57705):0.008258,Primula_nutans:1.585308):2.139828,Primula_involucr
ata:3.725136):2.399971,(Primula_conspersa:3.33487,Primula_gemmifera:3.33487):2.790237):0.45618
1):1.323871):1.881325,((Primula_alpicola:2.770424,Primula_sikkimensis:2.770424):4.974695,Primula
_florindae:7.745119):2.041365):0.213695,Primula_souliei:10.000179):0.64181):15.0035,((((((((((Prim
ula_chrysochlora:0.816785,Primula_helodoxa:0.816785):1.098348,Primula_melanodonta:1.915133):0.
022085,Primula_prenantha:1.937218):0.014521,Primula_serratifolia:1.951739):0.620511,Primula_sec
undiflora:2.57225):1.394977,((Primula_wilsonii:1.435618,Primula_anisodora:1.435618):0.013064,Pri
mula_poissonii:1.448682):2.518545):0.398939,(((Primula_bulleyana:1.173549,Primula_beesiana:1.17
3549):0.141959,Primula_aurantiaca:1.315508):0.043933,Primula_chungensis:1.359441):3.006725):0.5
16547,Primula_amethystina:4.882713):0.325094,Primula_agleniana:5.207807):1.198552,(Primula_mo
upinensis:4.023268,Primula_calderiana:4.023268):2.383091):3.624035,Primula_nivalis:10.030394):15.
615095):1.219595,(((((Primula_polyneura:2.638552,Primula_septemloba:2.638552):2.26549,(Primula
_latisecta:2.150029,Primula_heucherifolia:2.150029):2.754013):1.140848,Primula_oreodoxa:6.04489):
1.32665,Primula_malacoides:7.37154):1.215199,Primula_obconica:8.586739):18.278345):5.348348,(
Androsace_tanggulashanensis:26.147877,Androsace_gmelinii:26.147877,(Androsace_zambalensis:0.4
96482,Androsace_yargongensis:0.496482):25.651396):6.065554):8.392698,(Lysimachia_tsarongensis:

21.33634,Lysimachia_pumila:21.33634,Lysimachia_platypetala:21.33634,Lysimachia_parvifolia:21.33
634,Lysimachia_foenum.graecum:21.33634,Lysimachia_drymarifolia:21.33634,Lysimachia_biflora:21.
33634,Lysimachia_barystachys:21.33634,((((((Lysimachia_congestiflora:2.604341,((Lysimachia_steno
sepala:2.158734,Lysimachia_rubiginosa:2.158734):0.006083,Lysimachia_phyllocephala:2.164817):0.4
39524):0.020945,(Lysimachia_omeiensis:1.68443,Lysimachia_hemsleyi:1.68443):0.940856):0.686837,
Lysimachia_hemsleyana:3.312123):2.531819,Lysimachia_paridiformis:5.843942):9.468233,(Lysimach
ia_candida:8.971705,(Lysimachia_lobelioides:7.915769,(Lysimachia_clethroides:1.595732,Lysimachia
_fortunei:1.595732):6.320037):1.055936):6.34047):1.646209,Lysimachia_maritima:16.958384):4.3779
57,(Lysimachia_microcarpa:1.408897,Lysimachia_capillipes:1.408897):19.927444):19.269789):54.277
163):8.693903,(Impatiens_xanthina:31.346521,Impatiens_vittata:31.346521,Impatiens_toxophora:31.3
46521,Impatiens_tortisepala:31.346521,Impatiens_thomsonii:31.346521,Impatiens_taronensis:31.3465
21,Impatiens_siculifer:31.346521,Impatiens_scutisepala:31.346521,Impatiens_ruiliensis:31.346521,Im
patiens_rostellata:31.346521,Impatiens_recurvicornis:31.346521,Impatiens_rectangula:31.346521,Imp
atiens_purpurea:31.346521,Impatiens_pseudokingii:31.346521,Impatiens_potaninii:31.346521,Impatie
ns_polyceras:31.346521,Impatiens_notolopha:31.346521,Impatiens_noli.tangere:31.346521,Impatiens
_mussotii:31.346521,Impatiens_muliensis:31.346521,Impatiens_mengtszeana:31.346521,Impatiens_m
embranifolia:31.346521,Impatiens_margaritifera:31.346521,Impatiens_lecomtei:31.346521,Impatiens_
latebracteata:31.346521,Impatiens_lasiophyton:31.346521,Impatiens_infirma:31.346521,Impatiens_ho
locentra:31.346521,Impatiens_gracilipes:31.346521,Impatiens_drepanophora:31.346521,Impatiens_di
varicata:31.346521,Impatiens_dicentra:31.346521,Impatiens_desmantha:31.346521,Impatiens_cyathifl
ora:31.346521,Impatiens_crassicaudex:31.346521,Impatiens_chungtienensis:31.346521,Impatiens_chi
nensis:31.346521,Impatiens_chimiliensis:31.346521,Impatiens_ceratophora:31.346521,Impatiens_brac
hycentra:31.346521,Impatiens_apsotis:31.346521,Impatiens_alpicola:31.346521,Impatiens_abbatis:31.
346521,((((Impatiens_gongshanensis:25.36373,Impatiens_rubrostriata:25.36373):3.040354,(Impatiens
_soulieana:19.592343,((Impatiens_barbata:9.550644,Impatiens_corchorifolia:9.550644):1.230176,(Im
patiens_delavayi:1.462581,Impatiens_nubigena:1.462581):9.318239):8.811523):8.811741):0.691659,I
mpatiens_arguta:29.095743):0.788716,(((((Impatiens_radiata:6.186344,Impatiens_racemosa:6.186344):
3.558897,(Impatiens_laxiflora:3.378039,Impatiens_tuberculata:3.378039):6.367202):1.118043,(((Impa
tiens_uliginosa:7.103227,Impatiens_bicornuta:7.103227):0.563536,Impatiens_aquatilis:7.666763):2.06
5569,(Impatiens_principis:8.953956,Impatiens_nyimana:8.953956):0.778376):1.130952):2.843204,Im
patiens_cymbifera:13.706488):1.929721,((Impatiens_sulcata:2.051536,Impatiens_fragicolor:2.051536):
10.409371,(Impatiens_falcifer:7.545755,Impatiens_scabrida:7.545755):4.915152):3.175302):14.24825)
:1.462063,(Impatiens_clavigera:3.77939,Impatiens_wilsonii:3.77939):27.567132):72.230674)Ericales.
rn.d8s.tre:8.763533)mrcaott248ott650:7.533505,((((((((Arenaria_zhongdianensis:32.806215,Arenaria_t
umengelaensis:32.806215,Arenaria_trichophora:32.806215,Arenaria_forrestii:32.806215,Arenaria_edg
eworthiana:32.806215,Arenaria_dsharaensis:32.806215,Arenaria_aksayqingensis:32.806215,(Arenaria
_neelgherrensis:0.489186,Arenaria_puranensis:0.489186):32.31703):0.252951,Arenaria_stracheyi:33.0
59167):12.303166,((Stellaria_uda:21.277595,Stellaria_palustris:21.277595,Stellaria_mainlingensis:21.
277595,(Cerastium_thomsonii:16.345153,(Stellaria_chinensis:10.688744,Stellaria_uliginosa:10.68874
4):5.65641):4.932442):3.259968,Pseudostellaria_tibetica:24.537563):20.824769):0.897757,Thylacospe
rmum_caespitosum:46.26009):5.434025,(Sagina_japonica:6.027835,Sagina_saginoides:6.027836):45.6
66279):0.735782,(Spergularia_diandra:12.516412,Spergularia_marina:12.516413):39.913484):17.6827
35,(((Salsola_monoptera:31.71325,((Suaeda_salsa:20.484426,Suaeda_przewalskii:20.484426,((Suaeda
_paradoxa:13.076303,Suaeda_glauca:13.076303):4.114319,(Suaeda_corniculata:2.963097,((Suaeda_he

terophylla:0.290426,Suaeda_stellatiflora:0.290426):2.247696,Suaeda_maritima:2.538122):0.424975):1
4.227525):3.293805):2.138645,(Salicornia_europaea:11.414608,Kalidium_foliatum:11.414608):11.208
464):9.090179):8.536166,((Atriplex_laevis:8.180593,(Chenopodium_gracilispicum:1.083217,Chenopo
dium_album:1.083218):7.097376):15.792071,Dysphania_schraderiana:23.972665):16.276752):3.4119
09,Alternanthera_sessilis:43.661326):26.451306):36.938676,((((Rumex_ucranicus:22.243073,Rumex_
trisetifer:22.243073,Rumex_pseudonatronatus:22.243073,Rumex_patientia:22.243073,Rumex_obtusif
olius:22.243073,Rumex_nepalensis:22.243073,Rumex_longifolius:22.243073,Rumex_japonicus:22.24
3073,Rumex_gmelinii:22.243073,Rumex_dentatus:22.243073,Rumex_crispus:22.243073,Rumex_chal
epensis:22.243073,Rumex_aquaticus:22.243073,Rumex_acetosa:22.243073,Rumex_angulatus:22.2430
73):22.243073,((((Polygonum_wallichii:6.197441,Polygonum_umbrosum:6.197441,Polygonum_tibeti
cum:6.197441,Polygonum_taquetii:6.197441,Polygonum_suffultoides:6.197441,Polygonum_strindber
gii:6.197441,Polygonum_sparsipilosum:6.197441,Polygonum_sinomontanum:6.197441,Polygonum_si
biricum:6.197441,Polygonum_rigidum:6.197441,Polygonum_praetermissum:6.197441,Polygonum_pa
tulum:6.197441,Polygonum_paleaceum:6.197441,Polygonum_muricatum:6.197441,Polygonum_millet
ii:6.197441,Polygonum_macrophyllum:6.197441,Polygonum_kawagoeanum:6.197441,Polygonum_hu
mile:6.197441,Polygonum_forrestii:6.197441,Polygonum_fertile:6.197441,Polygonum_argyrocoleon:
6.197441,((Polygonum_aviculare:3.971846,Polygonum_cognatum:3.971846):0.002737,Polygonum_pl
ebeium:3.974583):2.222859):8.364801,(Fallopia_dentatoalata:4.803675,Fallopia_convolvulus:4.80367
5):9.758568):12.024693,(((Rheum_palmatum:2.448096,Rheum_alexandrae:2.448096):1.931617,Rheu
m_moorcroftianum:4.379713):13.462257,Oxyria_digyna:17.84197):8.744966):7.219854,((((((Persicari
a_wallichii:7.915883,Persicaria_strigosa:7.915883,Persicaria_filiformis:7.915883,Persicaria_campanul
ata:7.915883,((((Polygonum_jucundum:2.516262,(Persicaria_longiseta:1.709072,Polygonum_posumb
u:1.709072):0.80719):0.00293,Polygonum_japonicum:2.519192):3.240824,((Persicaria_hydropiper:2.6
8109,Persicaria_barbata:2.68109):0.180395,Polygonum_pubescens:2.861485):2.898531):1.649513,(Pe
rsicaria_maculosa:2.139697,Polygonum_delicatulum:2.139697):5.269832):0.506355,(Persicaria_lapat
hifolia:4.214378,Persicaria_amphibia:4.214378):3.701506):3.753698,Polygonum_viscosum:11.669582)
:6.381394,((((Polygonum_maackianum:5.725161,Polygonum_hastatosagittatum:5.725161):0.381242,P
olygonum_thunbergii:6.106403):2.677115,Polygonum_dissitiflorum:8.783518):3.80319,(Polygonum_s
enticosum:10.278738,(Polygonum_perfoliatum:6.199895,Persicaria_sagittata:6.199895):4.078843):2.3
0797):5.464268):5.944646,(((Persicaria_chinensis:8.910687,Persicaria_nepalensis:8.910687):1.378903,
(Polygonum_glaciale:6.489281,Polygonum_runcinatum:6.489281):3.800309):4.035897,Persicaria_cap
itata:14.325487):9.670135):6.421891,(((Polygonum_suffultum:21.744015,Persicaria_vivipara:21.7440
15):3.834786,Polygonum_filicaule:25.578801):0.011865,Koenigia_islandica:25.590666):4.826847):2.
88651,Fagopyrum_acutatum:33.304023):0.502767):10.679357):48.769402,(Tamarix_hispida:21.35791,
(Myricaria_paniculata:7.231232,(Myricaria_germanica:6.275369,((Myricaria_wardii:4.026836,Myrica
ria_rosea:4.026836):0.005205,Myricaria_prostrata:4.032041):2.243328):0.955864,Myricaria_elegans:7.
231233):14.126678):71.897638):6.039987,Drosera_peltata:99.295536):7.755772)Caryophyllales.rn.d8
s.tre:12.822926)mrcaott248ott557:3.860003,(((((((Phyllolobium_turgidocarpum:42.3816685,Phyllolob
ium_eutrichus:42.3816685,Phyllolobium_heydei:42.3816685):42.3816685,(((((Medicago_lupulina:18.
559242,(((Vicia_bungei:8.980356,Vicia_unijuga:8.980356):1.81156,Vicia_cracca:10.791916):0.56839
3,((Lathyrus_palustris:1.302937,Lathyrus_dielsianus:1.302937):6.912286,Lathyrus_pratensis:8.215223)
:3.145086):7.198933):13.700617,(((Astragalus_yangtzeanus:10.208616,Astragalus_webbianus:10.208
616,Astragalus_tibetanus:10.208616,Astragalus_sulcatus:10.208616,Astragalus_strictus:10.208616,Ast
ragalus_souliei:10.208616,Astragalus_rigidulus:10.208616,Astragalus_nivalis:10.208616,Astragalus_n

anjiangianus:10.208616,Astragalus_muliensis:10.208616,Astragalus_minutidentatus:10.208616,Astrag
alus_mahoschanicus:10.208616,Astragalus_longilobus:10.208616,Astragalus_lithophilus:10.208616,A
stragalus_licentianus:10.208616,Astragalus_leucocephalus:10.208616,Astragalus_leansanicus:10.2086
16,Astragalus_laxmannii:10.208616,Astragalus_laspurensis:10.208616,Astragalus_kuschakewiczii:10.
208616,Astragalus_hotianensis:10.208616,Astragalus_hoantchy:10.208616,Astragalus_handelii:10.20
8616,Astragalus_densiflorus:10.208616,Astragalus_dahuricus:10.208616,Astragalus_chilienshanensis:
10.208616,Astragalus_changduensis:10.208616,Astragalus_chagyabensis:10.208616,Astragalus_bhota
nensis:10.208616,Astragalus_arnoldii:10.208616):1.460318,((Oxytropis_falcata:0.700792,Oxytropis_
microphylla:0.700792):1.802601,(Oxytropis_kansuensis:0.59074,Oxytropis_ochrocephala:0.59074):1.
912653):9.165542):16.985349,(Hedysarum_tibeticum:12.159925,(Hedysarum_alpinum:7.045103,Hed
ysarum_tanguticum:7.045103):5.114823):16.494358):3.605575):17.166662,(Lotus_frondosus:10.1795
37,(Lotus_tenuis:1.036986,Lotus_corniculatus:1.036986):9.142552):39.246983):8.204971,(Glycine_so
ja:30.444154,((Desmodium_elegans:11.515822,Desmodium_heterocarpon:11.515823):6.724602,Kum
merowia_striata:18.240425):12.20373):27.187337):6.584434,(((Crotalaria_prostrata:9.294261,Crotalar
ia_ferruginea:9.294261,Crotalaria_sessiliflora:9.294262):34.608696,(Thermopsis_alpina:20.94176,The
rmopsis_inflata:20.94176):22.961198):15.304252,Aeschynomene_indica:59.20721):5.008716):20.547
412):11.311575,Polygala_fallax:96.074912):16.626283,((((((((((((Potentilla_virgata:58.52047,Potentil
la_supina:58.52047,Potentilla_simulatrix:58.52047,Potentilla_reptans:58.52047,Potentilla_plumosa:58.
52047,Potentilla_parvifolia:58.52047,Potentilla_multiceps:58.52047,Potentilla_lancinata:58.52047,Pot
entilla_hypargyrea:58.52047,Potentilla_gelida:58.52047,Potentilla_fragarioides:58.52047,Potentilla_de
lavayi:58.52047,Potentilla_angustiloba:58.52047,((((Potentilla_multifida:23.102289,Potentilla_potanin
ii:23.102289):28.495502,Potentilla_inclinata:51.597791):1.18978,(((Potentilla_griffithii:21.979107,Pot
entilla_multicaulis:21.979107):24.813656,(Potentilla_saundersiana:36.955479,Potentilla_discolor:36.9
55479):9.837284):3.545197,Potentilla_kleiniana:50.33796):2.449611):1.111133,(Potentilla_conferta:4
4.850405,Potentilla_sericea:44.850405):9.048299):4.621767):7.538418,Potentilla_freyniana:66.05888
9):2.605539,(Potentilla_flagellaris:63.019839,Potentilla_centigrana:63.019839):5.644589):2.463297,((
(Potentilla_leuconota:14.899458,Potentilla_stenophylla:14.899458):15.57363,Potentilla_anserina:30.4
73088):29.167607,Potentilla_glabra:59.640695):11.48703):0.015463,(Duchesnea_chrysantha:31.86893,
Duchesnea_indica:31.86893):39.274258):7.538571,((Comarum_salesovianum:53.483949,Sibbaldia_pa
rviflora:53.483949):0.497998,Sibbaldianthe_bifurca:53.981947):24.699812):2.674252,(((Sanguisorba_
alpina:47.555845,Sanguisorba_officinalis:47.555845):10.522557,Sanguisorba_filiformis:58.078402):1
5.241934,Agrimonia_pilosa:73.320336):8.035675):0.679169,Rosa_mairei:82.03518):2.967608,(Rubus
_wallichianus:84.30543,Rubus_tsangii:84.30543,Rubus_trijugus:84.30543,Rubus_thibetanus:84.30543,
Rubus_rubrisetulosus:84.30543,Rubus_pinnatisepalus:84.30543,Rubus_paniculatus:84.30543,Rubus_n
yalamensis:84.30543,Rubus_mesogaeus:84.30543,Rubus_malifolius:84.30543,Rubus_idaeopsis:84.30
543,Rubus_hunanensis:84.30543,Rubus_flosculosus:84.30543,Rubus_eustephanos:84.30543,Rubus_a
mabilis:84.30543,(((((Rubus_pectinellus:24.709447,Rubus_assamensis:24.709447):24.794923,Rubus_
pectinarioides:49.50437):5.316357,Rubus_pungens:54.820727):5.227164,Rubus_corchorifolius:60.047
891):0.277926,(Rubus_coreanus:35.599657,(Rubus_alexeterius:34.559373,Rubus_niveus:34.559373):
1.040284):24.72616):23.979614,Rubus_biflorus:84.305431):0.697357):0.475769,(Geum_japonicum:1
7.874766,Geum_aleppicum:17.874767):67.60379):9.082623,Sorbaria_kirilowii:94.56118):4.400616,((
((((Pilea_salwinensis:25.449849,Pilea_pauciflora:25.449849,Pilea_monilifera:25.449849,Pilea_martini:
25.449849,Pilea_hilliana:25.449849,Pilea_auricularis:25.449849,(Achudemia_japonica:23.757622,(((P
ilea_melastomoides:5.199541,Pilea_angulata:5.199541):5.921622,Pilea_anisophylla:11.121163):10.35

5719,Pilea_pumila:21.476882):2.28074):1.692228,((Pilea_pseudonotata:8.97431,Pilea_notata:8.97431)
:12.340625,Pilea_sinofasciata:21.314935):4.134915):11.265331,Lecanthus_peduncularis:36.715181):3.
091091,(((Elatostema_pseudoficoides:24.223034,Elatostema_platyphyllum:24.223034,Elatostema_obt
usum:24.223034,Elatostema_monandrum:24.223034,Elatostema_ficoides:24.223034,Elatostema_balan
sae:24.223034,Elatostema_parvum:24.223035):0.256875,Procris_crenata:24.47991):0.496895,Pellioni
a_heteroloba:24.976805):14.829467):2.401548,(((Urtica_laetevirens:13.641878,Urtica_atrichocaulis:1
3.641878):0.628763,Urtica_cannabina:14.270641):21.361669,(Laportea_cuspidata:33.351316,Laporte
a_bulbifera:33.351317):2.280993):6.57551):4.491285,((((Boehmeria_clidemioides:15.666724,Boehme
ria_macrophylla:15.666725):5.0221,Debregeasia_orientalis:20.688825):9.565752,Pouzolzia_zeylanica:
30.254576):11.545031,Maoutia_puya:41.799608):4.899497):38.794311,(Hippophae_tibetana:9.020905,
Hippophae_neurocarpa:9.020905):76.472511):13.46838)Rosales.rn.d8s.tre:12.186212,Actinostemma_t
enerum:111.148008)mrcaott371ott2511:1.553188)mrcaott371ott579:3.084369,(((((((Hypericum_wighti
anum:14.460606,((Hypericum_perforatum:6.240696,(Hypericum_reptans:4.43065,(Hypericum_elodeo
ides:2.41833,Hypericum_monanthemum:2.41833):2.01232):1.810046):5.964807,(((Hypericum_acmos
epalum:0.095642,Hypericum_lagarocladum:0.095642):0.002903,Hypericum_beanii:0.098545):5.7484
6,Hypericum_ascyron:5.847005):6.358498):2.255104):10.482436,(Hypericum_japonicum:3.463411,H
ypericum_gramineum:3.463411):21.479632):6.027458,Triadenum_breviflorum:30.970501):63.894778,
((Salix_yadongensis:7.342431,Salix_wilhelmsiana:7.342431,Salix_variegata:7.342431,Salix_tenuijulis:
7.342431,Salix_taoensis:7.342431,Salix_sphaeronymphe:7.342431,Salix_sikkimensis:7.342431,Salix_
shihtsuanensis:7.342431,Salix_serpyllum:7.342431,Salix_sclerophylloides:7.342431,Salix_saposhniko
vii:7.342431,Salix_rockii:7.342431,Salix_rhoophila:7.342431,Salix_resecta:7.342431,Salix_rehderian
a:7.342431,Salix_pseudowallichiana:7.342431,Salix_pseudospissa:7.342431,Salix_phanera:7.342431,
Salix_paratetradenia:7.342431,Salix_oritrepha:7.342431,Salix_myrtillacea:7.342431,Salix_michelsonii:
7.342431,Salix_luctuosa:7.342431,Salix_hypoleuca:7.342431,Salix_haoana:7.342431,Salix_fedtschen
koi:7.342431,Salix_etosia:7.342431,Salix_eriostachya:7.342431,Salix_erioclada:7.342431,Salix_driop
hila:7.342431,Salix_divergentistyla:7.342431,Salix_dissa:7.342431,Salix_disperma:7.342431,Salix_di
bapha:7.342431,Salix_denticulata:7.342431,Salix_delavayana:7.342431,Salix_daliensis:7.342431,Sali
x_cyanolimnea:7.342431,Salix_cheilophila:7.342431,Salix_characta:7.342431,Salix_caesia:7.342431,
Salix_brachista:7.342431,Salix_atopantha:7.342431,Salix_rosmarinifolia:7.342432):87.019623,(Viola_
urophylla:14.937105,Viola_thomsonii:14.937105,Viola_sikkimensis:14.937105,Viola_pendulicarpa:14.
937105,Viola_forrestiana:14.937105,Viola_confertifolia:14.937105,((((Viola_delavayi:1.355332,Viola
_biflora:1.355332):6.87179,(Viola_moupinensis:2.923278,Viola_arcuata:2.923278):5.303844):0.30994
3,((Viola_diffusa:5.280027,Viola_fargesii:5.280027):1.536842,Viola_grandisepala:6.816869):1.720196)
:2.672999,(((Viola_prionantha:2.883802,Viola_inconspicua:2.883802):1.233263,Viola_selkirkii:4.1170
65):0.059507,Viola_chaerophylloides:4.176572):7.033492):3.727042):79.424949):0.503224):7.74248,(
Homonoia_riparia:61.896506,Excoecaria_acerifolia:61.896506):40.711252):8.818668,(Oxalis_griffithi
i:22.8346595,Oxalis_corniculata:22.8346595):88.5917675)mrcaott2ott345:0.594159,(Parnassia_pusilla:
13.850866,Parnassia_oreophila:13.850866,Parnassia_gansuensis:13.850866,Parnassia_crassifolia:13.8
50866,Parnassia_cacuminum:13.850866,(((((Parnassia_wightiana:5.719222,((Parnassia_delavayi:0.016
483,Parnassia_brevistyla:0.016483):3.116015,Parnassia_yunnanensis:3.132498):2.586724):0.013825,P
arnassia_trinervis:5.733047):0.518913,(Parnassia_farreri:1.04326,Parnassia_chinensis:1.04326):5.2087)
:1.170896,Parnassia_noemiae:7.422856):4.248394,(Parnassia_palustris:11.658008,(Parnassia_nubicola:
10.409136,Parnassia_laxmannii:10.409136):1.248872):0.013242):2.179617):98.169719)mrcaott2ott14
79:3.764979)mrcaott2ott371:2.793039,(((Aphragmus_hobsonii:16.7568825,Aphragmus_oxycarpus:16.

7568825):16.7568825,((((((Eutrema_yunnanense:11.561929,Eutrema_himalaicum:11.561929,Eutrema
_fontanum:11.561929,Eutrema_deltoideum:11.561929):0.015505,Pegaeophyton_scapiflorum:11.57743
5):15.16835,Megacarpaea_delavayi:26.745785):0.188645,(Draba_parviflora:8.852807,Draba_mongoli
ca:8.852807,(((Draba_lichiangensis:2.981737,(Draba_glomerata:0.9652159997,Draba_oreades:0.9652
159997):2.016521):1.934471,((Draba_stenocarpa:1.641537,Draba_involucrata:1.641537):1.200745,(D
raba_yunnanensis:2.547381,Draba_eriopoda:2.547381):0.294901):2.073926):1.485557,Draba_nemoro
sa:6.401765):2.451043):18.081622):0.367842,(Braya_rosea:9.548403,(Dilophia_salsa:4.7687895,Dilo
phia_ebracteata:4.7687895):4.7796135):17.753869):0.146893,((((Arabidopsis_thaliana:13.11896,Catol
obus_pendulus:13.11896):9.89668,((Cardamine_trifoliolata:13.018777,Cardamine_simplex:13.018777,
Cardamine_rockii:13.018777,Cardamine_purpurascens:13.018777,Cardamine_pulchella:13.018777,Ca
rdamine_paucifolia:13.018777,Cardamine_multijuga:13.018777,Cardamine_multiflora:13.018777,Car
damine_lyrata:13.018777,Cardamine_loxostemonoides:13.018777,Cardamine_gracilis:13.018777,Card
amine_franchetiana:13.018777,Cardamine_calcicola:13.018777,(((((Cardamine_pratensis:5.777102,Car
damine_griffithii:5.777102):4.17502,(Cardamine_hirsuta:7.720233,Cardamine_parviflora:7.720233):2.
231889):0.854485,(Cardamine_circaeoides:3.400532,Cardamine_microzyga:3.400532):7.406075):0.25
986,(((Cardamine_macrophylla:0.9666739997,Cardamine_tangutorum:0.9666739997):9.482902,Carda
mine_yunnanensis:10.449576):0.020593,(Cardamine_leucantha:8.043547,Cardamine_impatiens:8.043
547):2.426622):0.596298):1.952311):3.766412,((Rorippa_globosa:9.909509,Rorippa_elata:9.909509,R
orippa_dubia:9.909509,((Rorippa_cantoniensis:1.093518,Rorippa_indica:1.093518):1.674884,Rorippa
_palustris:2.768402):7.141108):0.671336,Barbarea_intermedia:10.580845):6.204344):6.23045):0.4235
89,Lepidium_latifolium:23.439228):0.183,Smelowskia_tibetica:23.622229):3.826936):6.064601):83.4
7943,(Sarcopyramis_napalensis:93.319518,(((((Epilobium_williamsii:17.888129,Epilobium_wallichian
um:17.888129,Epilobium_subcoriaceum:17.888129,Epilobium_sinense:17.888129,Epilobium_sikkime
nse:17.888129,Epilobium_royleanum:17.888129,Epilobium_pyrricholophum:17.888129,Epilobium_pl
atystigmatosum:17.888129,Epilobium_pannosum:17.888129,Epilobium_minutiflorum:17.888129,Epil
obium_kingdonii:17.888129,Epilobium_kermodei:17.888129,Epilobium_brevifolium:17.888129,Epilo
bium_blinii:17.888129,Epilobium_amurense:17.888129,(((Epilobium_cylindricum:2.899684,Epilobiu
m_palustre:2.899684):0.110637,Epilobium_parviflorum:3.010321):0.615477,Epilobium_hirsutum:3.62
5798):14.262332,Epilobium_angustifolium:17.88813):24.183026,(Circaea_repens:8.318848,Circaea_a
lpina:8.318849):33.752307):18.493547,(Ludwigia_taiwanensis:18.867776,Ludwigia_prostrata:18.8677
76,Ludwigia_octovalvis:18.867776,Ludwigia_adscendens:18.867777):41.696926):12.071174,(((Trapa
_incisa:39.115177,(Ammannia_baccifera:9.65864,Ammannia_auriculata:9.65864):29.456536):0.99970
8,Lythrum_salicaria:40.114885):6.192002,(Rotala_indica:22.086074,Rotala_rotundifolia:22.086075):2
4.220812):26.32899):20.683641):23.673678)mrcaott96ott607:1.585408)mrcaott2ott96:3.826488,(((((Se
dum_tsinghaicum:24.45841874,Sedum_obtusipetalum:24.45841874,Sedum_fedtschenkoi:24.4584187
4,Sedum_chauveaudii:24.45841874,(Sedum_obtrullatum:14.67668132,Sedum_triactina:14.67668132):
9.78173842):13.09385856,((Rhodiola_sherriffii:5.5303795,(((Rhodiola_tangutica:2.39646824,Sedum_
quadrifidum:2.39646824):0.25812393,Rhodiola_tibetica:2.65459217):0.774672,(Rhodiola_himalensis:
3.1718143,Rhodiola_heterodonta:3.1718143):0.25744987):2.10111633,Rhodiola_alterna:5.5303805):1
8.736717,Phedimus_odontophyllus:24.2670975):13.2851808):56.0242427,(((Myriophyllum_spicatum:
10.9617757,Myriophyllum_verticillatum:10.9617757):9.9563797,Gonocarpus_micranthus:20.9181554)
:49.817553,Penthorum_chinense:70.7357084):22.8408126):11.106525,(((Saxifraga_tigrina:27.479794
09,Saxifraga_pardanthina:27.47979409,Saxifraga_oresbia:27.47979409,Saxifraga_nana:27.47979409,
Saxifraga_elliotii:27.47979409,Saxifraga_clavistaminea:27.47979409,(Saxifraga_cernua:23.84138069,

((((((((Saxifraga_heleonastes:0.1263535008,Saxifraga_parva:0.1263535008):0.671053908,Saxifraga_
ciliatopetala:0.7974074088):0.69981316,((Saxifraga_hookeri:1.426899665,Saxifraga_lychnitis:1.4268
99665):0.0685930522,Saxifraga_sinomontana:1.495492718):0.0017278513):0.038176587,Saxifraga_ti
betica:1.535397156):0.0022007783,Saxifraga_moorcroftiana:1.537597934):0.1146543709,(Saxifraga_
hirculus:0.6903797545,Saxifraga_maxionggouensis:0.6903797545):0.9618725505):0.21643963,Saxifr
aga_nigroglandulifera:1.868691935):2.708712554,Saxifraga_diapensia:4.577404489):2.5225589,(Saxi
fraga_gemmigera:4.547620696,Saxifraga_umbellulata:4.547620696):2.552342693):16.7414173):3.638
4144):11.59759441,Saxifraga_rufescens:39.0773895):8.523898,((((((Saxifraga_lumpuensis:1.1332850
44,Saxifraga_divaricata:1.133285044):0.29576185,Saxifraga_melanocentra:1.429046894):19.7159130
1,Saxifraga_pallida:21.1449599):11.9383695,(Chrysosplenium_lanuginosum:6.4364956,Chrysospleniu
m_davidianum:6.4364956):26.6468338):0.6839495,Tiarella_polyphylla:33.7672789):1.2496476,Astilb
e_rubra:35.0169265):12.584361):57.0817585):17.722046)mrcaott2ott2464:1.329145)Pentapetalae:4.8
1969,Pachysandra_axillaris:128.553927)mrcaott2ott8379:3.126773,((((((((((Ranunculus_yunnanensis:
13.278987,Ranunculus_trivedii:13.278987,Ranunculus_trautvetterianus:13.278987,Ranunculus_tangut
icus:13.278987,Ranunculus_popovii:13.278987,Ranunculus_minor:13.278987,Ranunculus_kamchatic
us:13.278987,Ranunculus_indivisus:13.278987,Ranunculus_furcatifidus:13.278987,Ranunculus_distan
s:13.278987,Ranunculus_dielsianus:13.278987,Ranunculus_banguoensis:13.278987,Ranunculus_alber
tii:13.278987,(((((((Ranunculus_cantoniensis:1.023868,Ranunculus_silerifolius:1.023868):0.247934,R
anunculus_trigonus:1.271802):0.564941,(Ranunculus_sinovaginatus:1.67264,Ranunculus_sieboldii:1.6
7264):0.164103):1.069727,Ranunculus_diffusus:2.90647):2.845127,Ranunculus_repens:5.751597):1.2
37417,Ranunculus_japonicus:6.989014):1.816146,Ranunculus_ficariifolius:8.80516):4.473828,(((((Ra
nunculus_potaninii:1.517773,Ranunculus_hirtellus:1.517773):0.308852,Ranunculus_brotherusii:1.826
625):0.123884,((Ranunculus_nematolobus:1.027395,Ranunculus_membranaceus:1.027395):0.253056,
Ranunculus_pseudopygmaeus:1.280451):0.670058):0.99962,((Ranunculus_pulchellus:0.740851,Ranun
culus_pedatifidus:0.740851):1.505715,Ranunculus_pegaeus:2.246566):0.703563):6.848754,(Batrachiu
m_bungei:5.335658,(Ranunculus_sceleratus:4.724085,Ranunculus_natans:4.724085):0.611573):4.463
225):3.480105):1.822499,(Batrachium_trichophyllum:12.384947,Batrachium_eradicatum:12.384947,B
atrachium_foeniculaceum:12.384948):2.716539):9.43623,(Oxygraphis_tenuifolia:12.876265,(Halerpes
tes_tricuspis:12.870562,Halerpestes_lancifolia:12.870562,Halerpestes_filisecta:12.870562,Halerpestes
_sarmentosa:12.870563,Halerpestes_ruthenica:12.870563):0.005702):11.661452):14.281139,((Clemati
s_pseudopogonandra:5.815276,(Clematis_tangutica:4.736985,Clematis_rehderiana:4.736985):1.07829
2):13.417144,(Anemone_rupestris:12.034303,(Anemone_rupicola:7.329027,Anemone_rivularis:7.329
027):4.705277,(Anemone_trullifolia:9.082713,(Anemone_flaccida:3.055893,Anemone_baicalensis:3.0
55893):6.02682):2.951591):7.198117):19.586435):2.472759,(((((Trollius_pumilus:0.443771,Trollius_r
anunculoides:0.443771):0.266447,Trollius_yunnanensis:0.710218):26.857739,(Caltha_palustris:5.2444
36,Caltha_scaposa:5.244436):22.323521):10.199461,Asteropyrum_cavaleriei:37.767418):1.917939,Ac
taea_asiatica:39.685357):1.606258):0.494432,(Aconitum_pseudobrunneum:23.184479,Delphinium_de
lavayi:23.18448,(Aconitum_tanguticum:5.476165,Aconitum_bulleyanum:5.476165):17.708315):18.60
1567):0.764671,(((Thalictrum_alpinum:3.687431,(Thalictrum_rutifolium:3.256049,Thalictrum_cultrat
um:3.256049):0.431382):0.649928,Thalictrum_squamiferum:4.337359):0.243236,(Thalictrum_simple
x:2.976722,Thalictrum_delavayi:2.976722):1.603873):37.970123):37.732339,(((Berberis_xanthophlae
a:2.678992,Berberis_ulicina:2.678992,Berberis_tenuipedicellata:2.678992,Berberis_racemulosa:2.678
992,Berberis_pseudotibetica:2.678992,Berberis_potaninii:2.678992,Berberis_obovatifolia:2.678992,Be
rberis_longispina:2.678992,Berberis_jiulongensis:2.678992,Berberis_jamesiana:2.678992,Berberis_ha

oi:2.678992,Berberis_feddeana:2.678992,Berberis_dielsiana:2.678992,Berberis_dictyophylla:2.678992,
Berberis_davidii:2.678992,Berberis_dasystachya:2.678992,Berberis_daochengensis:2.678992,Berberis
_concolor:2.678992,(((Berberis_vernae:0.765986,Berberis_amurensis:0.765986):1.02322,(Berberis_jul
ianae:1.138306,Berberis_sanguinea:1.138306):0.6509):0.292375,Berberis_poiretii:2.081581):0.597412)
:39.150379,(Dysosma_versipellis:2.753235,Dysosma_delavayi:2.753235):39.076137):0.002017,Sinop
odophyllum_hexandrum:41.831389):38.451668):17.915206,Circaeaster_agrestis:98.198263):14.73982
1,((((Meconopsis_paniculata:10.025653,Meconopsis_pinnatifolia:10.025653):25.745095,Meconopsis_
chelidoniifolia:35.770748):19.37404,(Hylomecon_japonica:45.705218,Eomecon_chionantha:45.70521
8):9.43957):32.053241,((Corydalis_zadoiensis:22.758246,Corydalis_wuzhengyiana:22.758246,Coryda
lis_uvaria:22.758246,Corydalis_sheareri:22.758246,Corydalis_rorida:22.758246,Corydalis_radicans:2
2.758246,Corydalis_raddeana:22.758246,Corydalis_quinquefoliolata:22.758246,Corydalis_pseudofilis
ecta:22.758246,Corydalis_pinnata:22.758246,Corydalis_pingwuensis:22.758246,Corydalis_nigroapicu
lata:22.758246,Corydalis_mucronata:22.758246,Corydalis_ludlowii:22.758246,Corydalis_longibractea
ta:22.758246,Corydalis_linstowiana:22.758246,Corydalis_iochanensis:22.758246,Corydalis_henderso
nii:22.758246,Corydalis_gracillima:22.758246,Corydalis_ellipticarpa:22.758246,Corydalis_conspersa:
22.758246,Corydalis_chrysosphaera:22.758246,Corydalis_cavei:22.758246,Corydalis_bulleyana:22.75
8246,Corydalis_bimaculata:22.758246,Corydalis_balsamiflora:22.758246,Corydalis_auriculata:22.758
246,Corydalis_adoxifolia:22.758246,((((Corydalis_hamata:2.539483,Corydalis_elata:2.539483):0.2859
65,Corydalis_flexuosa:2.825448):12.450929,(Corydalis_ternatifolia:2.361835,Corydalis_temulifolia:2.
361835):12.914542):5.188795,((Corydalis_edulis:7.831488,Corydalis_balansae:7.831488):3.630382,((
Corydalis_giraldii:2.161736,Corydalis_ophiocarpa:2.161736):1.074782,Corydalis_racemosa:3.236518)
:8.225352):9.003302):2.293075,Corydalis_adunca:22.758247):1.201755,(Dactylicapnos_torulosa:10.9
65408,Dactylicapnos_scandens:10.965408):12.994594):63.238027):25.740055):18.742616)eudicotyle
dons:2.742485,Ceratophyllum_demersum:134.423185)mrcaott2ott10930:1.33488,(((((((Frigidorchis_h
umidicola:66.482784,((((Cremastra_appendiculata:31.176492,(Calanthe_davidii:11.899843,Phaius_tan
kervilleae:11.899843):19.276649):1.055384,Liparis_petiolata:32.231875):11.804952,((Epipactis_humil
ior:7.679887,Epipactis_helleborine:7.679888):15.574835,Cephalanthera_longifolia:23.254723):20.782
105):9.85901,(((((Gymnadenia_conopsea:2.856331,Gymnadenia_orchidis:2.856331):7.124291,Dactylo
rhiza_umbrosa:9.980621):9.253508,(Amitostigma_trifurcatum:4.934892,Amitostigma_monanthum:4.9
34892,Amitostigma_gracile:4.934892,Amitostigma_basifoliatum:4.934893):14.299237):2.699064,(Ha
benaria_dentata:18.995304,(((Herminium_monorchis:3.79415,Peristylus_elisabethae:3.79415):1.85565
4,Peristylus_forceps:5.649804):12.06907,Peristylus_tentaculatus:17.718874):1.27643):2.93789):24.14
935,(Spiranthes_sinensis:32.583135,Goodyera_foliosa:32.583135):13.499409):7.813294):12.586947):
42.475952,(((((Polygonatum_kingianum:9.529609,Maianthemum_henryi:9.529609):0.636795,(((Ophi
opogon_angustifoliatus:4.326392,(((Ophiopogon_bodinieri:2.611174,Ophiopogon_japonicus:2.611174)
:0.39059,Ophiopogon_yunnanensis:3.001764):0.272066,Ophiopogon_intermedius:3.27383):1.052563):
0.728624,Liriope_kansuensis:5.055016):4.648477,Rohdea_wattii:9.703494):0.46291):52.326966,((Alli
um_yanchiense:24.753997,Allium_wallichii:24.753997,Allium_victorialis:24.753997,Allium_schoeno
prasum:24.753997,Allium_ovalifolium:24.753997,Allium_hookeri:24.753997,Allium_atrosanguineum:
24.753997,((((Allium_rude:10.816425,Allium_polyrhizum:10.816425):1.717979,Allium_trifurcatum:1
2.534404):0.413866,Allium_prattii:12.94827):1.26031,Allium_macranthum:14.20858):10.545418):30.
456017,Lycoris_aurea:55.210015):7.283355):18.164276,(Iris_farreri:22.865915,(((((Iris_japonica:2.85
9897,Iris_confusa:2.859897):0.58618,(Iris_wattii:0.858924,Iris_milesii:0.858924):2.587153):5.891659,
Iris_tectorum:9.337736):0.108114,Iris_subdichotoma:9.44585):8.75024,(((((((Iris_delavayi:0.734868,I

ris_chrysographes:0.734868):0.005673,Iris_bulleyana:0.740541):1.309698,Iris_clarkei:2.050239):0.00
7559,Iris_wilsonii:2.057798):6.162898,Iris_forrestii:8.220696):3.472853,Iris_laevigata:11.693549):5.8
6172,Iris_halophila:17.555269):0.640821):4.669826):57.79173):22.28506,Molineria_capitulata:102.94
2706):6.016031)Asparagales.rn.d8s.tre:5.641826,(((((Ptilagrostis_junatovii:47.740111,Deyeuxia_zangx
iensis:47.740111,((((((Panicum_khasianum:10.610029,Panicum_bisulcatum:10.61003):3.991501,((Pen
nisetum_flaccidum:6.01753,Setaria_plicata:6.017531):0.707312,Paspalidium_flavidum:6.724843):7.87
6688):2.004986,((Echinochloa_crus.galli:1.307076,(Echinochloa_glabrescens:0.040922,Echinochloa_o
ryzoides:0.040922):1.266155,Echinochloa_colona:1.307077):12.554717,(Sacciolepis_myosuroides:7.1
51136,Sacciolepis_indica:7.151137):6.710657):2.744723):4.212592,((((((((Saccharum_arundinaceum:
2.760166,Saccharum_spontaneum:2.760167):3.58646,(Microstegium_nudum:5.205041,Microstegium_
ciliatum:5.205041):1.141585):0.009975,Apluda_mutica:6.356602):0.338626,Pogonatherum_crinitum:
6.695228):1.434482,((Hemarthria_altissima:0.926734,Hemarthria_compressa:0.926734):6.735287,(Isc
haemum_rugosum:6.962804,Coix_aquatica:6.962804):0.699217):0.467689):6.904384,Arthraxon_hispi
dus:15.034094):2.277973,Arundinella_hirta:17.312067):2.850275,(Paspalum_longifolium:3.764196,Pa
spalum_thunbergii:3.764197):16.398145):0.656767):4.846477,(((((Leptochloa_chinensis:10.894758,L
eptochloa_fusca:10.894759):2.523021,Muhlenbergia_japonica:13.417779):3.99658,Eragrostis_japonic
a:17.41436):0.707151,Neyraudia_reynaudiana:18.121511):6.964491,((Isachne_clarkei:4.417242,((Isac
hne_globosa:1.538423,Isachne_albens:1.538423):0.002044,Isachne_sikkimensis:1.540467):2.876776):
19.119322,(Phragmites_karka:1.418588,Phragmites_australis:1.418589):22.117976):1.549437):0.5795
84):14.085492,((((((((Festuca_tristis:7.398515,Festuca_procera:7.398515,Festuca_nitidula:7.398515,F
estuca_leptopogon:7.398515,Festuca_japonica:7.398515,Festuca_forrestii:7.398515,Festuca_coelestis:
7.398515,(Festuca_extremiorientalis:3.306401,Festuca_parvigluma:3.306401):4.092115):5.300444,De
schampsia_cespitosa:12.69896):0.559832,(((((Alopecurus_arundinaceus:3.440174,Alopecurus_aequali
s:3.440174):0.765749,Beckmannia_syzigachne:4.205923):1.22582,(Poa_szechuensis:0.456145,Poa_su
pina:0.456145,Poa_sikkimensis:0.456145,Poa_pratensis:0.456145,Poa_palustris:0.456145,Poa_nubige
na:0.456145,Poa_nepalensis:0.456145,Poa_nemoralis:0.456145,Poa_lipskyi:0.456145,Poa_grandis:0.4
56145,Poa_calliopsis:0.456145,Poa_bomiensis:0.456145,Poa_binodis:0.456145,Poa_arctica:0.456145,
Poa_annua:0.456145,Poa_alpina:0.456145,Poa_acroleuca:0.456145,Poa_tibetica:0.456146):4.975597):
1.112012,(Phleum_alpinum:3.994429,Phleum_paniculatum:3.994429):2.549326):5.53857,((Puccinellia
_strictura:4.009556,Puccinellia_stapfiana:4.009556,Puccinellia_schischkinii:4.009556,Puccinellia_rob
orovskyi:4.009556,Puccinellia_przewalskii:4.009556,Puccinellia_poecilantha:4.009556,Puccinellia_pa
uciramea:4.009556,Puccinellia_pamirica:4.009556,Puccinellia_nudiflora:4.009556,Puccinellia_multifl
ora:4.009556,Puccinellia_minuta:4.009556,Puccinellia_micrandra:4.009556,Puccinellia_leiolepis:4.00
9556,Puccinellia_ladyginii:4.009556,Puccinellia_ladakhensis:4.009556,Puccinellia_himalaica:4.00955
6,Puccinellia_hauptiana:4.009556,Puccinellia_hackeliana:4.009556,Puccinellia_gigantea:4.009556,Puc
cinellia_dolicholepis:4.009556,Puccinellia_degeensis:4.009556,Puccinellia_arjinshanensis:4.009556,P
uccinellia_altaica:4.009556,(Puccinellia_chinampoensis:0.794627,(Puccinellia_distans:0.790911,Pucci
nellia_tenuiflora:0.790911):0.003716):3.21493):0.87194,Catabrosa_aquatica:4.881497):7.200828):1.17
6467):3.634498,((((((Agrostis_micrantha:3.288324,Agrostis_hookeriana:3.288324,(((Agrostis_clavata:
1.771285,Agrostis_vinealis:1.771285):0.00243,Agrostis_hugoniana:1.773715):1.175599,Agrostis_giga
ntea:2.949314):0.339011):0.001795,(((Polypogon_fugax:0.693908,Polypogon_monspeliensis:0.69390
8):1.251102,Polypogon_maritimus:1.94501):0.00887,Agropogon_lutosus:1.95388):1.33624):1.221596,
(Calamagrostis_purpurea:1.582831,Calamagrostis_pseudophragmites:1.582831,Calamagrostis_effusifl
ora:1.582831,(Calamagrostis_stricta:1.511109,(Calamagrostis_arundinacea:0.359009,Calamagrostis_e

pigeios:0.359009):1.1521):0.071723):2.928884):8.401144,Hierochloe_odorata:12.91286):0.244444,Ph
alaris_arundinacea:13.157304):0.004016,(Trisetum_bifidum:8.822463,(Helictotrichon_schmidii:8.819
658,Helictotrichon_junghuhnii:8.819658):0.002804):4.338857):3.73197):2.636326,(((Leymus_secalin
us:5.483472,(Elymus_pulanensis:2.82864,Elymus_burchan.buddae:2.82864,Elymus_nutans:2.828641):
2.654831):4.945464,Littledalea_racemosa:10.428935):0.011433,Bromus_japonicus:10.440369):9.0892
47):4.03786,(Melica_przewalskyi:15.32732,(Glyceria_tonglensis:8.456239,Glyceria_arundinacea:8.45
6239,Glyceria_acutiflora:8.456239,Glyceria_maxima:8.45624):6.871081):8.240155):0.282466,(((Stipa
_subsessiliflora:2.356619,Stipa_sareptana:2.356619,Stipa_hookeri:2.356619,Stipa_concinna:2.356619,
Stipa_bungeana:2.356619):3.113034,Piptatherum_laterale:5.469653):4.486269,Stipa_pappiformis:9.95
5923):13.894019):11.804834,Leersia_japonica:35.654776):4.096302):7.989034):41.342216,((Eriocaul
on_sollyanum:2.508515,Eriocaulon_setaceum:2.508515,Eriocaulon_nepalense:2.508515,Eriocaulon_
minusculum:2.508515,Eriocaulon_leianthum:2.508515,Eriocaulon_kunmingense:2.508515,Eriocaulon
_henryanum:2.508515,Eriocaulon_buergerianum:2.508515,Eriocaulon_alpestre:2.508515):74.619806,(
Xyris_pauciflora:29.4625735,Xyris_capensis:29.4625745):47.6657475):11.954006):4.739179,((((((((C
arex_yunnanensis:6.698533,Carex_yunlingensis:6.698533,Carex_wui:6.698533,Carex_vulpina:6.6985
33,Carex_thomsonii:6.698533,Carex_thibetica:6.698533,Carex_tangulashanensis:6.698533,Carex_sub
filicinoides:6.698533,Carex_stipitiutriculata:6.698533,Carex_shandanica:6.698533,Carex_setigera:6.6
98533,Carex_satakeana:6.698533,Carex_sagaensis:6.698533,Carex_rubrobrunnea:6.698533,Carex_roc
hebrunii:6.698533,Carex_remotiuscula:6.698533,Carex_pseudofoetida:6.698533,Carex_przewalskii:6.
698533,Carex_pamirensis:6.698533,Carex_ovatispiculata:6.698533,Carex_orbicularis:6.698533,Carex
_olivacea:6.698533,Carex_obscuriceps:6.698533,Carex_muliensis:6.698533,Carex_montis.everesti:6.6
98533,Carex_meyeriana:6.698533,Carex_melinacra:6.698533,Carex_maubertiana:6.698533,Carex_ma
quensis:6.698533,Carex_makuensis:6.698533,Carex_longispiculata:6.698533,Carex_longipes:6.69853
3,Carex_ligulata:6.698533,Carex_lehmannii:6.698533,Carex_lancisquamata:6.698533,Carex_laeta:6.6
98533,Carex_karlongensis:6.698533,Carex_ivanoviae:6.698533,Carex_ischnostachya:6.698533,Carex
_insignis:6.698533,Carex_inanis:6.698533,Carex_hongyuanensis:6.698533,Carex_henryi:6.698533,Ca
rex_haematostoma:6.698533,Carex_gonggaensis:6.698533,Carex_gentilis:6.698533,Carex_forrestii:6.
698533,Carex_fluviatilis:6.698533,Carex_finitima:6.698533,Carex_fargesii:6.698533,Carex_ensifolia:
6.698533,Carex_earistata:6.698533,Carex_duriuscula:6.698533,Carex_drepanorhyncha:6.698533,Care
x_dolichostachya:6.698533,Carex_delavayi:6.698533,Carex_cylindrostachys:6.698533,Carex_curaica:
6.698533,Carex_chlorostachys:6.698533,Carex_cespitosa:6.698533,Carex_cardiolepis:6.698533,Carex
_caespititia:6.698533,Carex_breviculmis:6.698533,Carex_aridula:6.698533,Carex_arcatica:6.698533,
Carex_amgunensis:6.698533,Carex_alta:6.698533,Carex_alba:6.698533,Carex_agglomerata:6.698533,
(((((((((Carex_phacota:0.904601,(Carex_dimorpholepis:0.801874,Carex_pruinosa:0.801874):0.102727)
:0.958906,Carex_atrofusca:1.863507):1.362642,Carex_capillaris:3.226149):0.384138,(Carex_baccans:
3.134263,Carex_lanceolata:3.134263):0.476024):0.340051,((((((Carex_obscura:0.888369,Carex_kansu
ensis:0.888369):0.001056,Carex_hancockiana:0.889425):0.55081,Carex_atrata:1.440235):0.052724,Ca
rex_moorcroftii:1.492959):2.192755,(Carex_japonica:2.076868,Carex_doniana:2.076868):1.608846):0.
26331,Carex_speciosa:3.949024):0.001314):0.387044,((Carex_filicina:3.075949,Carex_dielsiana:3.07
5949):0.003314,(Carex_scabrirostris:0.003469,Carex_coriophora:0.003469):3.075794):1.258119):0.56
4926,(Carex_cruciata:3.638262,(Carex_teinogyna:0.61397,Carex_brunnea:0.61397):3.024292):1.2640
46):1.024246,((Carex_enervis:3.102225,((Carex_leiorhyncha:2.207202,Carex_neurocarpa:2.207202):0.
472888,Carex_nubigena:2.68009):0.422135):0.726327,Carex_gibba:3.828552):2.098002):0.770258,Ca
rex_parva:6.696812):0.001722,((Carex_capillacea:0.668412,Carex_rara:0.668412):4.949471,Carex_mi

croglochin:5.617883):1.080651):0.002241,(Kobresia_vidua:6.303619,Kobresia_tunicata:6.303619,Kob
resia_tibetica:6.303619,Kobresia_setschwanensis:6.303619,Kobresia_robusta:6.303619,Kobresia_pusil
la:6.303619,Kobresia_macrantha:6.303619,Kobresia_loliacea:6.303619,Kobresia_littledalei:6.303619,
Kobresia_kansuensis:6.303619,Kobresia_hohxilensis:6.303619,Kobresia_graminifolia:6.303619,Kobre
sia_filifolia:6.303619,Kobresia_filicina:6.303619,Kobresia_duthiei:6.303619,Kobresia_cuneata:6.3036
19,((((Kobresia_pygmaea:0.983896,Kobresia_schoenoides:0.983896):1.027721,Kobresia_myosuroides:
2.011617):3.818425,Kobresia_fragilis:5.830042):0.298062,(Kobresia_capillifolia:5.820604,Kobresia_r
oyleana:5.820604):0.3075):0.175516,(Kobresia_laxa:5.88104,Kobresia_uncinioides:5.88104):0.42258):
0.397155):8.153875,(((Scirpus_rosthornii:4.098333,Scirpus_orientalis:4.098333,Scirpus_lushanensis:4.
098333,Scirpus_wichurae:4.098334):0.004262,Eriophorum_gracile:4.102596):7.482551,(Trichophoru
m_pumilum:0.003536,Trichophorum_distigmaticum:0.003536):11.581611):3.269503):1.262902,((Blys
mus_compressus:0.384581,Blysmus_sinocompressus:0.384581):5.807385,Blysmus_rufus:6.191966):9.
925586):10.316124,((((((((((Cyperus_pannonicus:5.051298,Cyperus_nigrofuscus:5.051298,Cyperus_e
xaltatus:5.051298,Cyperus_duclouxii:5.051298,((Cyperus_difformis:1.756675,Cyperus_fuscus:1.7566
75):3.102686,Cyperus_cuspidatus:4.859361):0.191938,(Cyperus_cyperoides:2.367805,Cyperus_amuri
cus:2.367805):2.683494):0.095,((Cyperus_pilosus:2.169494,Cyperus_compressus:2.169494):0.273449,
Cyperus_serotinus:2.442943):2.703356):0.002694,(Cyperus_squarrosus:3.333164,Kyllinga_squamulat
a:3.333164):1.815829):0.082987,(Cyperus_iria:0.527859,Cyperus_microiria:0.527859):4.704121):0.00
2882,(Pycreus_unioloides:4.661381,Pycreus_lijiangensis:4.661381,Pycreus_delavayi:4.661381,(Pycre
us_sanguinolentus:3.228299,Pycreus_flavidus:3.228299):1.433083):0.57348):0.109612,Kyllinga_brevi
folia:5.344474):0.647205,(Lipocarpha_microcephala:3.428388,Lipocarpha_chinensis:3.428388):2.563
291):7.872598,Isolepis_setacea:13.864277):6.533125,(((Schoenoplectiella_mucronata:1.30716,Schoen
oplectiella_juncoides:1.30716):16.694602,(Schoenoplectus_subulatus:14.294657,(Schoenoplectus_tab
ernaemontani:0.155768,Schoenoplectus_triqueter:0.155768):14.13889):3.707104):0.834568,Bolboscho
enus_planiculmis:18.83633):1.561072):2.861776,((Eleocharis_yunnanensis:18.97514,Eleocharis_qing
haiensis:18.97514,Eleocharis_penchaoi:18.97514,Eleocharis_fennica:18.97514,Eleocharis_erhaiensis:
18.97514,((((Eleocharis_palustris:0.157413,Eleocharis_valleculosa:0.157413):2.24525,Eleocharis_ovat
a:2.402663):5.432047,(Eleocharis_uniglumis:5.367173,Eleocharis_yokoscensis:5.367173):2.467537):2.
471171,Eleocharis_quinqueflora:10.305881):8.66926):1.120343,((Fimbristylis_stolonifera:8.533386,Fi
mbristylis_nigrobrunnea:8.533386,Fimbristylis_henryi:8.533386,Fimbristylis_bisumbellata:8.533386,(
((Fimbristylis_aestivalis:2.364236,Fimbristylis_dichotoma:2.364236):2.096585,Fimbristylis_complana
ta:4.460821):0.520994,(Fimbristylis_littoralis:2.501623,Fimbristylis_quinquangularis:2.501623):2.480
192):3.551572,Fimbristylis_ovata:8.533387):3.557755,Bulbostylis_densa:12.091142):8.004342):3.163
694):3.174498):12.976738,Cladium_mariscus:39.410414):1.742873,(Scleria_hookeriana:20.835659,Sc
leria_biflora:20.835659,(Scleria_terrestris:9.951142,Scleria_parvula:9.951142):10.884518):20.317627):
14.007693,((Juncus_thomsonii:36.605208,Juncus_sphacelatus:36.605208,Juncus_sikkimensis:36.6052
08,Juncus_setchuensis:36.605208,Juncus_rohtangensis:36.605208,Juncus_przewalskii:36.605208,Junc
us_prismatocarpus:36.605208,Juncus_potaninii:36.605208,Juncus_nepalicus:36.605208,Juncus_modic
us:36.605208,Juncus_minimus:36.605208,Juncus_libanoticus:36.605208,Juncus_leucomelas:36.60520
8,Juncus_leucanthus:36.605208,Juncus_leptospermus:36.605208,Juncus_himalensis:36.605208,Juncus
_heptopotamicus:36.605208,Juncus_grisebachii:36.605208,Juncus_gracilicaulis:36.605208,Juncus_gig
anteus:36.605208,Juncus_concinnus:36.605208,Juncus_clarkei:36.605208,Juncus_amplifolius:36.6052
08,Juncus_allioides:36.605208,Juncus_alatus:36.605208,(((((Juncus_ranarius:2.555459,Juncus_bufoni
us:2.555459):5.317992,Juncus_tenuis:7.873451):6.766493,Juncus_compressus:14.639944):2.637009,(J

uncus_effusus:6.235109,Juncus_inflexus:6.235109):11.041844):15.889722,(((Juncus_wallichianus:0.0
04504,Juncus_diastrophanthus:0.004504):8.555425,Juncus_articulatus:8.559929):9.318704,(Juncus_tri
glumis:4.180604,Juncus_castaneus:4.180604):13.698029):15.288042):3.438534):7.016196,(Luzula_pl
umosa:12.812848,Luzula_effusa:12.812848,Luzula_multiflora:12.812849):30.808556):11.539575):38.
660527):7.154022,((Sparganium_limosum:10.4188,Sparganium_confertum:10.4188,((Sparganium_em
ersum:6.556157,(Sparganium_stoloniferum:5.275524,Sparganium_glomeratum:5.275524):1.280633):0.
004807,Sparganium_fallax:6.560964):3.857837):43.882175,(Typha_lugdunensis:19.649673,Typha_da
vidiana:19.649673,((((Typha_latifolia:2.275283,Typha_domingensis:2.275283):0.700983,Typha_laxm
annii:2.976266):0.007522,Typha_orientalis:2.983788):7.363823,Typha_angustifolia:10.347611):9.302
063,Typha_minima:19.649674):34.651302):46.674553)Poales.rn.d8s.tre:7.259624,((Zingiber_striolatu
m:58.260569,Musa_rubra:58.260568)Zingiberales.rn.d8s.tre:21.542656,(Monochoria_vaginalis:68.548
014,((Cyanotis_arachnoidea:7.34539,Cyanotis_cristata:7.345391):28.110089,(((Commelina_maculata:
6.125974,Commelina_communis:6.125975,(Commelina_paludosa:4.580185,Commelina_benghalensis:
4.580185):1.54579):0.021708,Floscopa_scandens:6.147683):20.903067,(Murdannia_triquetra:17.9001
43,Murdannia_simplex:17.900143,Murdannia_loriformis:17.900143,Murdannia_nudiflora:17.900144):
9.150606):8.40473):33.092534)Commelinales.rn.d8s.tre:11.255211)mrcaott121ott3449:28.431928)mrc
aott121ott252:6.36541)mrcaott121ott334:2.276244,(((Fritillaria_sichuanica:2.396714,Fritillaria_cirrho
sa:2.396714):8.431594,Fritillaria_davidii:10.828308):44.747878,Smilax_glabra:55.576186):61.300621)
mrcaott121ott1439:2.895004,(Tacca_chantrieri:95.232997,(Aletris_pauciflora:25.759648,((Aletris_ste
noloba:12.263265,(Aletris_gracilis:11.725085,Aletris_alpestris:11.725085):0.53818):1.188735,Aletris_
laxiflora:13.452):12.307649):69.473348)Dioscoreales.rn.d8s.tre:24.538814)mrcaott121ott2256:11.026
948,(((((Arisaema_griffithii:89.141365,Pinellia_pedatisecta:89.141365):0.037604,Sauromatum_gigant
eum:89.178969):31.228579,Lasia_spinosa:120.407548):1.243204,(Spirodela_polyrhiza:73.079775,(W
olffia_arrhiza:58.920148,((Lemna_japonica:16.673081,Lemna_trisulca:16.673081):5.833578,Lemna_
minor:22.506659):36.413489):14.159628):48.570976):7.210626,(((((((((((Potamogeton_wrightii:1.73
6175,(Potamogeton_nodosus:1.541937,Potamogeton_distinctus:1.541937):0.194238):0.758123,Potam
ogeton_perfoliatus:2.494298):1.658855,(Potamogeton_lucens:0.585781,Potamogeton_gramineus:0.58
5781):3.567372):0.289358,Potamogeton_crispus:4.442511):0.867911,((((Potamogeton_oxyphyllus:0.7
30214,Potamogeton_pusillus:0.730214):1.280826,(Potamogeton_compressus:0.284106,Potamogeton_
obtusifolius:0.284106):1.726934):0.651609,Potamogeton_octandrus:2.662649):2.031395,Potamogeton
_natans:4.694044):0.616378):13.230559,Potamogeton_maackianus:18.540981):12.337144,Zannichelli
a_palustris:30.878125):0.337349,((Stuckenia_amblyophylla:1.894549,Stuckenia_filiformis:1.894549):
2.959084,(Stuckenia_pectinata:3.573687,Stuckenia_pamirica:3.573687):1.279946):26.361841):28.427
772,Ruppia_maritima:59.643246):9.114296,(Triglochin_maritima:6.908515,Triglochin_palustris:6.908
515):61.849027):10.828654,Scheuchzeria_palustris:79.586196):17.047052,(((((Sagittaria_pygmaea:2.5
45583,Sagittaria_tengtsungensis:2.545583):3.004321,Sagittaria_trifolia:5.549904):29.329871,Caldesia
_parnassifolia:34.879775):5.294355,(Alisma_plantago.aquatica:3.614442,Alisma_orientale:3.614442,
Alisma_canaliculatum:3.614443,Alisma_gramineum:3.614443):36.559687):31.880621,(((Vallisneria_n
atans:35.886299,Hydrilla_verticillata:35.886299):8.235652,(Najas_marina:11.809955,Najas_minor:11.
809955):32.311996):0.821885,(Ottelia_acuminata:16.151401,(Blyxa_echinosperma:5.495879,Blyxa_j
aponica:5.495879):10.655522):28.792435):27.110915):24.578497):32.22813)Alismatales.rn.d8s.tre:1.9
37381)mrcaott121ott290:2.435645,(Acorus_calamus:30.000014,Acorus_gramineus:30.000014)Acoral
es.rn.d8s.tre:103.23439)Liliopsida:2.523661)mrcaott2ott121:0.154122,((Phoebe_hunanensis:132.3938
8,((Saururus_chinensis:44.501597,Houttuynia_cordata:44.501597):60.941422,((Asarum_caudigerellu

m:4.693971,Asarum_himalaicum:4.693971):2.042015,(Asarum_cardiophyllum:1.038004,Asarum_caudigerum:1.038004):5.697982):98.707033)Piperales.rn.d8s.tre:26.950862)Magnoliidae:2.226081,(Chloranthus_multistachys:44.881395,(Chloranthus_serratus:38.540722,Chloranthus_elatior:38.540722):6.340674):89.738566)mrcaott890ott34978:1.292225)Mesangiospermae:3.052834,(Nymphaea_tetragona:109.574477,Brasenia_schreberi:109.574477):29.390544)mrcaott2ott2645;
