# Supplementary material for: Large-scale geographic patterns and environmental and anthropogenic drivers of wetland plant diversity in the Qinghai-Tibet Plateau
Source: BMC Ecol Evol. 2024 Jun 3;24:74. doi: 10.1186/s12862-024-02263-w (PMC11145778; doi:10.1186/s12862-024-02263-w)
Supplement: Supplementary file 5 — Supplementary Material 5 [file 12862_2024_2263_MOESM5_ESM.pdf]

Additional file 5.

The Newick version of the functional dendrogram of wetland plants in the Qinghai-Tibet Plateau based on six morphological traits

((Wolffia_arrhiza:0.05288461538,(Spirodela_polyrhiza:0.02272727273,(Lemna_japonica:0,Lemna_minor:0):0.02272727273):0.03015734266):0.4471153846,((((Callitriche_palustris:0.1166078651,Ranunculus_natans:0.1166078651):0.1658875711,((((((Potentilla_supina:0.02564102564,(Ixeris_chinensis:0.01777389277,(Grangea_maderaspatana:0.004953379953,Taraxacum_scariosum:0.004953379953):0.01282051282):0.007867132867):0.01591171886,((Potentilla_kleiniana:0.01312605248,(Potentilla_griffithii:0.003470520444,Potentilla_leuconota:0.003470520444):0.009655532038):0.01302160328,(Potentilla_flagellaris:0.01331532429,((Artemisiella_stracheyi:0.001420903417,Pseudognaphalium_affine:0.001420903417):0.005787358405,(Polygonum_kawagoeanum:0.002436542554,Potentilla_discolor:0.002436542554):0.004771719267):0.006107062465):0.01283233147):0.01540508874):0.02313257019,(((Carpesium_minus:0.002185314685,Potentilla_angustiloba:0.002185314685):0.03538372597,((Sibbaldianthe_bifurca:0.005807363859,(Halerpestes_tricuspis:0.0005827505828,Ranunculus_membranaceus:0.0005827505828):0.005224613277):0.0118785898,(Potentilla_sericea:0.006162465278,(Sibbaldia_parviflora:0.004585884199,(Potentilla_anserina:0,Potentilla_multicaulis:0):0.004585884199):0.001576581078):0.01152348838):0.019883087):0.01918641425,(((Valeriana_flaccidissima:0.008304195804,(Artemisia_velutina:0.005897073011,Duchesnea_indica:0.005897073011):0.002407122794):0.01373822285,((Potentilla_lancinata:0.005202941121,(Crepis_rigescens:0.001311188811,Potentilla_virgata:0.001311188811):0.003891752309):0.01146319902,(Artemisia_capillaris:0.008741258741,(Agrimonia_pilosa:0.001165501166,Senecio_wightii:0.001165501166):0.007575757576):0.007924881396):0.00537627852):0.01828286858,(((Potentilla_saundersiana:0.007513801483,(Heteropappus_gouldii:0.002185314685,Potentilla_potaninii:0.002185314685):0.005328486798):0.01104627802,((Potentilla_multifida:0.006252750083,(Taraxacum_maurocarpum:0.003364980221,(Ranunculus_ficariifolius:0.0004370629371,Ranunculus_trigonus:0.0004370629371):0.002927917284):0.002887769862):0.007062574176,(Anemone_trullifolia:0.006661484307,(Ranunculus_albertii:0.001748251748,Ranunculus_yunnanensis:0.001748251748):0.004913232559):0.006653839952):0.005244755245):0.01280292499,(((Duchesnea_chrysantha:0.0005827505828,Potentilla_centigrana:0.0005827505828):0.01024171933,(Potentilla_gelida:0.005827505828,Potentilla_plumosa:0.005827505828):0.004996964084):0.0101981352,((Ranunculus_pedatifidus:0.005078925603,(Ranunculus_nematolobus:0.002296695965,(Askellia_flexuosa:0.0008741258741,(Potentilla_simulatrix:0,Senecio_dubitabilis:0):0.0008741258741):0.001422570091):0.002782229638):0.007799335719,((Ranunculus_tanguticus:0.003787878788,(Halerpestes_ruthenica:0.001456876457,Ranunculus_potaninii:0.001456876457):0.002331002331):0.004922200291,(Ranunculus_distans:0.00707089749,((Neobrachyactis_roylei:0,Potentilla_delavayi:0):0.003506491473,(Erigeron_moupinensis:0.001223776224,Pouzolzia_zeylanica:0.001223776224):0.00228271525):0.003564406017):0.001639181589):0.004168182243):0.008144343788):0.01034039939):0.008962282745):0.01643016766):0.007929859786):0.03826728827,(((((Ranunculus_sinovaginatus:0.007645324759,(Potentilla_fragarioides:0.003205128205,(Cremanthodium_smithianum:0.0005827505828,Taraxacum_mongolicum:0.0005827505828):0.002622377622):0.004440196554):0.008248792991,(Ligularia_microcephala:0.00829237715,Taraxacu

m_eriopodum:0.00829237715):0.0076017406):0.01584741708,(Tussilago_farfara:0.02817546415,
(Cremanthodium_glandulipilosum:0.01126018508,Ligularia_potaninii:0.01126018508):0.016915
27906):0.00356607068):0.01803793725,((((Askellia_karelinii:0,Potentilla_multiceps:0):0.010940
7378,(Eschenbachia_muliensis:0.004170742261,Ranunculus_trautvetterianus:0.004170742261):0.
006769995536):0.01425901734,((Senecio_yungningensis:0.008228075342,(Polygonum_forrestii:
0.005328486798,Potentilla_hypargyrea:0.005328486798):0.002899588544):0.01201829985,((Art
emisia_macrocephala:0.003947730804,Ranunculus_indivisus:0.003947730804):0.004877259491,
(Senecio_albopurpureus:0.005641670787,(Ranunculus_popovii:0.001893939394,Ranunculus_pul
chellus:0.001893939394):0.003747731393):0.003183319507):0.01142138489):0.004953379953):
0.01098852952,((Ranunculus_dielsianus:0.01323159273,(Ajania_khartensis:0.003205128205,Ra
nunculus_pegaeus:0.003205128205):0.01002646453):0.01245301707,(Ranunculus_kamchaticus:
0.01448082545,(Ranunculus_trivedii:0.008547779374,(Artemisia_kuschakewiczii:0.0027822296
38,Ranunculus_furcatifidus:0.002782229638):0.005765549735):0.005933046079):0.0112037843
5):0.01050367486):0.01359118742):0.03021509119,(Pseudoyoungia_simulatrix:0.06677848266,(
(Halerpestes_filisecta:0.01800331197,Ranunculus_banguoensis:0.01800331197):0.01723472632,(
Ranunculus_pseudopygmaeus:0.02497033594,(Potentilla_stenophylla:0.01519509931,(Oxygraphi
s_tenuifolia:0.01098432196,(Halerpestes_lancifolia:0.004585884199,Ranunculus_brotherusii:0.0
04585884199):0.006398437756):0.004210777355):0.009775236631):0.01026770235):0.0315404
4437):0.0132160806):0.02295803969):0.04708887503,((((Clematis_tangutica:0.02789590751,(Cl
ematis_rehderiana:0.01038740697,Senecio_scandens:0.01038740697):0.01750850053):0.024648
77004,((Potentilla_parvifolia:0.01274439236,Thalictrum_cultratum:0.01274439236):0.02960635
755,(((Cremanthodium_stenoglossum:0.005820952548,Sonchella_dentata:0.005820952548):0.01
034382284,(Cremanthodium_pulchrum:0.005897073011,(Potentilla_conferta:0.001271041389,Ra
nunculus_sceleratus:0.001271041389):0.004626031622):0.01026770238):0.01142138489,(Thalict
rum_simplex:0.01768595366,(Bidens_tripartita:0.00931219067,(Crepis_bodinieri:0.00741825127
6,(Sanguisorba_alpina:0.003213451519,(Bidens_maximowicziana:0,Inula_caspica:0):0.00321345
1519):0.004204799757):0.001893939394):0.008373762987):0.009900206627):0.01476458962):0.
01019392764):0.02020699859,(((Potentilla_freyniana:0.01377239262,((Ligularia_latihastata:0.00
1171342222,Ligularia_vellerea:0.001171342222):0.009006650981,(Cremanthodium_angustifoliu
m:0.003893419011,Cremanthodium_lineare:0.003893419011):0.006284574191):0.00359439942):
0.01945434737,(((Inula_britannica:0.00793670005,((Ranunculus_japonicus:0.001165501166,Ran
unculus_repens:0.001165501166):0.004198958701,(Pilea_notata:0.002193637999,Ranunculus_ca
ntoniensis:0.002193637999):0.003170821867):0.002572240184):0.00573195562,(Artemisia_caru
ifolia:0.01086461733,(Geum_aleppicum:0.001748251748,(Ligularia_hodgsonii:0,Ligularia_retus
a:0):0.001748251748):0.009116365586):0.002804038336):0.01262048017,((Pilea_anisophylla:0.
005869708633,(Carpesium_cernuum:0,Potentilla_reptans:0):0.005869708633):0.01489161999,((
Tephroseris_stolonifera:0.005253078559,(Bidens_cernua:0.003323166097,Sonchus_wightianus:0.
003323166097):0.001929912462):0.01079433793,((Ligularia_virgaurea:0.004239106095,(Bidens
_parviflora:0,Cremanthodium_stenactinium:0):0.004239106095):0.008236398656,(Pilea_pumila:
0.004877259491,(Ranunculus_diffusus:0.003359139165,Saussurea_involucrata:0.003359139165):
0.001518120326):0.00759824526):0.003571911736):0.004713912135):0.005527807222):0.00693
7604152):0.02041387153,(((((Pilea_monilifera:0.001165501166,Urtica_cannabina:0.0011655011
66):0.008741258741,(Elatostema_platyphyllum:0.003350815851,Ligularia_phyllocolea:0.003350
815851):0.006555944056):0.01029861306,((Rumex_pseudonatronatus:0.004724520754,(Ligulari

a_atkinsonii:0.003121956652,(Ligularia_tongolensis:0.001602564103,(Ligularia_liatroides:0.000
7284382284,Ligularia_subspicata:0.0007284382284):0.0008741258741):0.001519392549):0.001
602564103):0.006114640085,(Cremanthodium_chungdienense:0.007695527296,((Rumex_obtusif
olius:0.002622377622,(Rumex_longifolius:0.00101981352,Rumex_patientia:0.00101981352):0.0
01602564103):0.00386315109,(Rumex_gmelinii:0.003146853147,(Ligularia_konkalingensis:0.00
2582230228,Parasenecio_pilgerianus:0.002582230228):0.000564622919):0.003338675566):0.00
1209998584):0.003143633543):0.00936621213):0.00750514812,(((Ligularia_lamarum:0.002035
565014,Ligularia_microcardia:0.002035565014):0.003911645408,(Ligularia_lidjiangensis:0.0036
1620809,Rumex_chalepensis:0.00361620809):0.002331002331):0.01592353754,(Cremanthodiu
m_petiolatum:0.01056296338,Parasenecio_hastiformis:0.01056296338):0.01130778458):0.00583
9773129):0.02125202466,(((Ligularia_cymbulifera:0.003274695388,Ligularia_purdomii:0.00327
4695388):0.01687378388,(Ligularia_transversifolia:0.00879900727,(Inula_racemosa:0.00536445
9866,Ligularia_oligonema:0.005364459866):0.003434547404):0.01134947199):0.01327174013,(
(Ligularia_duciformis:0.0118537579,(Ligularia_franchetiana:0.007735609592,Sonchus_palustris:
0.007735609592):0.004118148308):0.0120549504,(((Ligularia_caloxantha:0.002997239922,(Lig
ularia_fischeri:0.001748251748,Ligularia_rumicifolia:0.001748251748):0.001248988174):0.0047
08500939,(Ligularia_intermedia:0.003917862074,Ligularia_sibirica:0.003917862074):0.0037878
78788):0.009024007659,(Ligularia_sagitta:0.01227561764,(Ligularia_przewalskii:0.0070986595
45,((Ligularia_ghatsukupa:0.0005827505828,Ligularia_veitchiana:0.0005827505828):0.0052589
19615,(Dipsacus_asper:0.002185314685,Ligularia_tenuipes:0.002185314685):0.003656355512):
0.001256989347):0.005176958096):0.004454130879):0.007178959778):0.009511511094):0.0155
4232636):0.004678065779):0.01911106461):0.04271641987,(Sinosenecio_oldhamianus:0.07655
558117,((Ligularia_rockiana:0.02191979285,((Ligularia_japonica:0.007575757576,Ligularia_plat
yglossa:0.007575757576):0.01044936309,(Ligularia_pleurocaulis:0.01021229957,(Ligularia_sten
ocephala:0.004636021638,Senecio_graciliflorus:0.004636021638):0.005576277931):0.00781282
1102):0.003894672177):0.02447844566,((Acmella_paniculata:0.01594952056,(Ranunculus_sileri
folius:0.00702904516,Senecio_nemorensis:0.00702904516):0.008920475403):0.01203083072,((I
nula_japonica:0.006118881119,Youngia_japonica:0.006118881119):0.01147501767,(Gynura_bico
lor:0.01025588373,(Geum_japonicum:0.007735609592,Ranunculus_sieboldii:0.007735609592):0.
002520274136):0.007338015063):0.0103864525):0.01841788723):0.03015734266):0.038912514
84):0.03457338198):0.09162110445,(((Berberis_dasystachya:0.07648338128,(((Acorus_gramine
us:0.02566812104,(Berberis_obovatifolia:0.003929634466,Corydalis_quinquefoliolata:0.0039296
34466):0.02173848657):0.02105745256,((Berberis_longispina:0.02017446229,(Berberis_haoi:0.0
02476689977,Berberis_ulicina:0.002476689977):0.01769777231):0.01943420541,((Berberis_jiul
ongensis:0.01517861058,(Berberis_concolor:0.01036698631,((Berberis_daochengensis:0.000699
3006993,Berberis_racemulosa:0.0006993006993):0.009059729462,(Berberis_tenuipedicellata:0.0
03642191142,(Berberis_potaninii:0.0002913752914,Berberis_vernae:0.0002913752914):0.00335
0815851):0.006116839019):0.0006079561443):0.004811624275):0.01387097944,(Berberis_dicty
ophylla:0.01681182778,(Berberis_davidii:0.005099067599,Berberis_pseudotibetica:0.005099067
599):0.01171276018):0.01223776224):0.01055907767):0.0071169059):0.02792477606,(((Berberi
s_jamesiana:0.009032634033,Berberis_julianae:0.009032634033):0.01264051881,(Berberis_amu
rensis:0.01257856272,(Berberis_feddeana:0.007513801483,(Berberis_xanthophlaea:0.003059440
559,(Berberis_dielsiana:0.00101981352,Berberis_poiretii:0.00101981352):0.00203962704):0.004
454360924):0.005064761233):0.009094590125):0.0317138851,(Molineria_capitulata:0.03531468

531,(Maianthemum_henryi:0.01328671329,Rohdea_wattii:0.01328671329):0.02202797203):0.01
807235263):0.02126331171):0.001833031634):0.08689940401,(((Kyllinga_squamulata:0.019288
51773,((Carex_rara:0.004490333964,Elsholtzia_luteola:0.004490333964):0.008158508159,(Care
x_setigera:0.0048709345,(Carex_delavayi:0.003205128205,(Carex_chlorostachys:0.00263937486
7,Trichophorum_distigmaticum:0.002639374867):0.0005657533385):0.001665806295):0.007777
907623):0.006639675609):0.04273006435,(Scirpus_rosthornii:0.03792237204,((Cyperus_microir
ia:0.01747069883,(((Carex_dolichostachya:0.001165501166,Carex_doniana:0.001165501166):0.0
03787878788,(Cyperus_difformis:0.001985427603,Scleria_biflora:0.001985427603):0.00296795
235):0.006056925027,(Carex_maubertiana:0.005063094531,Carex_teinogyna:0.005063094531):0.
005947210449):0.006460393848):0.008691121303,((Carex_finitima:0.007112711614,((Carex_ag
glomerata:0,Carex_ischnostachya:0):0.006639675609,(Carex_leiorhyncha:0.002295029263,(Care
x_yunnanensis:0.001126606909,Pycreus_unioloides:0.001126606909):0.001168422354):0.00434
4646346):0.0004730360051):0.01345268862,(Carex_ligulata:0.01244119841,(Carex_henryi:0.00
7992678545,Carex_neurocarpa:0.007992678545):0.004448519868):0.00812420182):0.00559641
9899):0.01176055191):0.02409621004):0.0827765658,((Barbarea_intermedia:0.02547696606,(R
orippa_elata:0.01539853546,(Rorippa_dubia:0.006821336295,Rorippa_indica:0.006821336295):
0.008577199162):0.0100784306):0.1053321678,(Cyclorhiza_waltonii:0.05476018285,(Pleurospe
rmum_hookeri:0.03001165501,(Galium_verum:0.01690809021,(Bupleurum_gracillimum:0.0055
4445385,Chamaesium_paradoxum:0.00554445385):0.01136363636):0.0131035648):0.024748527
83):0.07604895105):0.01398601399):0.01858763742):0.03643413211,((((Caltha_scaposa:0.0168
2364644,(Phedimus_odontophyllus:0.006264568765,Trollius_ranunculoides:0.006264568765):0.
01055907767):0.01365683699,(Caltha_palustris:0.02613583711,(Trollius_yunnanensis:0.003907
583409,Vincetoxicum_forrestii:0.003907583409):0.0222282537):0.004344646319):0.038454826
28,((Rhodiola_sherriffii:0.01030951648,Sedum_triactina:0.01030951648):0.03117130187,(Sedu
m_fedtschenkoi:0.03825048455,((Sedum_tsinghaicum:0.005869708633,Trollius_pumilus:0.0058
69708633):0.01311326946,(Sedum_chauveaudii:0.009775236631,Sedum_obtusipetalum:0.00977
5236631):0.009207741467):0.01926750645):0.0032303338):0.02745449136):0.1084954457,((Dr
aba_involucrata:0.03987405993,Draba_oreades:0.03987405993):0.04810448099,((Draba_stenoca
rpa:0.02226422674,(Rorippa_cantoniensis:0.01928851773,Rorippa_palustris:0.01928851773):0.0
02975709006):0.0231023796,(Rorippa_globosa:0.02410453338,(Draba_nemorosa:0.0165682441
9,(Draba_eriopoda:0.006342459262,Draba_yunnanensis:0.006342459262):0.01022578492):0.007
536289196):0.02126207296):0.04261193459):0.08945221445):0.02238616203):0.04184566503):
0.04083285376):0.1114439577,(((Ludwigia_taiwanensis:0.1043123543,Nymphoides_peltata:0.10
43123543):0.1155738872,(Limnophila_sessiliflora:0.1315517365,((Utricularia_gibba:0.01590404
18,(Utricularia_aurea:0,Utricularia_australis:0):0.0159040418):0.03806691954,((Eriocaulon_seta
ceum:0.01171328671,Utricularia_minor:0.01171328671):0.02356516986,(Utricularia_intermedia:
0.02051597502,Utricularia_vulgaris:0.02051597502):0.01476248156):0.01869250476):0.077580
77512):0.08833450504):0.1134470918,((((((Triglochin_palustris:0.0100264645,(Ammannia_auri
culata:0.00307360493,Murdannia_nudiflora:0.00307360493):0.006952859571):0.01411753726,(
Lindernia_micrantha:0.01944602406,Murdannia_simplex:0.01944602406):0.004697977702):0.01
960166851,((Lindernia_procumbens:0.0171291861,((Pedicularis_anas:0,(Corydalis_adoxifolia:0,
Corydalis_linstowiana:0):0):0.009090909091,(Corydalis_pinnata:0.002989025216,Lindernia_pusi
lla:0.002989025216):0.006101883875):0.008038277008):0.01646270396,(Gentiana_helophila:0.0
3092936504,(Veronica_beccabunga:0.01107226107,Viola_prionantha:0.01107226107):0.0198571

0397):0.002662525017):0.01015378021):0.04118601957,((Viola_inconspicua:0.01223776224,(Li
ndernia_antipoda:0.00561986209,Lindernia_ciliata:0.00561986209):0.006617900148):0.0332167
8322,(Lindernia_crustacea:0.02621956863,Lobelia_zeylanica:0.02621956863):0.01923497682):0.
03947714439):0.07143616746,(((((((Viola_diffusa:0.0001748251748,(Gentiana_amplicrater:0,Ma
zus_lecomtei:0):0.0001748251748):0.009090909091,(Viola_forrestiana:0.004781709283,Viola_s
elkirkii:0.004781709283):0.004484024982):0.009972646056,(Henckelia_forrestii:0.00995618512
2,(Primula_woodwardii:0.003857445971,Viola_thomsonii:0.003857445971):0.006098739151):0.
0092821952):0.01208025594,((Primula_optata:0.01062337948,(Primula_laxiuscula:0.003747843
694,(Primula_forbesii:0.001548364638,Primula_tsariensis:0.001548364638):0.002199479056):0.
006875535787):0.01475803637,(Viola_pendulicarpa:0.02026474712,(Primula_algida:0.00857542
9127,Primula_macrophylla:0.008575429127):0.01168931799):0.005116668734):0.005937220405)
:0.01466832719,((Epilobium_kingdonii:0.01206293706,(Juncus_rohtangensis:0,Salix_caesia:0):0.
01206293706):0.02477826909,((Primula_silaensis:0.0076816307,(Pedicularis_variegata:0.00716
7832168,(Pedicularis_elliotii:0.003496503497,(Pedicularis_aschistorrhyncha:0,Salix_divergentist
yla:0):0.003496503497):0.003671328671):0.0005137985322):0.01322304297,(Amitostigma_mon
anthum:0.0154864746,Primula_souliei:0.0154864746):0.005418199067):0.01593653249):0.0091
45757296):0.03367550931,((Myricaria_prostrata:0.0215635416,(Lindernia_nummulariifolia:0.00
776502938,Swertia_hispidicalyx:0.00776502938):0.01379851222):0.03624755435,(Primula_sapp
hirina:0.03326600429,(Corydalis_uvaria:0.02239154248,(Pedicularis_pygmaea:0.01438877058,(
Gentiana_asparagoides:0.004953379953,Pedicularis_yui:0.004953379953):0.009435390631):0.00
8002771894):0.01087446181):0.02454509166):0.02185137682):0.03767448904,((Myricaria_war
dii:0.04347266694,(Rhododendron_nivale:0.01837080773,(Rhododendron_capitatum:0.0059731
93473,Rhododendron_xiguense:0.005973193473):0.01239761425):0.02510185922):0.048795312
69,(((Buddleja_officinalis:0.02887371176,((Impatiens_cymbifera:0.009032634033,(Iris_milesii:0.
004330481976,Iris_wattii:0.004330481976):0.004702152057):0.01040157137,((Primula_secundi
flora:0.005508480782,(Impatiens_fragicolor:0.00203962704,Impatiens_purpurea:0.00203962704):
0.003468853743):0.01144903462,(Iris_chrysographes:0.007589921946,(Iris_tectorum:0.0041989
58673,Primula_anisodora:0.004198958673):0.003390963273):0.009367593455):0.002476690004)
:0.009439506355):0.01675407925,((Veronica_laxa:0.01830467728,((Impatiens_aquatilis:0.00262
2377622,Impatiens_taronensis:0.002622377622):0.008318360174,(Pedicularis_pantlingii:0.00405
3271027,(Epilobium_subcoriaceum:0.002436542554,Impatiens_abbatis:0.002436542554):0.0016
16728473):0.00688746677):0.007363939483):0.01255091299,((Primula_limbata:0.00586970863
3,(Asarum_caudigerellum:0,Mazus_pumilus:0):0.005869708633):0.01756763036,((Primula_cald
aria:0.005071417873,(Iris_subdichotoma:0.002047950354,Primula_nivalis:0.002047950354):0.00
3023467519):0.006661484279,(Impatiens_recurvicornis:0.005495983114,(Gentiana_decumbens:
0.001893939394,Primula_capitata:0.001893939394):0.00360204372):0.006236919038):0.011704
43684):0.007418251276):0.01477220074):0.01700530712,((((Oldenlandia_herbacea:0,Pediculari
s_rhinanthoides:0):0.01501415082,(Pedicularis_deqinensis:0.004723248531,(Primula_nutans:0.0
02381139741,(Impatiens_tuberculata:0.001295484451,Pedicularis_strobilacea:0.001295484451):
0.00108565529):0.00234210879):0.01029090229):0.01798494004,(Rhododendron_hippophaeoid
es:0.01602564103,(Rhododendron_nitidulum:0.01238344988,Rhododendron_websterianum:0.01
238344988):0.003642191142):0.01697344983):0.02677222047,(((Astilbe_rubra:0.00981882079,
Buddleja_lindleyana:0.00981882079):0.01238344988,(Iris_delavayi:0.01531136717,(Lythrum_sa
licaria:0.004093418449,(Lobelia_sessilifolia:0.002331002331,Verbena_officinalis:0.0023310023

31):0.001762416118):0.01121794872):0.006890903506):0.0181807651,((Corydalis_sheareri:0.01
637476482,(Viola_moupinensis:0.01098432193,((Allium_macranthum:0.002622377622,Henckeli
a_pumila:0.002622377622):0.004308673278,(Tiarella_polyphylla:0.003460530456,(Lysionotus_s
erratus:0.001566591062,Primula_denticulata:0.001566591062):0.001893939394):0.00347052044
4):0.004053271027):0.00539044289):0.01686989817,((Viola_delavayi:0.01073309406,(Primula_
conspersa:0.006169018529,(Swertia_ciliata:0.003260531018,(Pedicularis_davidii:0.00101981352,
(Corydalis_radicans:0,Fritillaria_cirrhosa:0):0.00101981352):0.002240717498):0.002908487511):
0.00456407553):0.01189053516,((Viola_arcuata:0.008021143827,(Pedicularis_macrosiphon:0,Pri
mula_malacoides:0):0.008021143827):0.00980661177,((Triglochin_maritima:0.003003588777,(E
pilobium_blinii:0.002290854909,Gentianopsis_grandis:0.002290854909):0.0007127338685):0.00
7571319968,(Corydalis_bulleyana:0.005064761261,Pedicularis_longipetiolata:0.005064761261):
0.005510147484):0.007252846852):0.004795873626):0.01062103376):0.007138372781):0.0193
8827556):0.00286178681):0.02963488149):0.02506898218):0.0390308955):0.08436043433,((Lo
belia_alsinoides:0.09743905191,Oldenlandia_corymbosa:0.09743905191):0.1315819271,((((((Sa
xifraga_umbellulata:0.02294252756,(Corydalis_hendersonii:0.0171291861,Pedicularis_cryptanth
a:0.0171291861):0.005813341457):0.01280869417,((Juncus_minimus:0.006230262426,Pedicular
is_lyrata:0.006230262426):0.01811550881,(((Saxifraga_lychnitis:0.001311188811,Saxifraga_ores
bia:0.001311188811):0.003205128205,(Pedicularis_longiflora:0.002089764478,Saxifraga_tibetica:
0.002089764478):0.002426552538):0.01017799323,(Corydalis_chrysosphaera:0.007679382255,S
axifraga_hirculus:0.007679382255):0.007014927991):0.009651460989):0.01140545049):0.02322
969528,(Hypericum_japonicum:0.03968885018,((Chrysosplenium_davidianum:0.01547892861,(J
uncus_leucanthus:0.007138694639,Viola_biflora:0.007138694639):0.008340233971):0.01607506
621,(((Hypericum_wightianum:0.003496503497,Xyris_pauciflora:0.003496503497):0.006845187
651,(Saxifraga_maxionggouensis:0.008941042055,(Juncus_sikkimensis:0.001365284479,Saxifra
ga_hookeri:0.001365284479):0.007575757576):0.001400649092):0.01148953471,(((Juncus_prze
walskii:0,Juncus_thomsonii:0):0.004967544324,(Saxifraga_ciliatopetala:0.004336323004,Saxifra
ga_sinomontana:0.004336323004):0.0006312213192):0.006515796634,((Corydalis_cavei:0,Genti
ana_purdomii:0):0.006835500666,(Mimulus_tenellus:0.005983248588,(Oxalis_corniculata:0.002
769797069,Saxifraga_heleonastes:0.002769797069):0.003213451519):0.0008522520771):0.0046
47840292):0.0103478849):0.009722768968):0.008134855359):0.01929206682):0.0238308178,((
(Juncus_potaninii:0.02263933361,Pedicularis_latirostris:0.02263933361):0.008665138279,((Saxif
raga_gemmigera:0.01027801392,(Corydalis_conspersa:0,Corydalis_giraldii:0):0.01027801392):0.
01274532403,(Hypericum_gramineum:0.01045520412,(Corydalis_hamata:0.002442383611,Utric
ularia_scandens:0.002442383611):0.008012820513):0.01256813383):0.00828113394):0.0339769
8692,((Frigidorchis_humidicola:0.01078278253,Viola_confertifolia:0.01078278253):0.03409090
909,(Saxifraga_diapensia:0.01581215623,(Pedicularis_muscoides:0.01392405789,(Saxifraga_elli
otii:0.005218772193,Saxifraga_parva:0.005218772193):0.008705285701):0.001888098338):0.02
906153539):0.02040776719):0.017530276):0.03693432399,((((Dactylicapnos_scandens:0.014860
13986,Dactylicapnos_torulosa:0.01486013986):0.02411381117,(((Ludwigia_octovalvis:0.003907
583409,Meconopsis_chelidoniifolia:0.003907583409):0.01567230964,(Homonoia_riparia:0.0136
9463869,Polygala_fallax:0.01369463869):0.005885254357):0.01111584523,(Corydalis_raddeana:
0.02026818389,Swertia_bimaculata:0.02026818389):0.0104275544):0.008278212752):0.0344660
1594,(((Salix_denticulata:0.009761072261,Salix_wilhelmsiana:0.009761072261):0.01854170757,
(Salix_sclerophylloides:0.02358302667,((Salix_cyanolimnea:0.005328486798,Salix_resecta:0.00

5328486798):0.007575757576,(Salix_daliensis:0.006202612672,Salix_eriostachya:0.0062026126
72):0.006701631702):0.01067878229):0.004719753163):0.02696637882,(Salix_variegata:0.0315
5226302,(Hypericum_lagarocladum:0.01497984448,(Hypericum_acmosepalum:0.004079254079,
Hypericum_beanii:0.004079254079):0.0109005904):0.01657241854):0.02371689563):0.0181708
0832):0.01339905581,(((Meconopsis_paniculata:0.02118245715,(Ligularia_dictyoneura:0.009442
366153,(Cremanthodium_brunneopilosum:0.002622377622,Lycoris_aurea:0.002622377622):0.00
681998853):0.011740091):0.02089394163,(((Primula_alpicola:0.003496503497,Primula_sikkime
nsis:0.003496503497):0.009429549575,(Impatiens_chimiliensis:0.007208261822,(Impatiens_dre
panophora:0.00101981352,Lysimachia_biflora:0.00101981352):0.006188448302):0.00571779125)
:0.01758041341,((Senecio_arachnanthus:0.01346101193,(Primula_florindae:0.006390114442,(Pri
mula_bathangensis:0.002331002331,Primula_bulleyana:0.002331002331):0.004059112111):0.00
707089749):0.01267482517,((Cremanthodium_ellisii:0.01099614061,(Lysimachia_paridiformis:0.
003606218102,((Impatiens_tortisepala:0,Primula_beesiana:0):0.002983320097,(Iris_halophila:0.0
002913752914,Primula_agleniana:0.0002913752914):0.002691944806):0.0006228980051):0.007
389922508):0.01149750533,((Primula_helodoxa:0.00622442137,(Corydalis_ophiocarpa:0,Impati
ens_bicornuta:0):0.00622442137):0.01075323603,((Impatiens_cyathiflora:0.004627698324,(Impa
tiens_clavigera:0.002296695993,Impatiens_rectangula:0.002296695993):0.002331002331):0.003
754387978,(Primula_chungensis:0.005022947137,(Impatiens_dicentra:0.001562416708,Impatien
s_scutisepala:0.001562416708):0.003460530429):0.003359139165):0.008595571096):0.0055159
8854):0.00364219117):0.004370629371):0.01156993231):0.03302674059,(((((Impatiens_nyiman
a:0.003787878788,(Crepis_multicaulis:0.0004370629371,(Allium_atrosanguineum:0,Corydalis_g
racillima:0):0.0004370629371):0.003350815851):0.006202612672,((Hylomecon_japonica:0,Pote
ntilla_inclinata:0):0.005314322428,(Corydalis_balansae:0.0005827505828,Impatiens_holocentra:
0.0005827505828):0.004731571845):0.004676169032):0.005667653812,(Lindenbergia_muraria:
0.01314130787,(Lysimachia_congestiflora:0.001708104326,Saxifraga_nigroglandulifera:0.00170
8104326):0.01143320355):0.002516837399):0.02640449863,((Viola_urophylla:0.02162690111,((
(Corydalis_bimaculata:0,Impatiens_xanthina:0):0.005244755245,((Impatiens_soulieana:0,Juncus_
libanoticus:0):0.0015252497,(Lysimachia_phyllocephala:0.00101981352,Primula_prenantha:0.00
101981352):0.0005054361802):0.003719505545):0.009373434511,((Pedicularis_chinensis:0.002
442383611,(Corydalis_ellipticarpa:0.001165501166,(Impatiens_gracilipes:0.0001456876457,Imp
atiens_principis:0.0001456876457):0.00101981352):0.001276882445):0.006847319347,(Iris_farr
eri:0.004587550901,Primula_orbicularis:0.004587550901):0.004702152057):0.005328486798):0.
007008711353):0.01102217736,(((Hypericum_ascyron:0.00807832567,(Impatiens_ceratophora:0.
002436542582,Juncus_giganteus:0.002436542582):0.005641783088):0.00665383998,(((Impatien
s_polyceras:0.001560749978,(Impatiens_desmantha:0.0007284382284,Lysimachia_rubiginosa:0.
0007284382284):0.00083231175):0.003330673883,((Ludwigia_prostrata:0.001165501166,(Impat
iens_notolopha:0,Lysimachia_capillipes:0):0.001165501166):0.002331002331,(Lysimachia_hems
leyi:0.002097902098,(Mimulus_bracteosus:0,Salix_etosia:0):0.002097902098):0.001398601399):
0.001394920365):0.005897073011,((Iris_wilsonii:0.00203962704,(Impatiens_lasiophyton:0.0007
284382284,(Impatiens_radiata:0.0001456876457,Impatiens_vittata:0.0001456876457):0.0005827
505828):0.001311188811):0.004308673278,(Primula_firmipes:0.002691944806,(Impatiens_corch
orifolia:0.001271041389,Iris_forrestii:0.001271041389):0.001420903417):0.003656355512):0.00
4440196554):0.003943668778):0.009761072261,(((Impatiens_mengtszeana:0.003711758325,(Im
patiens_scabrida:0.001853791972,Ligularia_hookeri:0.001853791972):0.001857966354):0.00529

4892655,((Allium_rude:0.001456876457,Impatiens_noli.tangere:0.001456876457):0.0049190736
15,(Impatiens_siculifer:0.003350815851,(Impatiens_potaninii:0.00101981352,(Impatiens_infirma:
0.0002913752914,Impatiens_racemosa:0.0002913752914):0.0007284382284):0.002331002331):
0.003025134221):0.002630700909):0.007623516089,(Lysimachia_hemsleyana:0.01078849687,(
Mimulus_szechuanensis:0.003364980221,(Corydalis_adunca:0.002240717498,Xyris_capensis:0.0
02240717498):0.001124262723):0.007423516651):0.005841670198):0.007863070841):0.008155
840557):0.009413565438):0.008680240924,(((((Primula_melanodonta:0.003688325916,(Corydal
is_wuzhengyiana:0.001048951049,Impatiens_crassicaudex:0.001048951049):0.002639374867):0.
002447552448,(Fritillaria_davidii:0.004369089381,Lysimachia_microcarpa:0.004369089381):0.0
01766788982):0.009670300243,(Impatiens_muliensis:0.00846404782,(Impatiens_ruiliensis:0.003
496503497,(Salix_myrtillacea:0.0003496503497,(Impatiens_latebracteata:0,Juncus_grisebachii:0):
0.0003496503497):0.003146853147):0.004967544324):0.007342130786):0.009830639444,(Prim
ula_calthifolia:0.01674987166,((Impatiens_divaricata:0.004044947741,(Lysimachia_omeiensis:0.
001223776224,Salix_rehderiana:0.001223776224):0.002821171517):0.003359139137,((Impatien
s_barbata:0,Primula_serratifolia:0):0.005018885111,(Lysimachia_foenum.graecum:0.0009656140
833,Primula_chrysochlora:0.0009656140833):0.004053271027):0.002385201767):0.0093457847
85):0.008886946387):0.018502331,((((Hypericum_elodeoides:0.001610887417,Lysimachia_dry
marifolia:0.001610887417):0.004336323004,(Impatiens_alpicola:0.004891423861,(Hypericum_
monanthemum:0.002003653999,Saxifraga_tigrina:0.002003653999):0.002887769862):0.0010557
8656):0.009014262099,(Pedicularis_gyrorhyncha:0.007910717026,(Impatiens_falcifer:0.0030176
26435,Juncus_sphacelatus:0.003017626435):0.004893090591):0.007050755494):0.01442307692,
((Saxifraga_moorcroftiana:0.005339392969,(Corydalis_racemosa:0.002636541993,Hypericum_p
erforatum:0.002636541993):0.002702850976):0.01394912476,(Pedicularis_pseudocurvituba:0.01
486038441,((Mimulus_bodinieri:0.001005893694,Pedicularis_cranolopha:0.001005893694):0.00
8026984883,(Pedicularis_lasiophrys:0.005454744671,(Hypericum_reptans:0.002199479056,Pedi
cularis_plicata:0.002199479056):0.003255265616):0.003578133906):0.005827505828):0.004428
133327):0.01009603171):0.01475459961):0.006603735778):0.02436025454):0.01173588341):0.
03290703602):0.06986811384,((Salix_pseudowallichiana:0.0914524649,(Eriocaulon_leianthum:0.
06098116745,((Eriocaulon_kunmingense:0.01787522543,Eriocaulon_minusculum:0.0178752254
3):0.02572475719,(Eriocaulon_alpestre:0.02954860905,(Eriocaulon_nepalense:0.01952214452,E
riocaulon_sollyanum:0.01952214452):0.01002646453):0.01405137358):0.01738118482):0.03047
129745):0.09245259584,((((Juncus_triglumis:0.01477220072,Juncus_wallichianus:0.0147722007
2):0.03796251943,(Juncus_himalensis:0.02682069118,(Juncus_leptospermus:0.01396184362,Luz
ula_multiflora:0.01396184362):0.01285884755):0.02591402897):0.01822855685,((Cremastra_ap
pendiculata:0.03441989092,((Luzula_effusa:0.01119196569,Salix_saposhnikovii:0.01119196569):
0.01196816238,((Juncus_nepalicus:0.003219292575,Luzula_plumosa:0.003219292575):0.012211
77919,(Juncus_amplifolius:0.008996660992,Juncus_castaneus:0.008996660992):0.00643441076
9):0.007729056312):0.01125976284):0.01836673671,((Salix_rhoophila:0.01056142339,(Salix_di
ssa:0.005869708633,(Salix_characta:0,Salix_fedtschenkoi:0):0.005869708633):0.004691714756):
0.02690785947,(Salix_sphaeronymphe:0.02070524679,(Salix_michelsonii:0.006224421342,(Sali
x_hypoleuca:0.002145167263,Salix_tenuijulis:0.002145167263):0.004079254079):0.0144808254
5):0.01676403606):0.01531734477):0.01817664937):0.0881276321,(((Chrysosplenium_lanugino
sum:0.003420383034,Parnassia_yunnanensis:0.003420383034):0.02932259335,(Juncus_heptopot
amicus:0.01519509928,(Ammannia_baccifera:0.01221939028,Juncus_bufonius:0.01221939028):

0.002975709006):0.0175478771):0.04434258526,(((Salix_oritrepha:0.01239761425,Salix_rosmar
inifolia:0.01239761425):0.03455318423,((((Salix_rockii:0.0008741258741,Salix_taoensis:0.0008
741258741):0.00472027972,(Salix_erioclada:0.001748251748,Salix_luctuosa:0.001748251748):0.
003846153846):0.007998129586,(Salix_haoana:0.008493467581,Salix_shihtsuanensis:0.0084934
67581):0.005099067599):0.01531136717,(Salix_cheilophila:0.0143829295,(Salix_delavayana:0.0
04967544324,Salix_paratetradenia:0.004967544324):0.009415385177):0.01452097285):0.01804
689613):0.01378669359,(((Excoecaria_acerifolia:0.005869708633,(Asarum_cardiophyllum:0,Scr
ophularia_elatior:0):0.005869708633):0.02399205117,((Codonopsis_benthamii:0.01214982312,(
Swertia_cincta:0.005765549735,Swertia_nervosa:0.005765549735):0.006384273386):0.0074560
52954,(Gentiana_macrophylla:0.01300978463,Gentiana_stylophora:0.01300978463):0.00659609
145):0.01025588373):0.02271722411,(((Gentiana_straminea:0.01556843609,(Swertia_tetraptera:
0.008312519118,(Swertia_przewalskii:0.005155046066,(Actinostemma_tenerum:0,Peristylus_elis
abethae:0):0.005155046066):0.003157473053):0.007255916974):0.01575091239,(Swertia_tibetic
a:0.02250019924,(Juncus_alatus:0.01418183904,((Juncus_prismatocarpus:0.001748251748,Swert
ia_kouitchensis:0.001748251748):0.006092898067,(Juncus_compressus:0.003893419011,Peristyl
us_forceps:0.003893419011):0.003947730804):0.006340689227):0.008318360202):0.008819149
239):0.01373339488,((Calanthe_davidii:0.01818181818,((Epipactis_helleborine:0.00101981352,
Swertia_erythrosticta:0.00101981352):0.006847319347,(Pedicularis_pseudoingens:0.0030504993
67,(Juncus_inflexus:0,Salix_disperma:0):0.003050499367):0.0048166335):0.01031468531):0.018
88059235,((Corydalis_auriculata:0.005163398667,(Juncus_setchuensis:0,Salix_driophila:0):0.005
163398667):0.01339406562,((Juncus_diastrophanthus:0.002630700936,Juncus_tenuis:0.0026307
00936):0.01130168027,((Fritillaria_sichuanica:0.002345166701,Peristylus_tentaculatus:0.002345
166701):0.009491360103,(Swertia_wolfgangiana:0.00274614427,(Epipactis_humilior:0.0010198
1352,Herminium_monorchis:0.00101981352):0.00172633075):0.009090382534):0.00209585440
4):0.004625083083):0.01850494624):0.007990332828):0.007526240549):0.008158508159):0.01
634806957):0.08200534745):0.02481415165):0.005709111901):0.03940680638):0.01170731261):
0.0926050417):0.06060606061):0.1060606061,(((((Sanicula_lamelligera:0.03027704725,(Sanicul
a_caerulescens:0.01507539469,Sanicula_orthacantha:0.01507539469):0.01520165256):0.116488
1605,(Centella_asiatica:0.04807692308,Tongoloa_silaifolia:0.04807692308):0.09868828463):0.1
280930366,(Tacca_chantrieri:0.183598199,((Berberis_sanguinea:0.03287684538,(Lobelia_numm
ularia:0.01853021077,Vaccinium_vitis.idaea:0.01853021077):0.0143466346):0.06348489545,((M
usa_rubra:0.02054195804,Sauromatum_giganteum:0.02054195804):0.03294036307,((Dysosma_
delavayi:0.00447628195,Sinopodophyllum_hexandrum:0.00447628195):0.04068268065,(Dysos
ma_versipellis:0.02395052242,Polygonatum_kingianum:0.02395052242):0.02120844018):0.0083
23358512):0.04287941971):0.08723645822):0.09126004528):0.07661562938,(((((((Fimbristylis_
stolonifera:0.01538260105,((Bolboschoenus_planiculmis:0.001566591035,Carex_cespitosa:0.001
566591035):0.01029185677,(((Carex_lancisquamata:0.001456876457,Carex_obscuriceps:0.0014
56876457):0.002436542554,(Carex_hongyuanensis:0.001748251748,(Carex_arcatica:0,Carex_me
yeriana:0):0.001748251748):0.002145167263):0.002456548009,(Carex_hancockiana:0.00451187
9408,(Carex_haematostoma:0,Carex_scabrirostris:0):0.004511879408):0.001838087612):0.00550
8480782):0.00352415325):0.01964668304,((Carex_makuensis:0.005881368651,(Carex_earistata:
0.003672059872,Pycreus_delavayi:0.003672059872):0.002209308778):0.01418729772,((Carex_c
aespititia:0.004164624567,Trichophorum_pumilum:0.004164624567):0.008101268267,(Carex_m
aquensis:0.006667325335,Carex_satakeana:0.006667325335):0.005598567498):0.007802773533)

:0.01496061772):0.009672734682,(((Fimbristylis_nigrobrunnea:0.01269660064,(Carex_duriuscul
a:0.005897073011,(Carex_karlongensis:0.003857445971,Kobresia_macrantha:0.003857445971):
0.00203962704):0.006799527625):0.01350880363,(((Carex_coriophora:0.001311188811,Carex_i
nanis:0.001311188811):0.006599528187,((Eleocharis_quinqueflora:0,(Blysmus_compressus:0,Ca
rex_alba:0):0):0.004308608179,(Carex_curaica:0.001347226951,Carex_shandanica:0.001347226
951):0.002961381228):0.003602108819):0.004821856678,((Kobresia_filicina:0.001456876457,(
Carex_enervis:0,Eleocharis_erhaiensis:0):0.001456876457):0.007048122793,((Carex_capillacea:
0,Carex_capillaris:0):0.005107390913,(Carex_amgunensis:0.001893939394,Carex_parva:0.00189
3939394):0.003213451519):0.003397608337):0.004227574426):0.01347283059):0.00906478492
3,(((Carex_laeta:0.006971085995,(Blysmus_sinocompressus:0.002600456624,(Carex_przewalski
i:0.001748251748,Cyperus_fuscus:0.001748251748):0.0008522048759):0.004370629371):0.008
515388579,((Cyperus_amuricus:0.002913752914,Fimbristylis_dichotoma:0.002913752914):0.00
5827505828,(Kobresia_tibetica:0.005058920177,(Kobresia_capillifolia:0.0017050841,Kobresia_
graminifolia:0.0017050841):0.003353836077):0.003682338564):0.006745215833):0.0123976142
5,((Kobresia_duthiei:0.005785691731,Kobresia_hohxilensis:0.005785691731):0.01015201015,((
Kobresia_cuneata:0.002199479056,Kobresia_laxa:0.002199479056):0.004370629371,(Carex_dre
panorhyncha:0.005767216437,(Carex_atrata:0.004044947741,(Carex_gentilis:0,Fimbristylis_quin
quangularis:0):0.004044947741):0.001722268696):0.0008028919891):0.009367593455):0.01194
638695):0.007386100358):0.009431829587):0.04571841193,(((Carex_cruciata:0.00521877222,C
arex_filicina:0.00521877222):0.02198046257,(Carex_baccans:0.02540739888,Scleria_terrestris:0.
02540739888):0.001791835907):0.03130867945,(((Carex_olivacea:0.008485921617,(Carex_wui:
0.003059440559,(Carex_insignis:0,Carex_longispiculata:0):0.003059440559):0.005426481058):0.
01282632698,(Fimbristylis_complanata:0.01085877628,(Cyperus_serotinus:0.003310668456,Ko
bresia_kansuensis:0.003310668456):0.007548107822):0.01045347232):0.02201650073,(Cladium
_mariscus:0.02728296634,((Scirpus_lushanensis:0,Scirpus_wichurae:0):0.01951793696,(Carex_p
amirensis:0.007527965854,Carex_thibetica:0.007527965854):0.01198997111):0.00776502938):0.
01604578299):0.01517916491):0.03191251647):0.04492837053,((Bulbostylis_densa:0.06241206
088,((Kobresia_filifolia:0.01568991075,Kobresia_pusilla:0.01568991075):0.02700163967,(((Fim
bristylis_littoralis:0.003364980221,Pycreus_flavidus:0.003364980221):0.01307758177,(Carex_br
unnea:0.006932717603,Kobresia_uncinioides:0.006932717603):0.009509844392):0.0131278224
9,((Fimbristylis_bisumbellata:0.004148821262,Kobresia_littledalei:0.004148821262):0.01236330
789,((Kobresia_schoenoides:0.006334135948,(Kobresia_vidua:0.002331002331,(Kobresia_myos
uroides:0,Kobresia_setschwanensis:0):0.002331002331):0.004003133617):0.005474886667,(Kob
resia_robusta:0.007250075918,(Kobresia_royleana:0.005107390913,((Carex_cardiolepis:0,Eleoch
aris_yunnanensis:0):0.004198958673,(Carex_lanceolata:0.002005320673,Kobresia_tunicata:0.00
2005320673):0.002193637999):0.0009084322403):0.002142685005):0.004558946697):0.004703
106535):0.01305825533):0.01312116593):0.01972051047):0.04243027387,(Carex_sagaensis:0.0
676961927,((Carex_tangulashanensis:0.02032014993,Kobresia_pygmaea:0.02032014993):0.0228
5661917,(((Carex_aridula:0.003470520472,Cyperus_cuspidatus:0.003470520472):0.0085194506
33,(Carex_pseudofoetida:0.005404607261,Fimbristylis_aestivalis:0.005404607261):0.006585363
845):0.01622618028,((Carex_montis.everesti:0,(Carex_ivanoviae:0,Carex_microglochin:0):0):0.0
1496061772,(Eleocharis_uniglumis:0.005869708633,(Blysmus_rufus:0,Cyperus_squarrosus:0):0.
005869708633):0.009090909091):0.01325553366):0.01496061772):0.02451942359):0.03714614
206):0.03050646648):0.08497948159,(((Eleocharis_yokoscensis:0.04072829167,(Mosla_dianther

a:0.02249364597,Pogostemon_auricularius:0.02249364597):0.0182346457):0.05285948394,((Aju
ga_forrestii:0.01808697843,(Ajuga_ciliata:0.009214359818,Scutellaria_discolor:0.009214359818)
:0.008872618609):0.03273963796,(((Salvia_plebeia:0.00552837388,(Carex_muliensis:0.0030052
41123,Stachys_oblongifolia:0.003005241123):0.002523132757):0.01531250544,(Carex_melinacr
a:0.009163946644,Phlomoides_tibetica:0.009163946644):0.01167693268):0.01436762688,(Prune
lla_vulgaris:0.02246766291,(Pogostemon_linearis:0.02025058275,(Elsholtzia_densa:0.01257927
497,(Scleria_hookeriana:0.006967023969,Trigonotis_omeiensis:0.006967023969):0.0056122509
99):0.007671307783):0.002217080162):0.01274084329):0.01561811018):0.04276115922):0.098
45563684,((Carex_kansuensis:0.04766870422,(Carex_ensifolia:0.02006866637,Eleocharis_fenni
ca:0.02006866637):0.02760003785):0.1153463408,((Microula_tibetica:0.05161280314,(Microula
_leiocarpa:0.03034661443,Microula_younghusbandii:0.03034661443):0.02126618871):0.028975
81527,((((Eritrichium_acicularum:0.008304195804,Eritrichium_fruticulosum:0.008304195804):0.
01200413548,(Eritrichium_tangkulaense:0.01034382284,Trigonotis_rockii:0.01034382284):0.009
964508436):0.01708903868,((Myosotis_caespitosa:0.01187261217,(Microula_floribunda:0.00734
8684065,(Microula_stenophylla:0.0008741258741,Trigonotis_peduncularis:0.0008741258741):0.
006474558191):0.004523928107):0.01280634845,(Scutellaria_tenax:0.0173662496,(Trigonotis_h
eliotropifolia:0.01143320355,(Trigonotis_gracilipes:0.008860963335,(Glechoma_longituba:0.001
893939394,Microula_trichocarpa:0.001893939394):0.006967023941):0.002572240212):0.005933
046051):0.007312711025):0.01271840933):0.02239586237,((Amethystea_caerulea:0.021631338
74,(Hackelia_difformis:0.01844037491,Mentha_canadensis:0.01844037491):0.003190963835):0.
02606206233,((Microula_sikkimensis:0.01088653836,Trigonotis_cavaleriei:0.01088653836):0.02
168908725,(Scutellaria_barbata:0.01257856272,(Ajuga_nipponensis:0.00101981352,Microula_di
ffusa:0.00101981352):0.0115587492):0.01999706289):0.01511777547):0.01209983125):0.02079
538609):0.08242642658):0.02902836745):0.02828487038):0.09785353535,((((((Veronica_undula
ta:0.01806526807,(Swertia_macrosperma:0.01493803036,(Corydalis_elata:0.002231559667,Flos
copa_scandens:0.002231559667):0.01270647069):0.003127237708):0.04153514339,((Iris_confus
a:0.03319080019,(Corydalis_temulifolia:0.009920924277,(Commelina_paludosa:0.00419895870
1,Strobilanthes_inflata:0.004198958701):0.005721965576):0.02326987592):0.02069760247,(((Ps
eudolysimachion_linariifolium:0.01193456829,(Corydalis_flexuosa:0.007118552671,Cyanotis_ar
achnoidea:0.007118552671):0.004816015622):0.01374406388,(((Gentianopsis_barbata:0.005370
300922,Halenia_elliptica:0.005370300922):0.00605108397,(Commelina_communis:0.006196771
616,(Commelina_benghalensis:0.0001748251748,Commelina_maculata:0.0001748251748):0.006
021946441):0.005224613277):0.006390114414,(Swertia_wardii:0.01156707251,((Corydalis_bals
amiflora:0.001610887417,Swertia_bifolia:0.001610887417):0.005078925603,(Gentiana_dahurica:
0.004384793741,Gentiana_siphonantha:0.004384793741):0.002305019279):0.004877259491):0.0
06244426796):0.007867132867):0.01511759052,((Primula_moupinensis:0.01742012754,(Iris_bul
leyana:0.0101981352,(Cyanotis_cristata:0.003642191142,Gentianella_turkestanorum:0.00364219
1142):0.006555944056):0.007221992342):0.0091246889,((Iris_clarkei:0.003846153846,(Iris_jap
onica:0.001398601399,Iris_laevigata:0.001398601399):0.002447552448):0.01440838602,(Lobeli
a_nicotianifolia:0.01067878229,(Primula_deflexa:0.006154919258,Veronica_anagalloides:0.0061
54919258):0.004523863036):0.007575757576):0.008290276571):0.01425140625):0.0130921799
7):0.005712008797):0.0663406489,(((Gentiana_crenulatotruncata:0.01665197574,(Gentiana_clar
kei:0.008639155227,Gentiana_decorata:0.008639155227):0.008012820513):0.02818551277,(((G
entiana_tatsienensis:0.003642191142,Gentiana_tricolor:0.003642191142):0.01267482517,(Gentia

na_riparia:0.0132457571,(Metaeritrichium_microuloides:0.00895651357,(Gentiana_grumii:0.003
711758325,Primula_oxygraphidifolia:0.003711758325):0.005244755245):0.004289243533):0.00
3071259213):0.01388391047,(Gentiana_ninglangensis:0.02179951407,((Comastoma_disepalum:
0.006667325363,Gentiana_crassuloides:0.006667325363):0.009587734861,(Gentiana_aquatica:0.
01119196567,Lomatogonium_brachyantherum:0.01119196567):0.005063094559):0.00554445385)
:0.008401412713):0.01463656172):0.03376934321,((((Corydalis_zadoiensis:0.01631701632,(Co
mastoma_tenellum:0.01166917603,(Gentiana_piasezkii:0.006771198885,Lomatogoniopsis_galeif
ormis:0.006771198885):0.00489797714):0.004647840292):0.0136419604,((Gentiana_grata:0.006
092898095,(Comastoma_polycladum:0.004224941725,Cyananthus_hookeri:0.004224941725):0.0
0186795637):0.01522108233,(Swertia_diluta:0.01236330789,(Lomatogonium_forrestii:0.003607
884776,Lomatogonium_rotatum:0.003607884776):0.008755423111):0.008950672542):0.008644
996283):0.0212448471,(((Comastoma_stellariifolium:0.003059440559,Veronica_pusilla:0.00305
9440559):0.01346101193,(Gentiana_aperta:0.003933566434,Gentiana_pseudoaquatica:0.0039335
66434):0.01258688606):0.02464508813,((Lomatogonium_longifolium:0.01106044239,Swertia_fr
anchetiana:0.01106044239):0.00812463567,((Comastoma_falcatum:0.007645324759,(Lomatogon
ium_macranthum:0.003857445971,(Corydalis_pseudofilisecta:0.002185314685,(Lomatogonium_
carinthiacum:0.0008741258741,Lomatogonium_gamosepalum:0.0008741258741):0.00131118881
1):0.001672131286):0.003787878788):0.01110269036,((Comastoma_pulmonarium:0.001893939
394,Veronica_serpyllifolia:0.001893939394):0.007515902035,(Swertia_davidii:0.00812463567,(
Corydalis_rorida:0.004370629371,Gentianopsis_paludosa:0.004370629371):0.003754006299):0.
001285205759):0.009338173694):0.0004370629371):0.02198046257):0.01003828318):0.008785
179877,((((Gentiana_nubigena:0.00238406093,Primula_stenocalyx:0.00238406093):0.010556156
51,(Primula_amethystina:0.002689023645,Swertia_graciliflora:0.002689023645):0.0102511938):
0.0242495088,(Corydalis_ludlowii:0.01829468726,Gentiana_sutchuenensis:0.01829468726):0.01
889503898):0.01761227072,(Gentiana_yokusai:0.03762501915,(((Gentiana_aristata:0.002345166
701,Gentiana_pudica:0.002345166701):0.01140722052,(Corydalis_longibracteata:0.00623858574,
(Comastoma_pedunculatum:0.002768065268,Gentiana_picta:0.002768065268):0.003470520472):
0.007513801483):0.01223776224,(((Gentiana_exigua:0.0001456876457,Gentiana_flexicaulis:0.0
001456876457):0.01050367486,(Gentiana_ludingensis:0.004821856678,Gentiana_pedicellata:0.0
04821856678):0.005827505828):0.01372405848,((Gentiana_panthaica:0.009615384615,Gentiana
_veitchiorum:0.009615384615):0.009178321678,(Gentiana_mairei:0.01275855673,Gentiana_stip
itata:0.01275855673):0.006035149565):0.005579714695):0.001616728473):0.01163486969):0.01
717697782):0.005187006716):0.01861782803):0.04733422864):0.06627448524,((Oxytropis_mic
rophylla:0.0704826299,(Astragalus_laxmannii:0.01060921511,Crotalaria_sessiliflora:0.01060921
511):0.05987341479):0.0721849518,(Lancea_tibetica:0.1250695672,(Delphinium_delavayi:0.081
75177387,Gentiana_qiujiangensis:0.08175177387):0.04331779332):0.01759801451):0.04954796
39):0.09135954739,(((((((Lindernia_hyssopoides:0.01078088578,Rotala_rotundifolia:0.01078088
578):0.01752610161,(Corydalis_pingwuensis:0.0131814553,(Corydalis_ternatifolia:0.000524475
5245,Pedicularis_densispica:0.0005244755245):0.01265697977):0.0151255321):0.01939062127,(
Lobelia_chinensis:0.03412918444,(Epilobium_amurense:0.02255670327,Murdannia_loriformis:0.
02255670327):0.01157248117):0.01356842423):0.05580755425,((((Myricaria_rosea:0.01912524
229,(Corydalis_edulis:0,Microcarpaea_minima:0):0.01912524229):0.01863699547,((Pedicularis_
gruina:0.01441125824,(Lysimachia_maritima:0.01295438178,Murdannia_triquetra:0.0129543817
8):0.001456876457):0.009178321678,(Pedicularis_roylei:0.01547465592,((Pedicularis_kansuensi

s:0.003059440559,Pedicularis_microchila:0.003059440559):0.005815687146,((Pedicularis_cheila
nthifolia:0,Salix_brachista:0):0.007152859009,(Allium_polyrhizum:0.002185314685,Epilobium_
sikkimense:0.002185314685):0.004967544324):0.001722268696):0.006599528215):0.008114924)
:0.01417265784):0.02055219708,(((Schnabelia_terniflora:0.007721445221,Swertia_mussotii:0.00
7721445221):0.0154864746,(((Pedicularis_rhynchotricha:0.002097902098,(Pedicularis_axillaris:
0,Scrophularia_incisa:0):0.002097902098):0.009137647727,((Pedicularis_megalantha:0.0024365
42554,Saxifraga_pardanthina:0.002436542554):0.005815687173,(Impatiens_chinensis:0.0034705
20472,(Epilobium_pyrricholophum:0.002339325645,Pedicularis_resupinata:0.002339325645):0.0
01131194827):0.004781709256):0.002983320097):0.01022411825,((Epilobium_platystigmatosu
m:0.003642191142,Epilobium_royleanum:0.003642191142):0.007950864421,(Juncus_articulatus:
0.007735609592,(Pedicularis_oliveriana:0.002199479056,Pedicularis_pseudomelampyriflora:0.0
02199479056):0.005536130536):0.003857445971):0.009866612512):0.001748251748):0.021014
96078,((Pedicularis_muscicola:0.01369463869,(Pedicularis_siphonantha:0.01159305556,(Aletris
_pauciflora:0.007076738546,Primula_gemmifera:0.007076738546):0.004516317016):0.0021015
83132):0.01462651307,(((Gymnadenia_conopsea:0.0005827505828,Primula_pseudodenticulata:0.
0005827505828):0.006813257526,(Asarum_himalaicum:0.004662249207,Sarcopyramis_napalen
sis:0.004662249207):0.002733758902):0.01064352145,(Pedicularis_przewalskii:0.009964508408,
(Impatiens_delavayi:0.003205128205,(Pedicularis_spicata:0.0008741258741,(Epilobium_wallichi
anum:0.0002913752914,Pedicularis_flexuosa:0.0002913752914):0.0005827505828):0.00233100
2331):0.006759380203):0.00807502115):0.01028162221):0.01590172884):0.01409155424):0.01
838420127,(((Gentiana_choanantha:0.002476689977,Utricularia_striatula:0.002476689977):0.04
449248044,(((Primula_concinna:0.002185314685,Primula_fasciculata:0.002185314685):0.01245
301707,(Primula_pumilio:0.004891423861,Primula_walshii:0.004891423861):0.009746907891):
0.0142655706,(Primula_tibetica:0.0203962704,(Gentiana_syringea:0.00642442078,Utricularia_sa
lwinensis:0.00642442078):0.01397184962):0.008507631951):0.01806526807):0.02272779928,((
Pedicularis_chenocephala:0.02972789081,(Spergularia_diandra:0.0278003573,((Pedicularis_verti
cillata:0.006625511239,(Lysimachia_pumila:0,Pedicularis_kialensis:0):0.006625511239):0.00544
0580329,(Pedicularis_sphaerantha:0.00999049146,(Salix_serpyllum:0.0008741258741,(Arenaria
_forrestii:0,Pedicularis_pheulpinii:0):0.0008741258741):0.009116365586):0.002075600108):0.01
573426573):0.001927533509):0.01977571811,((Pedicularis_habachanensis:0.01483415684,(Prim
ula_monticola:0.006453840569,Primula_vaginata:0.006453840569):0.008380316267):0.0212520
2431,(((Impatiens_laxiflora:0.002639374867,Pedicularis_diffusa:0.002639374867):0.0100343331
7,(Amitostigma_trifurcatum:0.005278749733,(Pedicularis_dolichantha:0.002639374867,Primula_
laciniata:0.002639374867):0.002639374867):0.0073949583):0.01196510519,(((Corydalis_mucro
nata:0.003169155165,Rotala_indica:0.003169155165):0.005560284895,((Epilobium_williamsii:0.
0007284382284,Pedicularis_globifera:0.0007284382284):0.004093418449,(Pedicularis_souliei:0.
001748251748,Pedicularis_szetschuanica:0.001748251748):0.00307360493):0.003907583382):0.
005474174444,(Epilobium_cylindricum:0.0121096757,(Saussurea_salicifolia:0.005032937152,(E
pilobium_brevifolium:0.002145167291,Saxifraga_lumpuensis:0.002145167291):0.002887769862)
:0.007076738546):0.002093938804):0.01043519872):0.01144736792):0.01341742777):0.020193
36078):0.007001666417):0.02680652681):0.01803507748,((Myricaria_paniculata:0.0441869407
7,(Myricaria_elegans:0.01359253518,(Myricaria_germanica:0.01013617911,Tamarix_hispida:0.0
1013617911):0.003456356074):0.03059440559):0.06153372745,((Rhododendron_galactinum:0.0
5254467754,((Epilobium_kermodei:0.009433723902,Rhododendron_chamaethomsonii:0.009433

723902):0.02270472647,((Rhododendron_primuliflorum:0.01143320355,(Rhododendron_tubifor
me:0.0001748251748,Salix_yadongensis:0.0001748251748):0.01125837837):0.01092657343,(Sa
lix_atopantha:0.01369463869,(Salix_sikkimensis:0,(Rhododendron_viscidifolium:0,Salix_pseudo
spissa:0):0):0.01369463869):0.008665138279):0.009778673396):0.02040622718):0.0182310977
1,(((Epilobium_pannosum:0.01162902863,Epilobium_parviflorum:0.01162902863):0.020891081
83,(((Allium_prattii:0.009221905837,(Epilobium_angustifolium:0.003881084809,Meconopsis_pi
nnatifolia:0.003881084809):0.005340821028):0.01031783979,((Pedicularis_superba:0.00378787
8788,Primula_wilsonii:0.003787878788):0.008361944306,((Dactylorhiza_umbrosa:0,Primula_poi
ssonii:0):0.005475841146,(Impatiens_arguta:0.003170821867,Primula_tangutica:0.00317082186
7):0.002305019279):0.006673981948):0.007389922536):0.007146305757,((Lobelia_davidii:0.00
5128487388,(Asarum_caudigerum:0,Epilobium_hirsutum:0):0.005128487388):0.01991141574,(A
llium_wallichii:0.01003674319,Asystasiella_neesiana:0.01003674319):0.01500315993):0.001646
148261):0.005834059079):0.02125202434,(((Primula_latisecta:0.005655835158,Primula_oreodo
xa:0.005655835158):0.01631280873,((Impatiens_chungtienensis:0.007224092894,(Impatiens_lec
omtei:0.00565583513,Primula_stenodonta:0.00565583513):0.001568257764):0.01031617309,((I
mpatiens_gongshanensis:0.004044947713,Spiranthes_sinensis:0.004044947713):0.005079741187,
(Impatiens_rostellata:0.00463602161,Pedicularis_furfuracea:0.00463602161):0.00448866729):0.0
08415577084):0.0044283779):0.021025146,((Primula_obconica:0.01529897283,(Primula_septem
loba:0.008056404644,Primula_waltonii:0.008056404644):0.007242568188):0.02122260455,(((Pr
imula_heucherifolia:0.001311188811,(Primula_aurantiaca:0,Primula_russeola:0):0.001311188811)
:0.01199971,(Impatiens_thomsonii:0.005443765942,(Amitostigma_gracile:0.002967075965,Gym
nadenia_orchidis:0.002967075965):0.002476689977):0.007867132867):0.01477662619,(((Impati
ens_pseudokingii:0.003642191142,Impatiens_uliginosa:0.003642191142):0.004334656303,(Impa
tiens_rubrostriata:0.007404086906,Impatiens_sulcata:0.007404086906):0.000572760539):0.0125
031545,(Primula_vialii:0.01858606256,(Primula_polyneura:0.01099614061,(Primula_calderiana:
0.008569588044,(Mazus_celsioides:0.003933566434,Pedicularis_vagans:0.003933566434):0.004
63602161):0.002426552566):0.007589921946):0.001893939394):0.007607523053):0.008434052
378):0.006472212503):0.01077834492):0.01700364045):0.03494489297):0.01581957218):0.069
359138,(((Braya_rosea:0.1205230596,(Cardamine_pulchella:0.03720809815,Cardamine_tangutor
um:0.03720809815):0.0833149614):0.01919479619,(Gonocarpus_micranthus:0.1331424359,(Sua
eda_przewalskii:0.03639592837,((Suaeda_heterophylla:0.01026770238,Suaeda_salsa:0.01026770
238):0.008354333215,(Suaeda_corniculata:0.01462651307,(Kalidium_foliatum:0.006384273358,
(Salicornia_europaea:0,Salsola_monoptera:0):0.006384273358):0.008242239712):0.00399552252
6):0.01777389277):0.09674650754):0.006575419836):0.04079176997,((Apocynum_pictum:0.09
221530133,(Rhodiola_tangutica:0.03408586002,((Sedum_obtrullatum:0.0091000424,Sedum_qua
drifidum:0.0091000424):0.0121502119,(Rhodiola_alterna:0.01450096742,Rhodiola_tibetica:0.01
450096742):0.006749286882):0.01283560571):0.05812944132):0.07647184962,((Rubus_pectina
rioides:0.04661506823,(Rubus_thibetanus:0.02609225295,((Rubus_coreanus:0.003656355512,Ru
bus_niveus:0.003656355512):0.0101981352,(Rubus_flosculosus:0.003947730804,Rubus_idaeops
is:0.003947730804):0.009906759907):0.01223776224):0.02052281528):0.1104312354,(((Carex_
obscura:0.01209968568,(Mentha_asiatica:0.005869708633,(Eleocharis_palustris:0,Teucrium_pilo
sum:0):0.005869708633):0.00622997705):0.02357541558,(Phlomoides_atropurpurea:0.02518559
077,(Carex_fargesii:0.01046359256,Phlomoides_tuberosa:0.01046359256):0.01472199821):0.01
048951049):0.04118524874,((Cyperus_pannonicus:0.0104275544,Isolepis_setacea:0.0104275544)

:0.03614813724,(((Carex_dielsiana:0.002490854347,Elsholtzia_pilosa:0.002490854347):0.01323
92038,(Carex_moorcroftii:0.007625182763,(Clinopodium_gracile:0.002879446548,(Clinopodium
_repens:0.001456876457,Lamium_amplexicaule:0.001456876457):0.001422570091):0.00474573
6215):0.00810487538):0.01062103376,((Phlomoides_dentosa:0.007765029353,Stachys_affinis:0.
007765029353):0.01265468321,(Pycreus_lijiangensis:0.01520094037,(Carex_orbicularis:0.01124
139088,(Carex_atrofusca:0.004496175048,Stachys_kouyangensis:0.004496175048):0.006745215
833):0.003959549486):0.005218772193):0.005931379349):0.02022459973):0.03028465837):0.0
8018595366):0.01164084728):0.01182247476):0.01038975269):0.08201879176,(((((Cardamine_
macrophylla:0.01622146611,Cardamine_multiflora:0.01622146611):0.0111300096,(Cardamine_g
riffithii:0.01752844736,Cardamine_multijuga:0.01752844736):0.009823028353):0.0257105928,((
Cardamine_franchetiana:0.01298036484,Cardamine_purpurascens:0.01298036484):0.019052545
28,(Aphragmus_hobsonii:0.02227183786,(Cardamine_gracilis:0.01234914352,(Cardamine_loxos
temonoides:0.003796202102,Cardamine_pratensis:0.003796202102):0.008552941415):0.009922
69434):0.009761072261):0.02102915839):0.09772464478,((Cardamine_parviflora:0.0200865231
4,(Cardamine_rockii:0.007653648073,Cardamine_simplex:0.007653648073):0.01243287507):0.0
4905315247,(Cardamine_circaeoides:0.0523018648,(((Cardamine_trifoliolata:0.01092657343,(C
ardamine_calcicola:0.003059440559,Cardamine_microzyga:0.003059440559):0.007867132867):
0.01995499987,(Cardamine_hirsuta:0.01841439186,Eutrema_deltoideum:0.01841439186):0.0124
6718144):0.01572010136,(Catolobus_pendulus:0.04070460168,((Cardamine_impatiens:0.011150
15157,Cardamine_paucifolia:0.01115015157):0.01909092264,((Cardamine_leucantha:0.0038574
45971,Cardamine_yunnanensis:0.003857445971):0.01735276424,(Arabidopsis_thaliana:0.011636
63972,Cardamine_lyrata:0.01163663972):0.009573570491):0.009030863998):0.01046352747):0.
005897072983):0.005700190143):0.01683781081):0.08164703768):0.0935708451,(((((Astragalu
s_tibetanus:0.01522940565,Vicia_bungei:0.01522940565):0.0158103862,(Oxytropis_falcata:0.02
200715781,(Phyllolobium_turgidocarpum:0.009906759907,(Astragalus_chagyabensis:0.0074040
86878,Astragalus_densiflorus:0.007404086878):0.002502673029):0.01210039791):0.009032634
033):0.01909599287,((Astragalus_arnoldii:0.02613583711,Hedysarum_tibeticum:0.02613583711)
:0.01812301659,((Astragalus_kuschakewiczii:0.009178321678,Hedysarum_tanguticum:0.009178
321678):0.01716515917,(Astragalus_nanjiangianus:0.02051597502,((Astragalus_licentianus:0.00
3350815851,Astragalus_minutidentatus:0.003350815851):0.009443713945,(Astragalus_nivalis:0.
006890903506,Astragalus_rigidulus:0.006890903506):0.00590362629):0.007721445221):0.0058
27505828):0.01791537286):0.005876931015):0.02913168808,((Vicia_cracca:0.03665133062,(De
smodium_heterocarpon:0.01188443085,Vicia_unijuga:0.01188443085):0.02476689977):0.013934
04791,(Glycine_soja:0.04328262431,((Astragalus_sulcatus:0.004148821262,Lathyrus_palustris:0.
004148821262):0.01558857809,(Astragalus_bhotanensis:0.01111584523,Astragalus_muliensis:0.
01111584523):0.00862155412):0.02354522496):0.007302754218):0.02868209427):0.079672770
26,(((Allium_schoenoprasum:0.04099597697,((Astragalus_hoantchy:0.002636541993,Hedysaru
m_alpinum:0.002636541993):0.02046583758,(Desmodium_elegans:0.01013617911,Lathyrus_die
lsianus:0.01013617911):0.01296620047):0.0178935974):0.0275743416,(Phyllolobium_heydei:0.
05770570124,((Astragalus_leansanicus:0.0187317502,(Astragalus_dahuricus:0.006529961004,K
ummerowia_striata:0.006529961004):0.0122017892):0.01171276016,(Astragalus_laspurensis:0.0
2008652314,(Astragalus_hotianensis:0.008743028776,Astragalus_strictus:0.008743028776):0.01
134349437):0.01035798721):0.02726119088):0.01086461733):0.08155986936,((Astragalus_yang
tzeanus:0.03512067939,(Astragalus_changduensis:0.01604741649,Phyllolobium_eutrichus:0.016

04741649):0.0190732629):0.1034122454,(((Aeschynomene_indica:0.01923076923,Lotus_tenuis:
0.01923076923):0.01271840933,((Astragalus_longilobus:0.003642191142,Medicago_lupulina:0.
003642191142):0.01739457837,(Crotalaria_ferruginea:0.008304195804,Lathyrus_pratensis:0.008
304195804):0.0127325737):0.01091240906):0.01763816191,(((Lotus_frondosus:0.00675105688
9,Oxytropis_kansuensis:0.006751056889):0.02107107585,(Crotalaria_prostrata:0.02157009488,(
Astragalus_mahoschanicus:0.009853127129,(Lotus_corniculatus:0.003656355512,Oxytropis_och
rocephala:0.003656355512):0.006196771616):0.01171696775):0.00625203786):0.0182729118,((
Astragalus_chilienshanensis:0.007978514147,Astragalus_handelii:0.007978514147):0.019897251
37,((Thermopsis_alpina:0.004636021638,Thermopsis_inflata:0.004636021638):0.01384032634,((
Astragalus_leucocephalus:0.002331002331,Astragalus_webbianus:0.002331002331):0.00999049
146,(Astragalus_lithophilus:0.004821856678,Astragalus_souliei:0.004821856678):0.0074996371
13):0.006154854187):0.009399417536):0.01821927903):0.003492295933):0.08894558433):0.01
159726313):0.008810055126):0.08541731533):0.02856061177):0.01065692284):0.02291233694,
((((((Setaria_plicata:0.02086916086,(Coix_aquatica:0.009761072261,Saccharum_arundinaceum:0.
009761072261):0.0111080886):0.02254154999,(((Calamagrostis_effusiflora:0.00642442078,(Ech
inochloa_oryzoides:0.001456876457,Paspalidium_flavidum:0.001456876457):0.004967544324):
0.006479823593,(Stipa_sareptana:0.007583368667,(Paspalum_longifolium:0.002691944806,Stip
a_pappiformis:0.002691944806):0.004891423861):0.005320875707):0.01912866574,(Panicum_k
hasianum:0.01418945016,(Echinochloa_glabrescens:0.005987357843,Paspalum_thunbergii:0.005
987357843):0.008202092317):0.01784345996):0.01137780073):0.05107363672,((Poa_szechuens
is:0.02106275253,(Puccinellia_minuta:0.02017446229,(Puccinellia_hauptiana:0.01221177921,Pu
ccinellia_leiolepis:0.01221177921):0.007962683075):0.0008882902444):0.04887238763,((((Phle
um_paniculatum:0.00999049146,(Alopecurus_aequalis:0.002733758902,Isachne_clarkei:0.00273
3758902):0.007256732558):0.005420254478,(Hemarthria_compressa:0.01248188561,Isachne_si
kkimensis:0.01248188561):0.002928860324):0.01345718337,(((Echinochloa_colona:0.00229669
5993,(Ischaemum_rugosum:0,Polypogon_fugax:0):0.002296695993):0.006528294302,(Agrostis_
clavata:0.005328486798,Isachne_globosa:0.005328486798):0.003496503497):0.01515151515,((L
eptochloa_fusca:0.004780042554,(Festuca_parvigluma:0.001748251748,Glyceria_acutiflora:0.00
1748251748):0.003031790806):0.008846798992,(Helictotrichon_schmidii:0.008819149239,Sacci
olepis_indica:0.008819149239):0.004807692308):0.0103496639):0.004891423861):0.031428384
07,((((Bromus_japonicus:0.005078925631,(Alopecurus_arundinaceus:0.002151008347,Polypogo
n_monspeliensis:0.002151008347):0.002927917284):0.01071308863,((Agrostis_gigantea:0.0024
22490512,(Helictotrichon_junghuhnii:0.001165501166,Leymus_secalinus:0.001165501166):0.00
1256989347):0.008172672529,((Festuca_extremiorientalis:0.001602564103,Panicum_bisulcatum:
0.001602564103):0.006625511239,(Festuca_leptopogon:0.002476689977,Poa_pratensis:0.00247
6689977):0.005751385365):0.002367087699):0.005196851222):0.01194638695,(((Calamagrostis
_purpurea:0.004628935165,(Festuca_procera:0.001705084067,Melica_przewalskyi:0.001705084
067):0.002923851099):0.007419819513,(Poa_acroleuca:0.006535802088,(Elymus_nutans:0.0021
43500561,Glyceria_tonglensis:0.002143500561):0.004392301527):0.00551295259):0.010313368
01,(Glyceria_arundinacea:0.01410863977,Poa_palustris:0.01410863977):0.008253482922):0.005
37627852):0.02104695588,(Deschampsia_cespitosa:0.03269939225,((Festuca_forrestii:0.004891
423861,Puccinellia_schischkinii:0.004891423861):0.01230732942,((Festuca_japonica:0.0044401
96554,(Arthraxon_hispidus:0.001748251748,Piptatherum_laterale:0.001748251748):0.002691944
806):0.005765549735,(Microstegium_nudum:0.005328486798,Poa_tibetica:0.005328486798):0.0

04877259491):0.006993006993):0.01550063897):0.01608596483):0.0115109563):0.0096388267
78):0.02454920741):0.06816842464,((((Suaeda_glauca:0.01515151515,Suaeda_paradoxa:0.0151
5151515):0.01593770191,(Suaeda_maritima:0.01849051235,Suaeda_stellatiflora:0.01849051235):
0.01259870471):0.02900366015,(Chenopodium_album:0.04334712127,(Chenopodium_gracilispi
cum:0.02685595202,(Atriplex_laevis:0.006188448302,Dysphania_schraderiana:0.006188448302):
0.02066750372):0.01649116924):0.01674575594):0.09195646234,((Lasia_spinosa:0.0191252422
9,Pinellia_pedatisecta:0.01912524229):0.1232990001,(Hippophae_neurocarpa:0.03230591345,Hi
ppophae_tibetana:0.03230591345):0.110118329):0.009625097125):0.01060343266):0.028771608
27,(((Carex_stipitiutriculata:0.02820144717,(Carex_breviculmis:0.01724889072,Cyperus_nigrofu
scus:0.01724889072):0.01095255645):0.06377038292,(((Scirpus_orientalis:0.01425140625,(Care
x_subfilicinoides:0.008202092317,Cyperus_exaltatus:0.008202092317):0.006049313936):0.0172
3305962,((Carex_lehmannii:0.009615384615,Carex_vulpina:0.009615384615):0.01765734266,((
Cyperus_pilosus:0.002913752914,(Carex_fluviatilis:0.0019330649,Carex_pruinosa:0.001933064
9):0.0009806880138):0.008791396179,(Carex_dimorpholepis:0.003255265643,(Carex_longipes:
0.0009987741,Carex_phacota:0.0009987741):0.002256491543):0.00844988345):0.01556757818):
0.004211738601):0.03784682723,((((Carex_gibba:0.006056925027,Cyperus_iria:0.00605692502
7):0.008423900398,(Carex_thomsonii:0.008361944306,Lipocarpha_chinensis:0.008361944306):0.
006118881119):0.01495162807,((Cyperus_compressus:0.01047769181,Pycreus_sanguinolentus:0.
01047769181):0.01474398433,(Cyperus_cyperoides:0.01569423067,Cyperus_duclouxii:0.015694
23067):0.009527445471):0.004210777355):0.02266531664,(((Carex_alta:0.007353949468,Carex
_ovatispiculata:0.007353949468):0.01047769184,(Carex_nubigena:0.01477220074,(Scleria_parv
ula:0.008895269701,(Carex_gonggaensis:0.006488146908,(Carex_remotiuscula:0,Eriophorum_gr
acile:0):0.006488146908):0.002407122794):0.005876931043):0.003059440559):0.01745653443,(
(Carex_forrestii:0.007575757576,(Carex_cylindrostachys:0,Fimbristylis_henryi:0):0.0075757575
76):0.01508955906,(Carex_speciosa:0.0196335258,(Fimbristylis_ovata:0.005064761233,Kobresi
a_fragilis:0.005064761233):0.01456876457):0.003031790833):0.0126228591):0.0168095944):0.
01723352297):0.02264053699):0.07837640432,((Rhodiola_himalensis:0.03089562536,(Rhodiola
_heterodonta:0.004164624533,Scheuchzeria_palustris:0.004164624533):0.02673100083):0.11428
86814,(Galium_bungei:0.04311698984,(Hydrocotyle_salwinica:0.03676271193,(Chamaesium_vi
ridiflorum:0.01959171171,(Galium_elegans:0.009830639444,Galium_spurium:0.009830639444):
0.009761072261):0.01717100022):0.006354277916):0.1020673169):0.02516392764):0.0210761
4608):0.09460903055,(((Festuca_nitidula:0.007404086878,Puccinellia_ladyginii:0.007404086878)
:0.07550136947,(Eragrostis_japonica:0.04783908872,Phragmites_karka:0.04783908872):0.03506
636763):0.1131189727,(((Neyraudia_reynaudiana:0.0335475351,(Hemarthria_altissima:0.024956
17157,(Hierochloe_odorata:0.008347779963,Microstegium_ciliatum:0.008347779963):0.016608
39161):0.008591363532):0.06204035754,(Puccinellia_strictura:0.05523662235,((Agropogon_lut
osus:0,Calamagrostis_stricta:0):0.0202387641,(Trisetum_bifidum:0.01535495133,(Puccinellia_ch
inampoensis:0.006252750111,(Festuca_tristis:0.002636541993,Puccinellia_multiflora:0.0026365
41993):0.003616208118):0.009102201216):0.00488381277):0.03499785825):0.04035127029):0.
07395812876,((Sacciolepis_myosuroides:0.07596862302,(((Calamagrostis_arundinacea:0.005911
237381,Leptochloa_chinensis:0.005911237381):0.01796316458,(Arundinella_hirta:0.0182545398
7,((Agrostis_vinealis:0.001271041417,Puccinellia_gigantea:0.001271041417):0.008996660965,(
Calamagrostis_pseudophragmites:0.003129007743,Stipa_hookeri:0.003129007743):0.007138694
639):0.007986837489):0.00561986209):0.03273536529,(((Agrostis_hookeriana:0.001705084067,

Poa_binodis:0.001705084067):0.02459797837,(((Littledalea_racemosa:0.002185314685,Puccinel
lia_stapfiana:0.002185314685):0.004927396901,(Deyeuxia_zangxiensis:0.002622377622,(Agrost
is_hugoniana:0.001165501166,Puccinellia_pauciramea:0.001165501166):0.001456876457):0.004
490333964):0.007464376296,(Puccinellia_hackeliana:0.009186644992,(Puccinellia_altaica:0.003
205128205,Puccinellia_roborovskyi:0.003205128205):0.005981516787):0.00539044289):0.0117
2597455):0.0218642802,(((Muhlenbergia_japonica:0.008493467581,Phleum_alpinum:0.0084934
67581):0.01777389277,(Catabrosa_aquatica:0.008639155227,(Poa_alpina:0,Stipa_concinna:0):0.
008639155227):0.01762820513):0.01723472635,(((Poa_bomiensis:0.006701631702,Puccinellia_
distans:0.006701631702):0.01235746683,((Poa_supina:0.006056925027,Ptilagrostis_junatovii:0.
006056925027):0.008715275689,((Puccinellia_przewalskii:0.003205128205,(Poa_sikkimensis:0,
Stipa_subsessiliflora:0):0.003205128205):0.005897073011,(Puccinellia_poecilantha:0.004003133
617,(Poa_nubigena:0.001311188811,Puccinellia_tenuiflora:0.001311188811):0.002691944806):0.
005099067599):0.0056699995):0.004286897817):0.01114182828,(Festuca_coelestis:0.02165966
749,Puccinellia_ladakhensis:0.02165966749):0.008541259331):0.01330115989):0.004665255935)
:0.008442424614):0.01935885577):0.08784203926,(((Calamagrostis_epigeios:0.01239761425,(A
pluda_mutica:0.002622377622,Echinochloa_crus.galli:0.002622377622):0.009775236631):0.029
62999489,(Phalaris_arundinacea:0.03414477191,(Pennisetum_flaccidum:0.01732112548,(Elymus
_burchan.buddae:0.0134032634,Poa_grandis:0.0134032634):0.003917862074):0.01682364644):0.
007882837227):0.03413683894,(((Agrostis_micrantha:0.00528805772,Elymus_pulanensis:0.005
28805772):0.01313788384,(Puccinellia_nudiflora:0.008875127705,Puccinellia_pamirica:0.00887
5127705):0.009550813853):0.0323256601,(Pogonatherum_crinitum:0.03391923839,((Poa_callio
psis:0.01648586742,(Puccinellia_degeensis:0.008519450633,(Puccinellia_arjinshanensis:0,Puccin
ellia_himalaica:0):0.008519450633):0.007966416791):0.01063547684,((Puccinellia_dolicholepis:
0.003059440559,Puccinellia_micrandra:0.003059440559):0.02046583758,(Poa_nemoralis:0.0100
6077087,(Poa_arctica:0.0019330649,Polypogon_maritimus:0.0019330649):0.008127705967):0.0
1346450727):0.003596066122):0.006797894132):0.01683236326):0.02541284642):0.087646214
2):0.00573535912):0.02647840762):0.09000898201):0.0204540189):0.01169438825):0.0332920
5552):0.1220838186,(((((Schoenoplectiella_mucronata:0.1392083438,(Acorus_calamus:0.114254
3078,((Sparganium_glomeratum:0.001666685846,Sparganium_limosum:0.001666685846):0.0119
4021754,(Sparganium_emersum:0.003491178367,Sparganium_fallax:0.003491178367):0.010115
72502):0.1006474044):0.02495403605):0.1012737004,((((Typha_angustifolia:0.008387927358,T
ypha_orientalis:0.008387927358):0.01943420541,(Typha_laxmannii:0.01425430776,((Schoenopl
ectus_tabernaemontani:0,Typha_davidiana:0):0.005163398667,(Schoenoplectiella_juncoides:0.00
09987741,Typha_lugdunensis:0.0009987741):0.004164624567):0.009090909091):0.0135678250
1):0.02463644286,((Eleocharis_ovata:0.01759999259,Schoenoplectus_triqueter:0.01759999259):
0.01801111709,(Eleocharis_valleculosa:0.02135680192,(Eleocharis_penchaoi:0.002231559633,El
eocharis_qinghaiensis:0.002231559633):0.01912524229):0.01425430776):0.01684746594):0.105
5081101,((Caldesia_parnassifolia:0.0253378317,Typha_latifolia:0.0253378317):0.09473045148,(
Typha_domingensis:0.04087195245,Typha_minima:0.04087195245):0.07919633072):0.0378984
0256):0.08251535848):0.02033019584,((Dopatrium_junceum:0.1259292417,(Juncus_effusus:0.1
068039994,(Monochoria_vaginalis:0.005514256767,Veronica_anagallis.aquatica:0.00551425676
7):0.1012897427):0.01912524226):0.1227919081,((((Alisma_canaliculatum:0.006847319347,Ali
sma_plantago.aquatica:0.006847319347):0.04447831609,((Alisma_gramineum:0.01238344988,A
lisma_orientale:0.01238344988):0.02682069118,(Sagittaria_pygmaea:0.0174288847,(Sagittaria_t

engtsungensis:0.002639374867,Sagittaria_trifolia:0.002639374867):0.01478950984):0.02177525
636):0.01212149438):0.08534697732,(Sparganium_confertum:0.05448907623,Sparganium_stolo
niferum:0.05448907623):0.08218353653):0.1025212959,((Glyceria_maxima:0.09519178114,Phr
agmites_australis:0.09519178114):0.04587323646,(Beckmannia_syzigachne:0.1003787879,Leers
ia_japonica:0.1003787879):0.04068622971):0.09812889106):0.009527241148):0.01209109025):
0.1596423054,(((Lemna_trisulca:0.0303030303,Ottelia_acuminata:0.0303030303):0.1556672494,
(((Batrachium_bungei:0.0249821546,(Batrachium_foeniculaceum:0.01201595413,Batrachium_tri
chophyllum:0.01201595413):0.01296620047):0.02764022303,(Batrachium_eradicatum:0.036188
81119,Hydrilla_verticillata:0.03618881119):0.01643356643):0.09265734266,(Vallisneria_natans:
0.1107808858,(Blyxa_echinosperma:0.0298951049,Blyxa_japonica:0.0298951049):0.080885780
89):0.0344988345):0.04069055944):0.09812062937,((Myriophyllum_spicatum:0.1164858674,M
yriophyllum_verticillatum:0.1164858674):0.134912734,((Stuckenia_pectinata:0.03398459804,(R
uppia_maritima:0.01704545455,(Potamogeton_gramineus:0.003933566434,Stuckenia_pamirica:0.
003933566434):0.01311188811):0.0169391435):0.1404035138,((((Stuckenia_amblyophylla:0.012
26589283,(Potamogeton_pusillus:0.006555944056,(Potamogeton_compressus:0.00152972028,St
uckenia_filiformis:0.00152972028):0.005026223776):0.005709948777):0.0289829455,(Potamog
eton_octandrus:0.02132867133,(Potamogeton_obtusifolius:0.006993006993,Potamogeton_oxyph
yllus:0.006993006993):0.01433566434):0.019920167):0.01189801482,((Potamogeton_lucens:0.0
1311188811,(Potamogeton_crispus:0.003321678322,Potamogeton_perfoliatus:0.003321678322):0.
00979020979):0.02915676374,(Zannichellia_palustris:0.02850621601,(Potamogeton_maackianus:
0.01538461538,Potamogeton_nodosus:0.01538461538):0.01312160062):0.01376243584):0.0108
782013):0.1003551553,(Ceratophyllum_demersum:0.1278793547,(Najas_marina:0.03624603316,
Najas_minor:0.03624603316):0.09163332157):0.02562265371):0.02088610345):0.07701048951):
0.03269230769):0.1363636364):0.02272727273,(((Trapa_incisa:0.2533216783,(Nymphaea_tetra
gona:0.1197686383,(Smilax_glabra:0.04475099754,((Ophiopogon_bodinieri:0.02294664331,(Act
aea_asiatica:0.012108009,Sambucus_adnata:0.012108009):0.01083863431):0.0168192088,(Ophi
opogon_yunnanensis:0.03064948652,Vaccinium_uliginosum:0.03064948652):0.009116365586):0.
00498514543):0.07501764076):0.13355304):0.07644230769,((((((Androsace_yargongensis:0.010
09603171,(Androsace_zambalensis:0.004198958701,Saxifraga_nana:0.004198958701):0.005897
073011):0.01710320305,(Arenaria_aksayqingensis:0.01726070937,(Thylacospermum_caespitosu
m:0.01050367486,(Arenaria_stracheyi:0.003205128205,Stellaria_uliginosa:0.003205128205):0.0
07298546655):0.006757034515):0.009938525384):0.04846542347,(((Viola_sikkimensis:0.02316
433566,(((Goodyera_foliosa:0.003238657221,Saxifraga_melanocentra:0.003238657221):0.00259
6394598,(Peracarpa_carnosa:0.004294508908,Plantago_minuta:0.004294508908):0.00154054291
1):0.01429155365,(Saxifraga_divaricata:0.01150932401,(Parnassia_farreri:0.00101981352,Swerti
a_dichotoma:0.00101981352):0.01048951049):0.008617281457):0.003037730198):0.015362497
32,((Arenaria_tumengelaensis:0.008318360174,(Arenaria_dsharaensis:0.003656355512,Primula_
buryana:0.003656355512):0.004662004662):0.0154085841,(Primula_lactucoides:0.01144152686,
(Parnassia_cacuminum:0.006745215861,Veronica_chayuensis:0.006745215861):0.004696311):0.
01228541742):0.0147998887):0.01923915764,(((Epilobium_minutiflorum:0.01590404183,((Andr
osace_tanggulashanensis:0,Mazus_humilis:0):0.01083916084,(Arenaria_neelgherrensis:0.008519
450633,Arenaria_puranensis:0.008519450633):0.002319710206):0.005064880994):0.012321242
86,(((Juncus_ranarius:0.002199479056,Parnassia_pusilla:0.002199479056):0.003670229578,((An
drosace_gmelinii:0.001705084067,Arenaria_edgeworthiana:0.001705084067):0.002905005867,(

Aletris_alpestris:0.00202392268,Centaurium_pulchellum:0.00202392268):0.002586167254):0.00
12596187):0.01071112508,(Stellaria_uda:0.01308070848,(Pedicularis_reptans:0.0005244755245,
Stellaria_mainlingensis:0.0005244755245):0.01255623296):0.003500125226):0.01164445098):0.
02055093909,((((Leontopodium_franchetii:0.00101981352,Oldenlandia_diffusa:0.00101981352):
0.008810825924,(Lysimachia_parvifolia:0.003711758325,Stellaria_palustris:0.003711758325):0.
006118881119):0.01668956115,((Saxifraga_cernua:0.009667685606,(Cerastium_thomsonii:0,Ped
icularis_oederi:0):0.009667685606):0.01269209137,((Arenaria_trichophora:0.006931050901,Gen
tiana_algida:0.006931050901):0.009906759907,(Pseudostellaria_tibetica:0.006993006993,(Arena
ria_zhongdianensis:0.005460010074,Parnassia_chinensis:0.005460010074):0.001532996919):0.0
09844803814):0.005521966166):0.004160423618):0.01730336018,((Sagina_japonica:0.0086734
61593,Sagina_saginoides:0.008673461593):0.02388856633,((Juncus_modicus:0.00521877222,Sp
ergularia_marina:0.00521877222):0.01348699496,(Drosera_peltata:0.01602143346,(Gentiana_he
leonastes:0.007076738546,Juncus_leucomelas:0.007076738546):0.008944694916):0.0026843337
15):0.01385626075):0.01126153285):0.004952663005):0.00898976685):0.0178986676):0.05192
255039,((((Oldenlandia_verticillata:0.02317850003,Oxalis_griffithii:0.02317850003):0.02304806
778,(((Viola_chaerophylloides:0.004164624533,(Eriocaulon_henryanum:0.0009405074167,Linde
rnia_anagallis:0.0009405074167):0.003224117117):0.009795406401,(Corydalis_iochanensis:0.00
6084574753,Euphrasia_regelii:0.006084574753):0.007875456181):0.01858606256,((Juncus_allio
ides:0.002272727273,Neanotis_hirsuta:0.002272727273):0.01913914905,((Epilobium_sinense:0.
005322645742,(Pedicularis_oxycarpa:0.004816015622,(Juncus_concinnus:0.002855694685,(Parn
assia_oreophila:0.0009617552914,Torenia_violacea:0.0009617552914):0.001893939394):0.0019
60320936):0.0005066301202):0.007916558054,(Impatiens_mussotii:0.006348300318,(Impatiens
_apsotis:0.0002913752914,Lysimachia_candida:0.0002913752914):0.006056925027):0.00689090
3478):0.008172672529):0.01113421716):0.01368047432):0.02236973381,((Circaea_alpina:0.002
295029291,Viola_fargesii:0.002295029291):0.04556008568,(Gentiana_leucomelaena:0.03138059
235,Viola_grandisepala:0.03138059235):0.01647452262):0.02074118665):0.04997243733,(((Eo
mecon_chionantha:0.01294782853,Phaius_tankervilleae:0.01294782853):0.01813349906,((Alliu
m_trifurcatum:0.01085045296,(Pedicularis_vialii:0.005314322428,(Circaea_repens:0.004516317
016,Lysimachia_barystachys:0.004516317016):0.0007980054116):0.005536130536):0.01425724
731,((Habenaria_dentata:0.004011023081,Lysimachia_clethroides:0.004011023081):0.008575429
127,(Allium_victorialis:0.007625182791,Zingiber_striolatum:0.007625182791):0.004961269418):
0.01252124806):0.005973627323):0.04025705624,(Salix_dibapha:0.0591006216,((((Saxifraga_p
allida:0.008148229466,(Houttuynia_cordata:0.001456876457,Saxifraga_rufescens:0.0014568764
57):0.00669135301):0.009318583656,(Plantago_major:0.01250315448,(Plantago_asiatica:0.0074
04086878,(Parnassia_nubicola:0.004198958673,(Aletris_gracilis:0,Corydalis_nigroapiculata:0):0.
004198958673):0.003205128205):0.005099067599):0.004963658645):0.01988697267,(((Impatie
ns_membranifolia:0.007692307692,Salix_phanera:0.007692307692):0.006435186348,(Impatiens
_nubigena:0.007950864421,Impatiens_toxophora:0.007950864421):0.00617662962):0.01806526
807,((Gentiana_officinalis:0.00653580206,(Liparis_petiolata:0.002969155727,Plantago_depressa:
0.002969155727):0.003566646333):0.01058156536,((Cephalanthera_longifolia:0.003326485145,
Lysimachia_stenosepala:0.003326485145):0.009599680255,((Allium_hookeri:0.002927917284,A
llium_ovalifolium:0.002927917284):0.005099067599,(Impatiens_brachycentra:0.003364980221,I
mpatiens_wilsonii:0.003364980221):0.004662004662):0.004899180517):0.004191202017):0.015
07539469):0.005161023691):0.009159949745,(((Lysimachia_fortunei:0.009178321678,(Rhodode

ndron_temenium:0.004516317016,Stellaria_chinensis:0.004516317016):0.004662004662):0.0101
981352,((Penthorum_chinense:0.004953379953,Veronica_oxycarpa:0.004953379953):0.0059731
93473,(Comarum_salesovianum:0.006334135948,(Aletris_stenoloba:0.0001748251748,Pinguicul
a_alpina:0.0001748251748):0.006159310773):0.004592437479):0.00844988345):0.0190407266,(
((Impatiens_margaritifera:0.01053309462,(Parnassia_palustris:0.002913752914,Veronica_javanic
a:0.002913752914):0.007619341707):0.009016929673,((Parnassia_wightiana:0.006035379611,(
Aletris_laxiflora:0.001878235034,Parnassia_crassifolia:0.001878235034):0.004157144577):0.005
968755865,((Allium_yanchiense:0.003642191142,Epilobium_palustre:0.003642191142):0.00791
6558082,(Lysimachia_platypetala:0.006535802088,(Lysimachia_lobelioides:0.003059440559,Tri
adenum_breviflorum:0.003059440559):0.003476361528):0.005022947137):0.0004453862512):0.
007545888818):0.00948540133,((Lysimachia_tsarongensis:0.006546205136,Neanotis_wightiana:
0.006546205136):0.0169533345,((Parnassia_trinervis:0.006861483718,(Parnassia_laxmannii:0.00
3364980221,(Parnassia_gansuensis:0.002185314685,Pedicularis_longistipitata:0.002185314685):
0.001179665536):0.003496503497):0.007124774813,((Primula_involucrata:0.003600695955,(Jun
cus_gracilicaulis:0.001664942465,(Juncus_clarkei:0.0005412553957,Parnassia_delavayi:0.00054
12553957):0.001123687069):0.00193575349):0.005911237381,(Parnassia_noemiae:0.004308428
733,(Amitostigma_basifoliatum:0.002317082506,Parnassia_brevistyla:0.002317082506):0.00199
1346228):0.005203504603):0.004474325195):0.009513281101):0.005535885991):0.0093817578
53):0.008096552066):0.01258688606):0.01223776224):0.04723035512):0.009018469662):0.057
12792904,((Limosella_aquatica:0.06144999586,Nymphoides_indica:0.06144999586):0.06977605
897,(Ludwigia_adscendens:0.1099062333,(Eriocaulon_buergerianum:0.03201697569,Menyanthe
s_trifoliata:0.03201697569):0.07788925766):0.02131982149):0.05348908283):0.07542472248,(((
((Draba_glomerata:0.005474174444,Eutrema_fontanum:0.005474174444):0.01409155424,(Aphr
agmus_oxycarpus:0.01746414555,(Dilophia_ebracteata:0.01098432196,Draba_lichiangensis:0.01
098432196):0.006479823593):0.002101583132):0.01646270396,(Dilophia_salsa:0.02809934368,
(Draba_mongolica:0.01162902863,(Draba_parviflora:0.004490333992,Smelowskia_tibetica:0.00
4490333992):0.007138694639):0.01647031505):0.007929088959):0.05241315427,(Pegaeophyto
n_scapiflorum:0.04102774244,(Eutrema_himalaicum:0.02664902051,(Eutrema_yunnanense:0.01
82185668,Lepidium_latifolium:0.0182185668):0.008430453705):0.01437872194):0.0474138444
7):0.1106464903,(((((Pleurospermum_nanum:0.007394958333,Tongoloa_tenuifolia:0.007394958
333):0.02822474009,((Oenanthe_benghalensis:0.006133045489,(Galium_asperifolium:0.0043847
93741,Galium_boreale:0.004384793741):0.001748251748):0.02561504262,(Galium_tricornutum:
0.0127325737,Oenanthe_hookeri:0.0127325737):0.0190155144):0.003871610314):0.0398740599
6,((Galium_paradoxum:0.01710320307,Physospermopsis_kingdon.wardii:0.01710320307):0.029
86596737,(Galium_exile:0.01956572868,(Chamaesium_novemjugum:0.007208261822,Sium_frig
idum:0.007208261822):0.01235746686):0.02740344176):0.02852458794):0.03388747292,((((Di
ckinsia_hydrocotyloides:0.003324588282,Hydrocotyle_javanica:0.003324588282):0.0139602754
8,(Sanicula_chinensis:0.01172457884,Sanicula_elata:0.01172457884):0.005560284922):0.023286
11344,((Hydrocotyle_sibthorpioides:0.01486013986,(Pimpinella_diversifolia:0.007264240316,(Pi
mpinella_coriacea:0.003607884804,(Oenanthe_linearis:0.003205128205,Pimpinella_yunnanensis:
0.003205128205):0.0004027565987):0.003656355512):0.007595899544):0.01748251748,((Pimpi
nella_rhomboidea:0.002476689977,(Pimpinella_candolleana:0.0004370629371,Pimpinella_henry
i:0.0004370629371):0.00203962704):0.02210016716,((Hydrocotyle_wilfordii:0.007513801483,S
anicula_hacquetioides:0.007513801483):0.01359253518,(Galium_yunnanense:0.01035798721,Sa

nicula_astrantiifolia:0.01035798721):0.01074834945):0.003470520472):0.007765800207):0.0082
28319859):0.03565279902,((((Anthriscus_sylvestris:0.007575757576,Pleurospermum_rivulorum:
0.007575757576):0.02229782088,((Ligusticum_pteridophyllum:0.004440196554,Saururus_chine
nsis:0.004440196554):0.01471445221,(Ligusticum_jeholense:0.01565814527,(Cicuta_virosa:0.00
6861483718,Ligusticum_brachylobum:0.006861483718):0.008796661554):0.003496503497):0.0
1071892969):0.0057222167,((Ligusticum_sikiangense:0.01305202099,(Pimpinella_smithii:0.004
253013721,Pleurospermum_angelicoides:0.004253013721):0.00879900727):0.01968199654,((Cn
idium_salinum:0.008889292103,(Oenanthe_javanica:0.002636541993,Pleurospermum_franchetia
num:0.002636541993):0.006252750111):0.01646270396,((Ligusticum_tachiroei:0.007629080627,
(Hydrocotyle_himalaica:0.003001382331,Hydrocotyle_hookeri:0.003001382331):0.00462769829
6):0.0110091991,(Oenanthe_thomsonii:0.01006661192,Pleurospermum_linearilobum:0.01006661
192):0.008571667805):0.006713716338):0.007382021464):0.002861777627):0.02363127949,(G
alium_dahuricum:0.04010347916,((Carum_carvi:0.01309174614,Galium_innocuum:0.013091746
14):0.01379267116,(((Galium_asperuloides:0,Galium_hoffmeisteri:0):0.0157158938,((Angelica_
grosseserrata:0.001456876457,Pimpinella_fargesii:0.001456876457):0.004739895159,(Pimpinell
a_brachystyla:0.003504826811,(Pimpinella_chungdienensis:0.001456876457,Pimpinella_silvatic
a:0.001456876457):0.002047950354):0.002691944806):0.009519122185):0.008220464251,((Gal
ium_karataviense:0.006595757905,Tongoloa_elata:0.006595757905):0.01152725869,(Cnidium_
monnieri:0.007619341735,Pleurospermum_aromaticum:0.007619341735):0.01050367486):0.005
813341457):0.002948059252):0.01321906186):0.01912359549):0.01699670158):0.03315745507)
:0.07263698126,((((Rubus_corchorifolius:0.01290424437,(Chloranthus_elatior:0.005328486798,
Rubus_paniculatus:0.005328486798):0.007575757576):0.0229307089,((Rubus_amabilis:0.01622
90772,(Rubus_tsangii:0.01098432196,((Rubus_eustephanos:0.0004370629371,Rubus_trijugus:0.
0004370629371):0.006555944056,(Rubus_alexeterius:0.002272727273,Rubus_nyalamensis:0.00
2272727273):0.00472027972):0.003991314963):0.005244755245):0.01098432193,((Rubus_pung
ens:0.005973193473,(Rubus_biflorus:0.002768065268,Sambucus_javanica:0.002768065268):0.0
03205128205):0.01246718144,((Rubus_malifolius:0.002922076228,Rubus_mesogaeus:0.002922
076228):0.008936371574,(Rubus_hunanensis:0.004516317016,Rubus_wallichianus:0.004516317
016):0.007342130786):0.006581927108):0.008773024218):0.008621554148):0.02572366475,(((
Lagotis_brachystachya:0,Rubus_assamensis:0):0.02235557606,(Rubus_pectinellus:0.0118006993,
Rubus_rubrisetulosus:0.0118006993):0.01055487676):0.02235557602,((Chloranthus_serratus:0.0
1142138489,Rubus_pinnatisepalus:0.01142138489):0.02025058275,(Chloranthus_multistachys:0.
01835664336,Pachysandra_axillaris:0.01835664336):0.01331532429):0.01303918444):0.016847
46594):0.1022777763,((((Kyllinga_brevifolia:0.01462651307,Lipocarpha_microcephala:0.01462
651307):0.02685430525,(Lycopus_lucidus:0.02856348217,((Teucrium_viscidum:0.00933233263
8,(Lycopus_europaeus:0.003230333767,Schoenoplectus_subulatus:0.003230333767):0.00610199
8871):0.01302324342,(Carex_japonica:0.01263885213,(Carex_yunlingensis:0.00637428337,Kob
resia_loliacea:0.00637428337):0.006264568765):0.009716723923):0.00620790611):0.012917336
15):0.02620647689,((Eritrichium_pseudolatifolium:0.0229307089,(Actinocarya_tibetica:0.01214
982312,Eritrichium_sessilifructum:0.01214982312):0.01078088578):0.0282450313,((Carex_roch
ebrunii:0.008609735466,Lycopus_cavaleriei:0.008609735466):0.01631701632,(Carex_rubrobrun
nea:0.01172457884,Scutellaria_yunnanensis:0.01172457884):0.01320217294):0.02624898842):0.
016511555):0.08712590202,((Sorbaria_kirilowii:0.07554463174,(Asteropyrum_cavaleriei:0.0447
11621,Saxifraga_clavistaminea:0.044711621):0.03083301075):0.07459628218,(Alternanthera_se

ssilis:0.1167711538,((Poa_annua:0.0159040418,(Poa_nepalensis:0.005869708633,(Poa_lipskyi:0.
002639374867,Stipa_bungeana:0.002639374867):0.003230333767):0.01003433317):0.03474669
635,(Isachne_albens:0.03560553408,Saccharum_spontaneum:0.03560553408):0.01504520407):0.
06612041568):0.0333697601):0.004672283299):0.009023197146):0.01818181818):0.017069864
62):0.06105178297):0.06962412587):0.1020541958,((Brasenia_schreberi:0.25,(((Pedicularis_gla
brescens:0.003846153846,(Arisaema_griffithii:0,Liriope_kansuensis:0):0.003846153846):0.0446
3272057,(Ophiopogon_intermedius:0.0375430576,(Ophiopogon_angustifoliatus:0.01660839161,
Ophiopogon_japonicus:0.01660839161):0.02093466599):0.01093581682):0.09924839831,((Acon
itum_bulleyanum:0.0389705143,(Aconitum_pseudobrunneum:0.008741258741,Aconitum_tangut
icum:0.008741258741):0.03022925556):0.08320778766,(Megacarpaea_delavayi:0.1178512567,(
Angelica_decursiva:0.02118245715,Pimpinella_kingdon.wardii:0.02118245715):0.09666879959):
0.004327045212):0.02554897077):0.1022727273):0.1704545455,((Potamogeton_wrightii:0.2187
062937,(Phoebe_hunanensis:0.1281468531,(Potamogeton_distinctus:0.01363636364,Potamogeto
n_natans:0.01363636364):0.1145104895):0.09055944056):0.1903846154,((Persicaria_amphibia:0.
1704545455,(((Saussurea_nimborum:0.01308590506,Saussurea_ovatifolia:0.01308590506):0.038
44316653,((Centipeda_minima:0.009090909091,Polygonum_plebeium:0.009090909091):0.02821
615138,((Polygonum_glaciale:0.00641025641,Polygonum_humile:0.00641025641):0.016255060
22,(Polygonum_cognatum:0.01777389277,Saussurea_subulata:0.01777389277):0.004891423861):
0.01464174384):0.01422201112):0.05668168015,((((Pilea_pseudonotata:0.005801522803,Rumex
_nepalensis:0.005801522803):0.01450680848,((Persicaria_filiformis:0.003857445971,(Pilea_hilli
ana:0.001923076923,Polygonum_dissitiflorum:0.001923076923):0.001934369048):0.013969672
79,(Carduus_crispus:0.01011019608,(Boehmeria_macrophylla:0.0006993006993,Cirsium_souliei:
0.0006993006993):0.009410895382):0.007716922675):0.002481212523):0.02294278514,(Debre
geasia_orientalis:0.03502278347,Rheum_palmatum:0.03502278347):0.00822833295):0.0294168
2819,((Rheum_moorcroftianum:0.02197285147,Thalictrum_squamiferum:0.02197285147):0.035
080532,((Polygonum_runcinatum:0.02049416632,(((Artemisia_tangutica:0.001857966326,Persic
aria_maculosa:0.001857966326):0.00452464033,(Cirsium_arvense:0.002485013291,Polygonum_
posumbu:0.002485013291):0.003897593366):0.008243906414,(Persicaria_nepalensis:0.0089806
67956,(Persicaria_capitata:0.003496503497,(Polygonum_maackianum:0.0005827505828,Swertia
_punicea:0.0005827505828):0.002913752914):0.00548416446):0.005645845114):0.00586765325)
:0.0238526265,(((Polygonum_tibeticum:0.008158508159,(Polygonum_sinomontanum:0.0033106
68429,Saussurea_ussuriensis:0.003310668429):0.00484783973):0.01067878227,(Syncalathium_r
oseum:0.00844988345,(Polygonum_macrophyllum:0.001602564103,(Oxyria_digyna:0.00043706
29371,Rumex_ucranicus:0.0004370629371):0.001165501166):0.006847319347):0.01038740697):
0.0178717887,(((Persicaria_lapathifolia:0.005947210449,(Polygonum_suffultoides:0.0013111888
11,Saussurea_acroura:0.001311188811):0.004636021638):0.01653227115,((Rumex_aquaticus:0.0
04767544913,(Persicaria_campanulata:0.001271041417,Persicaria_wallichii:0.001271041417):0.
003496503497):0.00818028362,(Petasites_tricholobus:0.008533615003,(Pilea_sinofasciata:0.004
953379953,(Lecanthus_peduncularis:0.002331002331,Polygonum_paleaceum:0.002331002331):
0.002622377622):0.00358023505):0.00441421353):0.009531653062):0.008136699461,(Polygon
um_argyrocoleon:0.02749899197,(((Persicaria_chinensis:0.004585884199,(Polygonum_viscosum:
0.003606218074,(Saussurea_amara:0.001602564103,Saussurea_parviflora:0.001602564103):0.00
2003653972):0.0009796661253):0.006450403805,(Persicaria_longiseta:0.009844803814,(Polygo
num_praetermissum:0.004370629371,Polygonum_thunbergii:0.004370629371):0.005474174444):

0.00119148419):0.01246300711,(((Clematis_pseudopogonandra:0.001165501166,Polygonum_mu
ricatum:0.001165501166):0.004821856678,(Artemisia_mongolica:0.004053271055,(Persicaria_st
rigosa:0.002290854936,(Polygonum_hastatosagittatum:0,Polygonum_umbrosum:0):0.002290854
936):0.001762416118):0.001934086788):0.007208261822,(Polygonum_senticosum:0.006118881
119,Sanguisorba_officinalis:0.006118881119):0.007076738546):0.01030367545):0.00399969685
2):0.003117189089):0.006092898067):0.007637713696):0.01270659065):0.01561456114):0.035
54280713):0.06224379371):0.1382867133,((Hippuris_vulgaris:0.1706094191,((Ranunculus_min
or:0.06108250011,((Ranunculus_hirtellus:0.01396003096,(Artemisia_minor:0.004370629371,Ast
er_semiprostratus:0.004370629371):0.009589401591):0.02809934366,(Thalictrum_rutifolium:0.0
1894283068,(Saussurea_pulchra:0.005907063027,Thalictrum_alpinum:0.005907063027):0.01303
576765):0.02311654394):0.01902312549):0.07471264185,((((Carduus_acanthoides:0.007841149
843,Saussurea_japonica:0.007841149843):0.02556902093,(Kalimeris_indica:0.02395052242,(Het
eropappus_crenatifolius:0.01171276016,(Aster_flaccidus:0.006084574753,Erigeron_breviscapus:
0.006084574753):0.005628185404):0.01223776227):0.00945964835):0.03064383078,(Cirsium_e
riophoroides:0.04179144149,(Aster_tataricus:0.02734148567,((Aster_fuscescens:0.00678536325
5,Saussurea_populifolia:0.006785363255):0.009152338626,(Saussurea_iodostegia:0.0097007828
71,((Aster_diplostephioides:0.001416729035,Saussurea_salsa:0.001416729035):0.003947730804,
(Saussurea_obvallata:0.003350815851,Saussurea_runcinata:0.003350815851):0.002013643988):0.
004336323032):0.006236919011):0.01140378379):0.01444995582):0.02226256006):0.01051295
265,(Saussurea_katochaete:0.0651315636,(((Saussurea_chingiana:0.01455871595,(Cirsium_shan
siense:0.004885582805,Cremanthodium_daochengense:0.004885582805):0.009673133145):0.01
814899962,((Valeriana_officinalis:0.01207370266,(Aster_ageratoides:0.004636021638,Lactuca_t
atarica:0.004636021638):0.007437681021):0.01380042322,(Saussurea_laciniata:0.01363636364,
Saussurea_thoroldii:0.01363636364):0.0122377622 4):0.006833589694):0.01969127433,((((Aster
_asteroides:0.001223776224,Saussurea_thomsonii:0.001223776224):0.009614858086,(Aster_stra
cheyi:0.001748251748,Cremanthodium_discoideum:0.001748251748):0.009090382562):0.01687
378385,((Saussurea_phaeantha:0.01049535155,(Aster_falcifolius:0.004434355498,Aster_tongole
nsis:0.004434355498):0.006060996048):0.00631647621,((Artemisia_abaensis:0.003907583382,
Aster_veitchianus:0.003907583382):0.01130168027,(Elatostema_monandrum:0.007368113838,(
Aster_likiangensis:0.004919073587,(Saussurea_erubescens:0.0001748251748,Saussurea_stella:0.
0001748251748):0.004744248412):0.002449040251):0.007841149815):0.001602564103):0.0109
005904):0.01448082545,((Thalictrum_delavayi:0.01935224389,(Saussurea_caudata:0.007135774
771,(Artemisia_verbenacea:0.005255999747,Aster_dolichopodus:0.005255999747):0.001879775
024):0.01221646912):0.01166085271,(Aster_poliothamnus:0.02046583758,Tripolium_pannonicu
m:0.02046583758):0.01054725902):0.01118014701):0.01020574629):0.0127325737):0.00943539
0604):0.06122818776):0.03481427709):0.08969004883,(((Polygonum_fertile:0.06159908704,((T
araxacum_albiflos:0.01762399757,(Anemone_rupestris:0.004807692308,Leontopodium_nanum:0.
004807692308):0.01281630526):0.0145546002,(Leontopodium_stoloniferum:0.02594833534,(Po
lygonum_delicatulum:0.01754026601,(Leontopodium_pusillum:0.01008827508,((Leontopodium
_ochroleucum:0.002622377622,Polygonum_filicaule:0.002622377622):0.006639675609,(Polygo
num_sparsipilosum:0.002636541993,(Leontopodium_himalayanum:0.0001456876457,Leontopod
ium_souliei:0.0001456876457):0.002490854347):0.006625511239):0.0008262218516):0.007451
990928):0.008408069326):0.006230262426):0.02942048927):0.084216737,((Dichrocephala_bent
hamii:0.06219857609,(Aster_argyropholis:0.03821374733,Pellionia_heteroloba:0.03821374733):

0.02398482876):0.04507523105,((Petasites_japonicus:0.03575122172,(Polygonum_strindbergii:0.
02028234823,(Polygonum_suffultum:0.0116290286,(Leontopodium_conglobatum:0.0053284867
98,Polygonum_wallichii:0.005328486798):0.006300541805):0.008653319625):0.01546887349):
0.03346380352,((((Aster_vestitus:0.01067305354,Kalimeris_longipetiolata:0.01067305354):0.01
092657343,((Pilea_salwinensis:0.001562416708,Polygonum_jucundum:0.001562416708):0.0101
981352,(Elatostema_pseudoficoides:0.00809071101,(Persicaria_barbata:0.004197291971,Valeria
na_jatamansi:0.004197291971):0.003893419039):0.003669840896):0.009839075059):0.0190773
2496,((Persicaria_sagittata:0.01263467778,(Artemisia_lactiflora:0.003310668456,Aster_albescen
s:0.003310668456):0.009324009324):0.01397836966,((Valeriana_hardwickii:0.00897067794,(Pol
ygonum_japonicum:0.005099067599,Urtica_atrichocaulis:0.005099067599):0.003871610341):0.
01454278154,(Artemisia_robusta:0.01331532429,(Boehmeria_clidemioides:0.005474174444,(Art
emisia_codonocephala:0.00307360493,(Adenocaulon_himalaicum:0.0004370629371,Fagopyrum
_acutatum:0.0004370629371):0.002636541993):0.002400569514):0.007841149843):0.01019813
52):0.003099587954):0.01406390448):0.01618892981,((Leontopodium_artemisiifolium:0.02249
364597,(Aster_lavandulifolius:0.00981882079,(Potentilla_glabra:0,Rosa_mairei:0):0.0098188207
9):0.01267482517):0.02499047791,(((Anemone_rivularis:0.01319728637,(Anaphalis_nepalensis:
0.005073084547,Triplostegia_glandulifera:0.005073084547):0.00812420182):0.009365926781,(
Anaphalis_aureopunctata:0.01529897283,((Leontopodium_calocephalum:0.003857445971,Persic
aria_vivipara:0.003857445971):0.005398766205,(Anemone_flaccida:0.007062574176,(Anemone
_baicalensis:0.002289188235,Anemone_rupicola:0.002289188235):0.004773385942):0.0021936
37999):0.006042760656):0.007264240316):0.0157324957,((Elatostema_obtusum:0.00641857972
4,Sanguisorba_filiformis:0.006418579724):0.01887743785,((Achillea_acuminata:0.00555029490
6,Polygonum_taquetii:0.005550294906):0.01005244755,(Leontopodium_stracheyi:0.0087554231
11,Polygonum_rigidum:0.008755423111):0.006847319347):0.009693275113):0.01299969128):0.
009188415027):0.009381757853):0.01234914352):0.03805878189):0.0385420169):0.063147959
79,(((Koenigia_islandica:0.05216378825,(Circaeaster_agrestis:0.02572475719,Halerpestes_sarme
ntosa:0.02572475719):0.02643903105):0.04421449872,((((Laportea_bulbifera:0.005827505828,L
aportea_cuspidata:0.005827505828):0.01017215217,(Pilea_melastomoides:0.01067878229,Rume
x_crispus:0.01067878229):0.005320875707):0.02855477855,((Pilea_angulata:0.01731084681,(Ar
temisia_fulgens:0.002768065268,Fallopia_convolvulus:0.002768065268):0.01454278154):0.0199
7514186,((((Fallopia_dentatoalata:0.001562416708,Polygonum_pubescens:0.001562416708):0.0
04780042554,(Rumex_acetosa:0.002776388582,Rumex_dentatus:0.002776388582):0.003566070
68):0.0101981352,((Rheum_alexandrae:0.00203962704,(Procris_crenata:0.0005827505828,Rume
x_trisetifer:0.0005827505828):0.001456876457):0.007062574176,(Pilea_auricularis:0.004224941
725,(Pilea_martini:0.001456876457,Rumex_japonicus:0.001456876457):0.002768065268):0.004
877259491):0.007438393244):0.01178055733,(((Artemisia_dubia:0.004384793741,(Adenostemm
a_lavenia:0,Urtica_laetevirens:0):0.004384793741):0.006042760656,(Achudemia_japonica:0.007
986837489,Elatostema_balansae:0.007986837489):0.002440716909):0.01271840933,(Artemisia_
calophylla:0.01048951049,(Maoutia_puya:0.005099067599,Polygonum_perfoliatum:0.00509906
7599):0.00539044289):0.01265645324):0.005175188062):0.008964836884):0.007268447879):0.
0261434482,((Persicaria_hydropiper:0.02671858769,(Polygonum_aviculare:0.01666614014,Poly
gonum_patulum:0.01666614014):0.01005244755):0.02403846154,((Pilea_pauciflora:0.01028186
675,(Elatostema_parvum:0.002185314685,Polygonum_sibiricum:0.002185314685):0.008096552
066):0.02095303793,(Dichrocephala_integrifolia:0.01274439236,Elatostema_ficoides:0.0127443

9236):0.01849051232):0.01952214455):0.01994083553):0.02568040221):0.07897275258,(((Arte
misia_phaeolepis:0.01978753676,Artemisia_przewalskii:0.01978753676):0.03823496279,(((Poly
gonum_milletii:0.005404607261,Rumex_angulatus:0.005404607261):0.01819202378,(Ligularia_
cyathiceps:0.008552546937,Ligularia_longifolia:0.008552546937):0.0150440841):0.0205472856
1,(Ligularia_curvisquama:0.02813365002,(Artemisia_vulgaris:0.007289072187,(Artemisia_hedin
ii:0.002397648326,Artemisia_leucophylla:0.002397648326):0.004891423861):0.02084457784):0.
01601026662):0.01387858291):0.07263917666,(Aster_trichoneurus:0.09731934732,Cremanthodi
um_calcicola:0.09731934732):0.03334232889):0.04468936333):0.03361274428):0.05133568406)
:0.04844179086):0.1003496503):0.01136363636):0.01136363636):0.01136363636):0.030375874
13):0.02644230769):0);
