# Supplementary material for: Large-scale geographic patterns and environmental and anthropogenic drivers of wetland plant diversity in the Qinghai-Tibet Plateau
Source: BMC Ecol Evol. 2024 Jun 3;24:74. doi: 10.1186/s12862-024-02263-w (PMC11145778; doi:10.1186/s12862-024-02263-w)
Supplement: Supplementary file 6 — Supplementary Material 6 [file 12862_2024_2263_MOESM6_ESM.docx]

Additional file 6


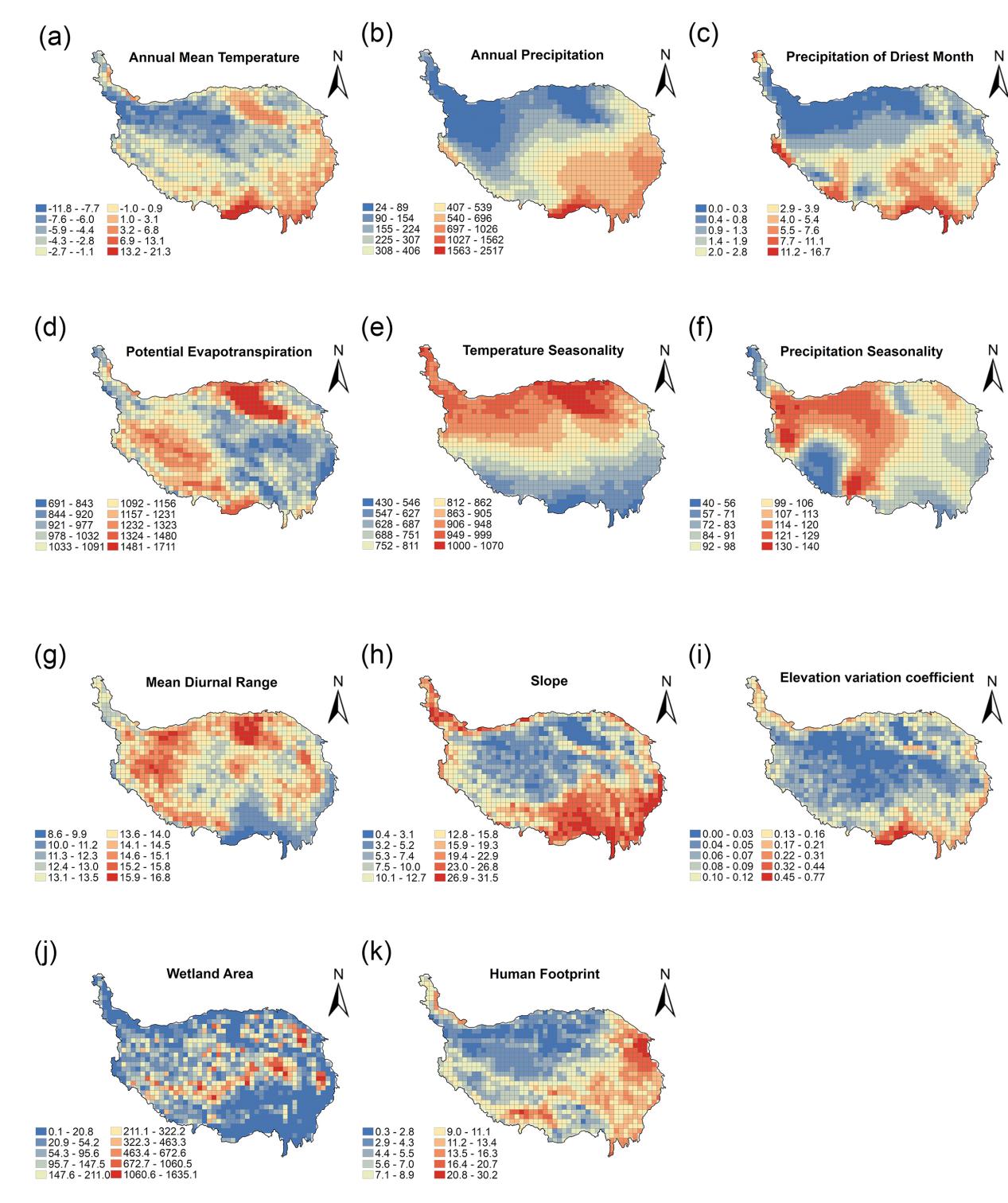


Geographic patterns of environmental variables used in this study. (a) annual mean temperature (AMT), (b) annual precipitation (AP), (c) precipitation of driest month (PDM), (d) potential evapotranspiration (PET), (e) temperature seasonality (TS), (f) precipitation seasonality (PS), (g) mean diurnal range (MDR), (h) slope (Slope), (i) elevation variation coefficient (EVC), (j) wetland area (WA), (k) human footprint (HF)
